# Dual framework for rainfall prediction: a multi-seed machine and deep learning evaluation across Pakistan’s climatic regimes

**DOI:** 10.1038/s41598-026-50979-0

**Published:** 2026-04-29

**Authors:** Hira Farman, Muhammad Arif Hussain, Sarang Shaikh, Saif Hassan, Syed Rizwan Hassan

**Affiliations:** 1 Department of Computer Science, Karachi Institute of Economics and Technology (KIET), Karachi, 75190 Pakistan; 2 Department of Information Security and Communication Technology, Norwegian University of Science and Technology (NTNU), Gjøvik, 2815 Norway; 3 Department of Computer Science, Sukkur IBA University, Sukkur, 65200 Pakistan; 4 Department of Computer Engineering, Gachon University, Seongnam-si, 13120 South Korea

**Keywords:** Rainfall classification, Particle swarm optimization (PSO), Variational mode decomposition (VMD), Time-series cross-validation, SMOTE, Class weighting, Multi-horizon, Climate sciences, Environmental sciences, Hydrology, Natural hazards

## Abstract

Accurate prediction of rainfall events is vital for agriculture, hydrology, flood preparedness, and climate-adaptive strategies in regions of Pakistan susceptible to monsoons and droughts. Increasing climate variability highlights the need for reliable data-driven forecasting systems capable of precisely representing nonlinear atmospheric dynamics across different forecast intervals. This study introduces an extensive hybrid machine learning–deep learning (ML–DL) framework designed to classify daily rainfall events (rain vs. no rain) and predict several horizons (RainDay0–RainDay5) by utilizing 130,230 daily meteorological data collected from 10 geographically diverse cities in Pakistan between 1990 and 2025. Machine learning models, such as Extra Trees classifier, Histogram Gradient Boosting, Ridge Classifier, and Gaussian Naïve Bayes, underwent evaluation using leakage-free TimeSeriesSplit validation with SMOTE applied only to training folds using various seeds.To improve the quality of sequence representation, Variational Mode Decomposition (VMD) was utilized to extract band-limited intrinsic mode functions from meteorological signals, while Particle Swarm Optimization (PSO) was employed for adaptive hyperparameter tuning and genetic algorithms were used. Experimental findings indicate that ensemble ML models delivered robust baseline classification results across various cities, while VMD-augmented deep learning frameworks enhanced robustness and temporal learning consistency throughout different forecast horizons. Specifically, VMD–LSTM demonstrated robust performance in Hyderabad (Acc = 0.862, F1 = 0.871, AUC = 0.941), whereas VMD–GRU exhibited dependable discrimination ability in Jamshoro (AUC = 0.918), Quetta (AUC = 0.867), and Thatta (AUC = 0.896).The enhanced VMD–PSO–GRU (VPG) framework demonstrated superior rainfall classification performance across various cities, particularly excelling in Hyderabad (AUC = 0.912), Jamshoro (Acc = 0.882), and Thatta (Acc = 0.878). In multi-horizon forecasting, a proposed multi-head VMD–GRU–Attention model, improved by hybrid PSO–GA, provided the most reliable results across forecast lead times RainDay0–RainDay5. In short-term forecasting, the system achieved an Accuracy of 0.872 (RainDay0) in Hyderabad, while maintaining steady medium-term performance at RainDay3 (approximately 0.819 for Hyderabad; about 0.808 for Thatta) and long-term predictions near 0.80 at RainDay5 across Gharo, Thatta, and Mirpur Khas, showcasing resilience despite increasing forecast uncertainty. The proposed hybrid framework demonstrates improved stability in temporal learning, robustness against noise, and capacity to generalize across areas, validating its effectiveness for operational rainfall early-warning systems in various climatic settings of Pakistan.

## Introduction

Classification of rainfall refers to the task of predicting rainfall in a specific region. Proper rainfall forecasting provides the decision-makers incredibly good information that helps them to strategize resources better, reduce risks, and generate better socio-economic outcomes. It is also imperative because more accurate rainfall predictions can assist farmers in scheduling the crop, cities in preparing against possible future flood risks, and the emergency responders in planning against the severe disaster. There are a number of ways of predicting rain as explored in the literature, such as statistical models, satellite imagery, and computer simulations. The techniques use variables like temperature, humidity, wind patterns, atmospheric pressure, etc., to predict short-term weather forecasts and long-term predictions of climatic factors.

Various datasets and methods have been used to predict rainfall at different temporal scales. Rainfall prediction may be categorized into several groups depending on the dataset used, the type of models employed, and the frequency of data collection, including very short-term, short-term, and long-term forecasts^1^ In hydrology, agriculture, and disaster management, rainfall prediction is a critical concern because it supports water resource planning, crop yield estimation, and flood-risk reduction^2^ For china city Weifang multiple model compare for climate prediction^3^

According to Elahi et al.^4^, statistical forecasting models including Autoregressive Integrated Moving Average (ARIMA), Seasonal Autoregressive Integrated Moving Average (SARIMA), and other regression-based methods were widely employed for precipitation modeling in previous decades. The strong nonlinearity, chaotic behavior, and seasonal fluctuations present in rainfall time series are difficult for these models to capture because, despite their mathematical simplicity and interpretability, they typically assume that the underlying data are linear, stationary, and normally distributed^5^. Furthermore, these models are less useful in situations when temperature, humidity, wind speed, and surface pressure interact intricately to affect precipitation since they find it difficult to integrate various meteorological predictors Arshad et al.^6^

As a result, the purely statistical techniques frequently fail in the areas, which have semi-arid climate, coastal variability, and localized convective rains, such as most parts of Pakistan. Machine learning methods have thus become more competitive and often outperform the conventional statistical models. Random Forest (RF), Support Vector Machines (SVM), Logistic Regression (LR), and Gradient Boosting (GB) are the models that can model nonlinear relationships between meteorological predictors and provide better prediction accuracy than classical statistical models at the same time^7^. The methods are scalable, interpretable and can be applied in high-dimensional meteorological datasets, which is appealing in operational rainfall forecasting applications.

However, ML models have significant drawbacks in addition to these advantages. They frequently rely on static input-output mappings and are unable to fully utilize the sequential linkages found in lengthy historical time series. They are therefore limited in their capacity to capture temporal dynamics, including autocorrelations, seasonal cycles, and lag effects. Furthermore, their effectiveness frequently decreases in highly imbalanced datasets with many dry days and few wet days, necessitating the use of additional techniques like class weighting or the Synthetic Minority Over-Sampling Technique (SMOTE) to produce accurate classification results. These drawbacks have led to a rise in the use of deep learning techniques, which are built to handle sequential data and long-term temporal structures.

More reliable options include deep learning models, especially when dealing with time-series forecasting problems. Because of their ability to retain long-term temporal connections and sequential patterns, LSTM, BiLSTM, and Gated Recurrent Unit (GRU) models have shown great efficacy in rainfall prediction. Among recurrent designs, GRU networks stand out for their higher computational efficiency, more straightforward structure than LSTM, and potent capacity to handle long-range dependencies and vanishing gradients. For large-scale climate datasets, when quicker convergence and simpler models are crucial, these properties are especially helpful. Deep learning models, such those described by Wani et al.^8^, have recently shown better results in rainfall predicting challenges. By using attention mechanisms to understand long-range dependencies across both space and time, transformer-based structures have made progress in the field (Elahi et al.^9^).

Although these advances have taken place, there are a number of challenges that remain. Most previous studies utilized coarse-resolution data or only satellite based precipitation data like TRMM and GPM-IMERG Arshad et al.^6^ that are not usually adequate to reflect local precipitation variability in semi-arid and coastal areas^10^. Prediction bias is also present in the nature of rainfall datasets whereby dry days far outnumber rainy days, which also leads to prediction bias where models will prefer the majority category. Moreover, the majority of past studies have employed either single-seed or single-fold validation methods, which casts doubts on the robustness, reproducibility, and generalizability (Javid et al.^11^).

Quetta and Mirpur Khas (deltaic and agrarian zones with monsoon-driven rainfall), Hyderabad, Jamshoro, and Nawabshah (semi-arid interior regions of Sindh with high temperature variability), Larkana (hot semi-arid with episodic rainfall), Quetta (arid highlands with continental extremes), and Hub–Lasbela in Balochistan (a dry coastal plateau with sparse precipitation) all represent distinct climatic regimes across Pakistan. Existing studies support this climatic diversity. In order to capture both nonlinear spatial dependencies and temporal dynamics, many recent research highlight the combination of machine learning and time-series forecasting models^12^. This allows for more reliable and broadly applicable rainfall prediction across diverse climatic regimes.

In addition, climate projection Sharifi et al.^13^ studies based on CMIP6 multi-model ensembles combined with artificial neural network downscaling approaches have been used to estimate long-term precipitation and temperature changes across major Pakistani cities under different Shared Socioeconomic Pathway (SSP) scenarios. However, these approaches primarily address long-term climate variability rather than operational daily rainfall occurrence classification.

Metaheuristic methods of optimization have increasingly been of attentiveness to hydrological and climate prediction problems since they provide effective search methods in high-dimensional hyperparameter space that is usually challenging to optimize with more traditional grid search or random search approaches. One of such methods is Particle Swarm Optimization (PSO), which has proved to have high convergence, relatively low complexity, and the ability to search the globe during rainfall and hydrological forecasting. To give an example, Nemade et al.^14^ experimented with an LSTM network where M-PSO was used to optimize and resulted in better rainfall prediction^43^ demonstrated that PSO-SVR could be successfully applied to short-term rainfall prediction. Similarly, Zhou et al.^35^ highlighted the effectiveness of using decomposition-based preprocessing and state-of-the-art sequence modeling in rainfall time-series prediction. These findings suggest that predictive stability and generalization are PSO-enhanceable, particularly when dealing with noisy, nonlinear, and heterogeneous climatic data. The processes of rainfall are nonlinear and non-stationary and very sensitive to the establishment of models; therefore, PSO is used in the present research to optimize machine learning and deep learning models systematically. This enables a more plausible comparison of the modeling paradigms and strengthens the different climatic regimes in Pakistan.

Beyond rainfall forecast model enhancements based on optimization Recent developments in artificial intelligence have shown how well deep learning architectures foresee a variety of climatic and environmental factors that affect the behavior of extreme weather. In particular, hybrid deep learning frameworks, transformer-based models, and explainable artificial intelligence techniques have significantly improved both predictive accuracy and interpretability in time-series forecasting systems across climate-sensitive applications. These developments highlight the growing role of advanced data-driven modeling approaches in enhancing environmental forecasting performance beyond traditional optimization-based rainfall prediction methods. The ability to forecast fine particulate matter (PM_2.5_) concentrations with hybrid convolutional neural network-based structures has been found to be highly effective in data-driven architectures to model nonlinear processes in the atmosphere that affect extreme weather variability as presented by Inam et al.^15^. In a similar manner, forecasting models, which utilize transformers and are combined with explainable artificial intelligence methods, have enhanced interpretability and predictive accuracy of large-scale environmental monitoring systems, which can be used to inform decision-making in climatically sensitive areas^16^. PM_1.0_ forecasting and global air-quality exposure prediction based on stacked deep learning architectures and hierarchical ensemble learning strategies have both been successfully applied and show the benefits of combining multiple predictors with feature-attribution mechanisms in atmospheric time-series modeling^17–19^.

Besides data-driven models, physics-informed neural networks (PINNs) have recently become effective instruments to predict meteorological variables including temperature, humidity, and wind speed by directly relating physical constraints within the deep learning frameworks, and by enhancing predictive accuracy across heterogeneous climatic conditions^20–22^. These methods contribute to better generalization of models by integrating the domain knowledge in the process of learning and this is especially useful in predicting the cases of hydro-meteorological extremities. More recently, physics-informed deep learning models are also applied to rainfall prediction in a variety of climate conditions, which have shown better robustness than the traditional methods of purely data-driven rainfall forecasting^23^.

Despite these advances, most existing studies primarily focus on regression-based prediction of environmental variables such as air quality, temperature, humidity, and wind speed rather than binary rainfall occurrence classification. Although recent deep learning and physics-informed frameworks have demonstrated strong performance in forecasting meteorological parameters and atmospheric pollutants, comparatively limited research has addressed multi-horizon daily rainfall occurrence prediction within a unified classification framework. In particular, the combined application of signal decomposition techniques such as Variational Mode Decomposition (VMD), hybrid metaheuristic optimization strategies including PSO–GA, multi-seed TimeSeriesSplit-based temporal validation, and city-wise statistical significance testing across heterogeneous climatic regimes remains largely unexplored. Furthermore, the role of explainable artificial intelligence methods in interpreting predictor importance across short-, medium-, and extended lead-time rainfall occurrence forecasts (RainDay0–RainDay5) has not been systematically investigated for operational early-warning and climate-resilience decision-support applications. Addressing these limitations, the present study proposes a robust Hybrid framework rainfall occurrence classification strategy designed to improve predictive reliability, interpretability, and cross-regional generalization across diverse climatic environments of Pakistan.

The suggested framework presents a leakage-controlled multi-head VMD–GRU–Attention–PSO–GA architecture for simultaneous multi-horizon rainfall occurrence forecasting across heterogeneous climatic regimes, in contrast to previous rainfall occurrence classification studies that are restricted to single-horizon prediction.

This study is guided by the following research questions:

### RQ1

How do machine learning models compare in daily rain/no-rain classification under identical time-series cross-validation settings across multiple climatic regimes of Pakistan?

### RQ2

How effectively do deep learning models perform in multi-horizon rainfall occurrence prediction (RainDay0 to RainDay5) under identical temporal validation settings?

### RQ3

Does signal denoising using Variational Mode Decomposition (VMD) improve the classification performance of sequential deep learning models for rainfall occurrence prediction?

### RQ4

What is the impact of hybrid Particle Swarm Optimization and Genetic Algorithm (PSO–GA)–based hyperparameter optimization on predictive stability and generalization across heterogeneous climatic regions?

### RQ5

How robust, reproducible, and statistically significant is the proposed rainfall occurrence classification framework across multiple Pakistani cities when evaluated using multi-seed TimeSeriesSplit-based temporal cross-validation and city-wise nonparametric statistical significance tests (Friedman and Wilcoxon signed-rank tests)?

### RQ6

How can Explainable Artificial Intelligence techniques, particularly SHAP analysis, be used to interpret feature importance across multi-horizon rainfall occurrence predictions (RainDay0 to RainDay5) and improve the transparency and reliability of classification models for short-, medium-, and extended lead-time forecasting?

### Objectives of the study

Rainfall occurrence classification can be defined as the process of identifying the probability of a particular region to have rainfall at a certain time horizon. Rainfall classification is essential in disaster preparedness, crop scheduling, water management, and urban planning that is resilient to climate change. Reproducible short- to medium-term forecasts on the occurrence of rainfall can assist policymakers, farmers and emergency management agencies to adopt proactive actions to extreme weather events and hydrometeorological hazards.

This study pursues the following objectives:

1. To construct a two-framework experimental set-up, which systematically assesses machine learning (ML) frameworks to identify single-day rainfall occurrence classification and sequential deep learning (DL) frameworks to identify both single-day and multi-horizon rainfall occurrence prediction (RainDay0-RainDay5) in ten climatically varied Pakistani cities that represent coastal, deltaic, semi-arid and highland.
2. To compare the performance of sequential deep learning models (LSTM, BiLSTM, GRU, and Transformer), in their default settings, and following PSO-based hyperparameter optimization, to model nonlinear temporal rainfall dynamics, and to compare their results with benchmark machine learning models (RF, LR, GB, SVC), ensemble learners (ET, HGB), and lightweight baselines (Ridge Classifier).
3. To partition the datasets of rainfall occurrences in case of class imbalance by using SMOTE in ML pipelines and class-weighted learning approaches in DL models without breaking the temporal correlations using TimeSeriesSplit-based multi-seed cross-validation.
4. To achieve better predictive performance with signal decomposition and hybrid optimization, apply Variational Mode Decomposition (VMD) to reduce noise and PSO-GA-based hyperparameter optimization to optimize stability and generalization of sequential rainfall classification models.
5. To evaluate model robustness and statistical reliability across multiple climatic regimes using multi-seed TimeSeriesSplit validation together with city-wise nonparametric statistical significance testing (Friedman and Wilcoxon signed-rank tests).
6. To interpret model behavior using Explainable Artificial Intelligence techniques, particularly SHAP-based feature attribution, for analyzing meteorological predictor importance across short-, medium-, and extended lead-time rainfall occurrence forecasts (RainDay0–RainDay5).
7. To analyze performance trade-offs across climatic environments, including accuracy, recall (false-alarm control), PR-AUC, ROC-AUC, and temporal stability, in order to identify the most reliable modeling strategies for operational rainfall classification in heterogeneous hydro climatic regions of Pakistan.
8. To support climate-resilient decision-making and early-warning applications by identifying an operationally deployable rainfall occurrence classification framework suitable for flood preparedness, agricultural planning, water resource management, and sustainable urban infrastructure development.
9. To evaluate the operational reliability of rainfall occurrence classification models using complementary performance indicators such as PR-AUC, ROC-AUC, false alarm rate, miss rate, specificity, negative predictive value (NPV), and calibration-based reliability metrics in order to assess their suitability for early-warning and decision-support applications across diverse climatic regimes.

### Sustainability and policy implications

The implementation of this study serves various goals of the United Nations Sustainable Development Goals (SDGs) in that it entails the integration of scientific rigor in a sustainability-oriented framework. By addressing SDG 2 (Zero Hunger) through the correct classification and prediction of rainfall, the suggested ML-DL framework increases food security at the community level by enhancing agricultural planning and irrigation timetables. It relates to SDG 11 (Sustainable Cities and Communities) because it helps to transmit early-warning data on flood preparedness and enhance city resilience to extreme weather conditions. Moreover, the study contributes to SDG 13 (Climate Action) by improving the adaptive capacity on the local level, as well as encouraging evidence-based decision-making in climate-prone areas. The combination of contributing to evidence-based policymaking on climate-resilient agriculture, water management, and disaster-risk reduction can support the efforts to address the problem in the various climatic zones of Pakistan^24^.

The structure of this article is as follows. The section on related work examines current techniques for predicting and detecting rainfall occurrences. The dataset, preparation procedures, and the suggested multi-horizon rainfall occurrence classification framework based on the PSO–GA adjusted VMD–GRU–Attention hybrid model are all covered in the Materials and Methods section. The experimental setting, model topologies, evaluation metrics, and SHAP-based explainability analysis for feature importance interpretation are all explained in the Final Prediction Model section. Performance comparisons across forecast horizons (RainDay0–RainDay5), statistical testing, and cross-regional reliability analysis are presented in the section titled Experimental Design, Results, and Evaluation. The Conclusion section concludes by summarizing the key findings and possible directions for future investigation.

### Background study

Rainfall prediction is a major issue in hydrology, agriculture, and disaster management, and multiple studies have utilized machine learning (ML) and deep learning (DL) models to enhance the accuracy of prediction. To illustrate, He et al.^1^ introduced an attention-based encoder-decoder deep learning model of multi-step monthly rainfall forecast, where the predictive performance and interpretability are enhanced by using feature-attribution analysis, but the architecture lacks metaheuristic hyperparameter optimization strategies to enhance convergence stability.

Conventional ML models like the Random Forest (RF), the Support Vector Machines (SVM), and the Gradient Boosting (GB) have demonstrated a high potential to model nonlinear, multi-dimensional interactions between meteorological predictors and both rainfall outcomes^2^. The advantage of these models is that they can be interpreted and are also computationally efficient. More modern DL models, including Long Short-Term Memory (LSTM), Bidirectional LSTM (BiLSTM), and Gated Recurrent Units (GRU), can utilize time-varying information of climatic data sequential dependencies. These DL models have shown better capability to represent complex spatiotemporal dynamics and long term rain patterns compared to traditional ML baselines. As an example, LSTM and BiLSTM networks are useful in bidirectional time-dependent modeling, whereas GRUs can be provided with comparable accuracy at a lower number of parameters and are therefore better applied to medium-sized datasets^3,4^. Although these advances have been made, there are a number of limitations present in the literature. First, most previous analyses were based on coarse-resolution or satellite-only data, such as TRMM or GPM products^5,6^. Although useful on the global levels, these datasets do not tend to represent the localized rainfall variability, especially in semi-arid and coastal areas where rainfall is seldom, but when it occurs, it can significantly influence the outcome. Second, the rainfall data is usually heavily misbalanced towards classes; it is only a small proportion of rainy days in comparison to dry days. The result of this imbalance frequently causes models to support the dominant (dry) class which amplifies overall accuracy but fails at detecting actual rainfall events. Third, some studies performed an evaluation with the single-seed experiment or small fold validation^7^, which compromises reproducibility and does not support the study of model robustness across initializations. An alternative common trend is that the models are not trained and validated across cross-city or region-specific analyses thus failing to consider the local climatic heterogeneity, which lowers outside generalizability to the various geographical situations.

While highlighting the growing role of hybrid machine learning and time-series fusion frameworks to improve generalization and operational reliability across diverse climatic regimes, recent surveys and regional studies also highlight the strong spatial heterogeneity of rainfall across Pakistan and neighboring highland regions, as well as the rapid evolution of deep learning and foundation models for weather forecasting^10,11,8,9,12^. Models include the study by Shah and Sharifi et al.^13^ that predicts future precipitation and temperature of large cities in Pakistan using CMIP6 based multi-model ensembles (MMEs) and an ANN downscaling method, and aimed primarily at forecasting long-term climatic change scenarios at different geographical contexts.

Rajab et al.^25^ applied ML to flood forecasting in Bangladesh, yet all their results were based on crude data and the interest in floods, which restricted the applicability.

Farman et al.^26^ compared DL and ML on precipitation of Australia and found that DL was stronger but their one-seed experiment and combined data weakened their study. Our research builds on them by employing a two-layer ML-DL structure in ten cities in Pakistan with multi-seed, multi-fold validation and class imbalance correction, so that it is reproducible and region-specific.

Wani et al.^27^ compared the precision of the rainfall prediction in the North-Western Himalayas on the basis of machine-learning, deep-learning, and time-series models but their research was limited to an altitudinal gradient. Their method is expanded to ten cities with different climatic conditions in Pakistan, making our framework apply VMD-PSO-optimized GRU models, with greater spatial generalization and relevance to operations.

Nemade et al.^14^ adopted an LSTM-M-PSO hybrid to improve the accuracy of rainfall prediction, though they only evaluated their model on one regional dataset as well as on tuning parameters. We build on this, including PSO in a VMD-denoised GRU model validated in a variety of Pakistani climates to achieve greater generalizability. Zhang et al.^28^ have used quadratic decomposition and enhanced algorithm to forecast monthly precipitation but the model was confined to monthly and synthetic data. We slice the concept of decomposition to everyday sequence by VMD, enhancing both temporal granularity and operative application.

Hasany et al.^29^ compared a few ML models in predicting rainfall in Bangladesh and Pakistan but their evaluation was based on single-split evaluation. Our work enhances this as it uses a multi seed, time-series cross-validation method to assess model robustness and reproducibility. Guo et al.^30^ benchmarked deep learning systems to climate forecasting in China, but the research failed to touch on the issue of class imbalance and the existence of decomposition noise. We use Synthetic Minority Over-Sampling Technique and Variational Mode Decomposition in our model to improve the quality of signals and minority-class recall.

Waqas et al.^31^ suggested to use hybrid deep learning models to predict rainfall in the tropics, however, with a greater focus on model stacking. Expanded this stream of literature by introducing multiapproach-Framework, combining swarm optimization (PSO) and VMD-based preprocessing that will allow the framework to be more versatile and transferable to semi-arid areas. The previous research has shown that there has been a significant advance in the detection, classification and prediction of rainfall through statistical and artificial intelligence methods. Hernandez et al.^32^ demonstrated that deep learning and classical machine learning models could effectively predict nonlinear rainfall patterns but their models were mostly tested at a small time scale and failed to explicitly assess cross-regional generalizability. Zeyaeyan et al.^33^ and Nabeel and Athar^34^ were interested in the classification of precipitation regime and warning systems, but their approaches were based on the rule-based or multi-criteria decision modeling, which might not be able to scale with highly non-stationary climatic conditions.

Iqbal et al.^35^ and Arshad et al.^36^ were also informative in understanding the variability of rainfall in the region, and the assessment of satellite-based precipitation products, but they did not build predictive machine learning pipelines to predict rainfall occurrence in real time or the short term. Rasool et al.^37^ created flood inundation mapping machine learning models using rainfall in Karachi, Pakistan. More recent machine learning and deep learning systems to map urban floods and predict multi-step rainfall have also claimed better predictive accuracy, though they may not have rigorous time-series testing, class-imbalance, and explainability mechanisms that are needed in operational early-warning systems.A modified genetic algorithm (MGA) to optimize the hyperparameters of a multilayer perceptron (MLP) model to classify rainfall was proposed by Marji et al.^38^, with classification accuracies of 86.02 and 79.05 on the Australian and Indonesian BMKG datasets respectively. Nevertheless, the MGAMLP framework is based on the single-step classification of rainfalls and lacks the multi-horizon sequence learning. Farman et al.^39^ also examined the effects of activation functions on the performance of rainfall prediction with various machine learning and deep learning architectures and showed that the correct choice of activation functions can enhance the accuracy of the model and convergence stability.

Farman and Hasany^40^ evaluated ARIMA and VAR models for rainfall forecasting at the national scale; however, these statistical models are constrained by linear assumptions, limited capability for modeling multiscale atmospheric variability, and lack of sequence-learning mechanisms. Moreover, the study did not incorporate multi-horizon rainfall occurrence prediction, signal decomposition techniques such as VMD, evolutionary optimization strategies, or explainable AI-based interpretation, which are addressed in the present hybrid framework.

The effects of climate change on agriculture, food security, and sustainable development were examined by Saleem et al.^24^, who identified policy-level vulnerabilities without connecting these findings to practical, city-scale rainfall prediction systems. Praveena et al.^41^ demonstrated baseline classification performance using logistic regression and support vector machines for rainfall prediction; however, the study does not address time-aware validation or model generalization across varied climatic regimes.

Azmi et al.^42^ used Naive Bayes classification in predicting rainfall in Banyuwangi which showed that probabilistic learning could be applied to predict rainfall events but the framework used was tested on a geographically limited dataset and did not focus on generalization of the classes. Aderyani et al.^43^ introduced a hybrid PSO-SVR, LSTM, and CNN-based short-term predictions model which had the highest predictive accuracy, but the work was done in terms of continuous rainfall prediction and not operational binary rainfall classification and failed to compare the multi-city robustness and the time-aware cross-validation of the model across heterogeneous climatic regimes .Zhou et al.^44^ improved the prediction of nonlinear rainfall time series by combining multifractal analysis, recurrent neural networks(RNN), and Variational Mode Decomposition(VMD).

Recent innovations in deep learning have proposed various state-of-the-art time-series forecasting architectures, such as N-BEATS, ConvLSTM, Autoformer, Informer, and Temporal Fusion Transformer (TFT) models, which have performed well on long-sequence and multi-horizon prediction tasks on environmental and meteorological data. Indicatively, Oreshkin et al.^45^ introduced the N-BEATS architecture that offers interpretable representations of basis-expansion representations of univariate and multivariate prediction tasks and achieved competitive performance on benchmark time-series prediction problems. In the same way, Ghosh et al.^46^ designed a ConvLSTM-based model to predict short-term rainfall in four large Indian cities, and it is effective as it can predict spatio-temporal rainfall with understandable AI integration. Wu et al.^47^ proposed Autoformer that enhances the long-term forecasting performance by decomposing temporal signals into seasonal and trend components via transformer based on decomposition. Similarly, Zhou et al.^48^ suggested Informer, which is more efficient in terms of computations with long sequences to be forecasted with the help of sparse self-attention. More so, Lim et al.^49^ created Temporal Fusion Transformer (TFT), which allows interpretable multi-horizon prediction via attention-based variable selection and gating. Nevertheless, the majority of these architectures are primarily regression-based time-series forecasting and applied to few applications, such as multi-horizon binary rainfall occurrence classification with decomposition-based preprocessing and swarm-optimized sequence learning.

Addressing these gaps, the present study proposes a multiapproachframework rainfall occurrence classification approach that integrates machine learning (ML) and deep learning (DL) models for binary and multi-horizon rainfall prediction (RainDay0–RainDay5) across ten major Pakistani cities representing coastal, semi-arid, and arid climatic regimes. The framework has been based on 35 years of observation of daily meteorology (1990–2025) which consists of about 130,000 records giving it a sufficiently deep temporal structure to support strong sequence models. In contrast to most previous studies that primarily focus on regression-based rainfall forecasting, limited attention has been given to multi-horizon rainfall occurrence classification across heterogeneous climatic regimes .Moreover, model stability and regional generalization are tested on city-wise statistical significance tests and various complementary measures of performance, such as Accuracy, Precision, Recall, F1-score, and ROC–AUC. This combined framework also promotes interpretability over short-, medium-, and long-lead-time horizons through explainable modeling strategies hence enhancing predictive reliability and operational applicability of the localized rainfall early-warning decision support in different climatic settings of Pakistan.

**Fig. 1:**
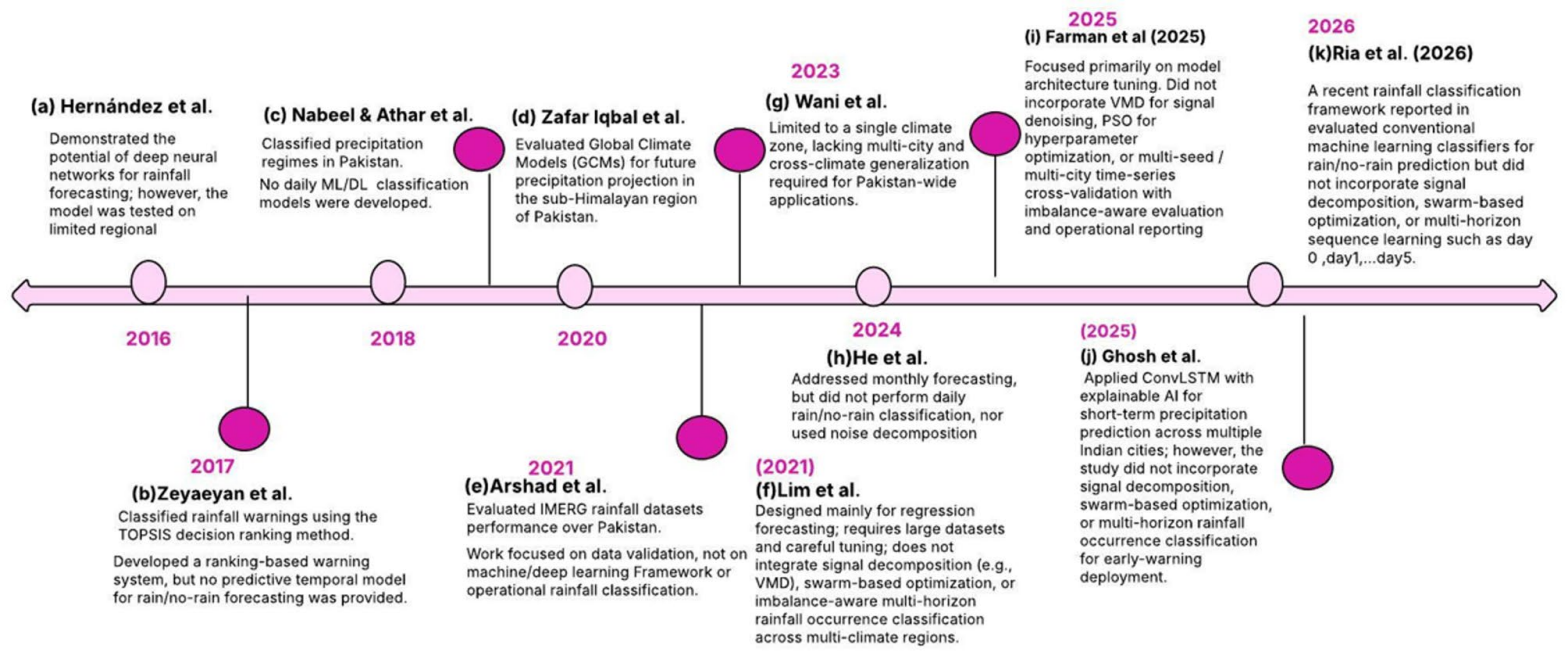
Chronological development of ML/DL-based rainfall prediction models and limitations leading to the proposed framework.

Figure 1 highlights the methodological evolution that underpins the proposed multi-horizon rainfall occurrence classification architecture by showing the transition from conventional statistical forecasting techniques to hybrid deep learning, decomposition-based preprocessing, and frameworks for metaheuristic optimization. A comparative analysis of the best rainfall prediction studies is shown in Table 1, which includes information on the datasets, approaches, regions covered, and limitations of each study. The analysis shows that integrated multi-horizon rain/no-rain classification frameworks with signal decomposition, hybrid optimization, and leakage-free temporal validation across various climatic circumstances are absent from the majority of earlier studies.

**Table 1:** Comparative analysis of state-of-the-art rainfall prediction models and research gaps addressed by the proposed framework.

Ref. no.	Study description	Dataset/data source	Cities/region	Key limitation	Key contribution
Hernández et al. (2016)	Deep learning for rainfall prediction.	Local rainfall time-series.	Not Pakistan-specific.	No rain/no-rain classification; no VMD; no PSO; limited generalization.	Introduced early deep learning–based rainfall prediction, demonstrating DL’s ability to model nonlinear rainfall patterns
Zeyaeyan et al. (2017)	Rainfall warning classification using TOPSIS.	Meteorological severity indicators.	Iran Region.	Ranking-based only, not predictive.	Proposed a TOPSIS-based framework for ranking rainfall warning levels using meteorological severity indicators.
Nabeel and Athar (2018)	Wet/dry spell precipitation regime classification.	1980–2016 rainfall climatology.	Pakistan (National).	Descriptive classification only; no ML/DL forecasting.	Developed a national-scale classification of wet and dry precipitation regimes across Pakistan using long-term climatological data.
Oreshkin et al. (2019) – N-BEATS	Introduced a deep residual fully connected architecture using basis expansion blocks for interpretable time-series forecasting	M3, M4, and Tourism forecasting benchmark datasets	Global benchmark datasets	Requires careful architecture tuning and high computational cost for deep stacks; lacks integrated hyperparameter optimization strategies for convergence stability; limited support for automated feature engineering or signal decomposition; interpretability depends on predefined basis components and was evaluated mainly on benchmark forecasting datasets rather than domain-specific environmental applications.	Achieved state-of-the-art forecasting accuracy by introducing a deep residual fully connected architecture based on backward and forward basis expansion blocks, enabling explicit modeling of trend and seasonality components while improving interpretability and scalability for long-horizon time-series forecasting across benchmark datasets.
Iqbal et al. (2020)	Evaluation of precipitation projections using GCMs.	CMIP5/CMIP6 models.	Sub-Himalaya, Pakistan.	Climate projection only; no ML/DL event forecasting.	Evaluated CMIP5/CMIP6 climate models for precipitation projection in the sub-Himalayan region of Pakistan.
Arshad et al. (2021)	Validation of IMERG & TRMM precipitation products.	TRMM-3B42, IMERG.	Pakistan.	Validation-focused; no ML/DL forecasting pipeline.	Validated IMERG and TRMM satellite precipitation products for hydrological applications over Pakistan.
Wu et al. (2021) – Autoformer	Proposed a decomposition-based transformer architecture integrating progressive trend–seasonal decomposition and an Auto-Correlation attention mechanism for long-term time-series forecasting	Benchmark long-sequence datasets including Electricity, Traffic, Exchange Rate, Weather, and Influenza datasets	Global benchmark datasets	Large-scale structured datasets are necessary for reliable training, and design for continuous regression tasks rather than binary event categorization are susceptible to erratic weather signals and lack hybrid evolutionary optimization for better convergence and hyperparameter resilience.	Introduced progressive decomposition architecture embedded inside transformer layers and Auto-Correlation mechanism that captures periodic dependencies more efficiently than self-attention, achieving ≈ 38% relative improvement over prior models on six benchmark datasets
Azmi et al. (2021)	Naïve Bayes rainfall prediction classification	Meteorological features	Banyuwangi, Indonesia	Uses one model naïve Bayes only limited generalization	Demonstrated probabilistic rainfall classification using a Naïve Bayes model on regional meteorological data.
Rasool et al. (2023)	Rainfall-driven ML models for flood inundation mapping.	Rainfall + DEM + GIS.	Karachi.	Focus on flood mapping, not rain/no-rain prediction; no VMD/PSO.	• Integrated rainfall-driven ML models with GIS and DEM data for urban flood inundation mapping in Karachi.
Wani et al. (2023)	ML/DL rainfall forecasting across altitude.	Station + altitude climatology.	Himalayan region only.	Single climate zone, not generalizable across Pakistan.	Compared ML, DL, and time-series models across altitudinal gradients, highlighting elevation effects on rainfall prediction.
Praveena et al. (2023)	Logistic Regression + SVM rainfall prediction	Rainfall + meteorological features	India	Only LR/SVM; no DL; no VMD/PSO; limited multi-city validation	Applied classical statistical machine learning models, Logistic Regression (LR) and Support Vector Machine (SVM) to predict rainfall occurrence through meteorological variables, determined the baseline binary rainfall prediction performance without either changing model architecture or using deep learning
He et al. (2024)	Enhancing the precision of multi-step GRU-based monthly rainfall prediction.	Long-term rainfall time-series.	Pakistan (national).	Monthly scale only; no daily classification; no imbalance/VMD.	Applied GRU-based deep learning for national-scale monthly rainfall forecasting in Pakistan.
Zhou et al. (2024)	VMD-RNN used to enhance daily and hourly rainfall forecasting, a hybrid VMD-RNN model developed and was assessed using multifractal analysis.	POWER Project rainfall time series, daily and hourly datasets from Champs-sur-Marne	Champs-sur-Marne, France	-model not evaluated across diverse climates or multiple regions - Hourly performance weaker –Limited geographic scope -Does not incorporate distributed model inputs or regional rainfall variability.-The model has trouble overestimating and incorrectly classifying instances of light rainfall.	Introduced a hybrid VMD–RNN framework to enhance daily and hourly rainfall prediction via signal decomposition.
Farman et al. (2025)	Fusion ML + time-series rainfall prediction.	NASA POWER + PMD.	Pakistan multi-city.	Regression-based; no operational Rain/No-Rain classification.	Proposed a fusion of machine learning and time-series models for multi-city rainfall regression in Pakistan.
Farman et al. (2025b)	Activation function impact in ML/DL rainfall prediction.	Historical rainfall & climate features.	Pakistan (city-wise).	Focus on network optimization only; no VMD, no PSO, no multi-seed TSCV.	Analyzed the impact of activation functions on ML and DL rainfall prediction performance.
Ghosh et al. (2025) – ConvLSTM	Applied ConvLSTM with explainable AI for short-term precipitation prediction	Meteorological datasets from four Indian cities	India (four major cities)	Focused on short-term precipitation regression; limited geographic coverage and no multi-horizon rainfall occurrence classification framework or statistical cross-city validation	Demonstrated effectiveness of ConvLSTM in capturing spatio-temporal rainfall dynamics with explainability integration
Ria et al. (2026)	Developed a machine learning–based rainfall intensity classification framework using meteorological predictors such as humidity, sunshine duration, and temperature for operational rainfall warning decision support	Bangladesh Meteorological Department (BMD) station-based meteorological dataset with multi-class rainfall intensity categories	Multiple stations across Bangladesh (Mongla, Teknaf, Satkhira, Khulna)	Limited to single-step rainfall intensity classification; no multi-horizon forecasting; no deep sequential architectures (GRU/BiLSTM/Transformer); no signal decomposition (VMD); no evolutionary optimization (PSO/GA); lacks chronological TimeSeriesSplit validation	-Demonstrated effectiveness of classification-based rainfall prediction aligned with operational warning thresholds and identified humidity and sunshine duration as dominant predictors influencing rainfall occurrence probability
Risyad et al. (2026)	The study evaluated multiple classification algorithms to distinguish rainfall and non-rainfall events and assessed performance using standard classification metrics.	Local meteorological station dataset (daily rainfall observations and climate variables)	Indonesia (Palembang region)	Limited evaluation across multiple climatic zones; absence of deep learning sequence models; no multi-horizon rainfall forecasting; no signal decomposition techniques (e.g., VMD); no hyperparameter optimization using swarm intelligence; and no explainable AI integration for model interpretation.	Provided comparative evaluation of machine learning classifiers for rainfall occurrence prediction and demonstrated the feasibility of data-driven rainfall classification using structured meteorological predictors. Highest classification accuracy reported in the study ≈ **85%** (best-performing model).
**Proposed Study**					
Proposed study	-Proposed a VMD + PSO + GRU -framework for Rain/No-Rain classification -Proposed a multi-head VMD–GRU–Attention–PSO–GA hybrid deep learning framework for multi-horizon daily rainfall occurrence classification (RainDay0–RainDay5) using meteorological predictors across heterogeneous climatic regions of Pakistan.	NASA POWER meteorological dataset (multi-city daily climate observations)	10 climatic zones across Pakistan Karachi, Hyderabad, Thatta, Gharo, Nawabshah, Larkana, Quetta, Jamshoro, Hub (Lasbela), Mirpur Khas — Pakistan	-The framework focuses on binary rainfall occurrence classification (rain / no rain) of day0-day5 rather than quantitative rainfall intensity prediction, which may limit hydrological applicability for flood volume estimation. -Although the proposed hybrid VMD–GRU–Attention–PSO–GA framework improves predictive robustness and temporal feature extraction, the integration of decomposition-based preprocessing and evolutionary optimization increases computational requirements compared with baseline deep learning architectures, which may influence deployment efficiency in fully real-time streaming forecasting environments.	(i)Multi-horizon rainfall occurrence classification framework This study proposes a multi-horizon rainfall occurrence classification framework that integrates VMD-based signal decomposition, attention-enhanced GRU sequence learning, hybrid PSO–GA optimization, SHAP-based interpretability, and multi-seed TimeSeriesSplit validation for leakage-free temporal evaluation. The framework simultaneously predicts rainfall occurrence across short- (RainDay0–RainDay1), medium- (RainDay2–RainDay3), and extended lead-time horizons (RainDay4–RainDay5), demonstrating statistically significant improvements over baseline machine learning and deep learning models across heterogeneous climatic regions of Pakistan. (ii)Deployment-oriented Early Warning System (EWS) architecture -The study further proposes a deployment-oriented Early Warning System (EWS) architecture that translates probabilistic rainfall predictions into actionable decision-support outputs through threshold-based alert triggering, multi-level escalation warnings, dashboard visualization interfaces, and operational forecasting signals for disaster preparedness and climate-resilient urban planning. This architecture bridges the gap between experimental rainfall classification models and real-world early-warning deployment environments.

## Materials and methodology

**Fig. 2:**
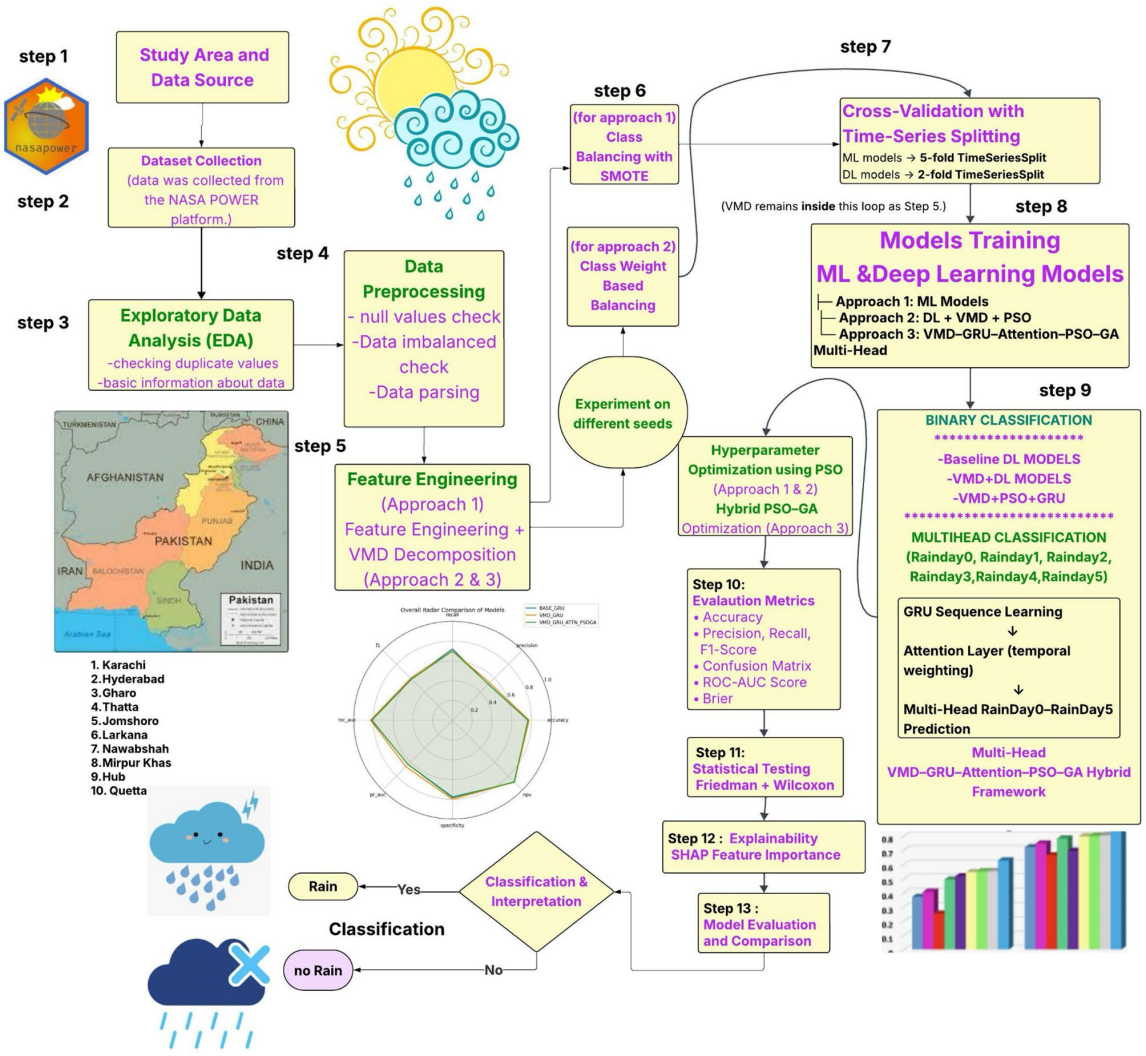
Overview of the proposed rainfall prediction framework showing three modeling pipelines: (i) ML models (RF, LR, GB, SVC) with PSO optimization for single-target classification, (ii) DL models (LSTM, BiLSTM, GRU, Transformer) with VMD and PSO tuning, and (iii) the proposed multi-head hybrid VMD–GRU–Attention–PSO–GA model for RainDay0–RainDay5 prediction.

The proposed experimental method illustrated in Fig. 2 relies on a comprehensive cross-comparative analysis of machine learning (ML) and deep learning (DL) models for predicting rainfall occurrence in ten climatically diverse cities in Pakistan. The framework consists of three complementary modeling approaches Approach 1 applies conventional machine learning classifiers with and without Particle Swarm Optimization (PSO) for single-target rainfall occurrence classification (rain vs. no rain). Approach 2 employs sequential deep learning architectures enhanced with Variational Mode Decomposition (VMD) and PSO-based hyperparameter optimization for single-target rainfall occurrence classification. Approach 3 introduces the proposed multi-head hybrid VMD–GRU–Attention–PSO–GA framework, which performs simultaneous multi-horizon rainfall occurrence prediction across forecast lead times RainDay0–RainDay5. Deep learning and machine learning connections were created with different representational goals in mind. While deep learning models were developed to take advantage of sequential temporal dependencies through sliding-window inputs, machine learning models were evaluated using static tabular meteorological predictors. Therefore, sequence learning, PSO-GA optimization, and VMD-based decomposition were applied in the deep learning framework to evaluate their combined effects in a phased ablation setting. The incremental effects of VMD, attention, and optimization are therefore examined through controlled comparisons within the same track among BASE-GRU, VMD–GRU, and VMD–GRU–Attention–PSO–GA, whereas ML-and-DL comparisons are viewed as cross-paradigm evaluations. This progressive experimental design allows systematic comparison between baseline tabular learning methods, sequential temporal modeling architectures, and the proposed hybrid multi-horizon optimization framework across heterogeneous climatic regimes of Pakistan. To ensure methodological consistency across model families and clarify differences in validation folds, seed settings, prediction types, and sequence window configurations, Table 2 summarizes the definitive experimental protocol used for all reported results.

**Proposed Methodology Approach 1:** Conventional machine learning classifiers with and without Particle Swarm Optimization (PSO) for single-target rainfall occurrence classification (rain vs. no rain).

**Table 2:** Definitive experimental protocol for rainfall occurrence classification models.

Approach	Model family	Prediction type	TimeSeries split	Seeds	lookback window	Feature set	Optimization	Selection criterion
Approach-1	ML (RF, LR, GB, SVC)	Binary classification (Rain / No Rain)	5 folds	42, 100, 123	Tabular (no sequence window)	Meteorological predictors + lag & rolling features	With / Without PSO	Validation Accuracy + F1-score
Approach-2	Baseline DL (GRU, LSTM, BiLSTM, Transformer)	Binary classification (Rain / No Rain)	2 folds	42, 100	7 days	Sequential meteorological predictors	None	Validation Accuracy
Approach-2	VMD–DL	Binary classification (Rain / No Rain)	2 folds	42, 3226	3, 5, 7, 14 days	Meteorological + VMD IMFs	PSO	ROC–AUC + F1-score
Approach-2	VMD–DL–PSO	Binary classification (Rain / No Rain)	2 folds	42, 3226	3, 5, 7, 14 days	Meteorological + VMD IMFs	PSO tuning	PR–AUC + Recall
Approach-3	Proposed VMD–GRU–Attention–PSO–GA Hybrid	Multi-horizon classification (RainDay0–RainDay5)	2 folds	42	7 days	Meteorological + VMD IMFs	Hybrid PSO–GA	PR–AUC + multi-horizon stability

The proposed study applies a machine learning (ML) model to predict the presence or absence of rainfall on a given day (Yes/No) using ten Pakistani cities, including Karachi, Hyderabad, Quetta, Thatta, Mirpur Khas, Larkana, Nawabshah, Gharo, Jamshoro, and Hub Lasbela (Balochistan), meteorological data from 1990 to 2025 (130,000 daily samples, 21 features). Such features are temperature, pressure, humidity, wind, and precipitation index. Mean-based imputation of missing values, feature standardization, and the Synthetic Minority Oversampling Technique (SMOTE) were used as data preprocessing measures to deal with class imbalance (rainfall: 15–30%). Only training folds were subjected to SMOTE so that there was no leakage into validation data. such as imbalance in Gharo Fold 1, {0: 1215, 1: 271}, was reduced to {0: 1215, 1: 1215}. TimeSeriesSplit was used to maintain temporal order. Each city was evaluated using five folds × three random seeds (42, 100, and 123) to ensure robustness and reproducibility. Pre-balancing sample sizes ranged from 1,400 to 7,400; after SMOTE, they ranged from 2,400 to 11,500; the validation set remained fixed at 1,486 samples per fold.

To capture both nonlinear relationships and linear decision boundaries in predicting rainfall occurrence, various machine learning classifiers were employed, including Histogram Gradient Boosting (HGB), Extra Trees (ET), Gaussian Naïve Bayes (GNB), and Ridge Classifier (RC). Both baseline and hyperparameter tuning configurations based on Particle Swarm Optimization (PSO) were utilized to train and evaluate these models. Accuracy, precision, recall, F1-score, ROC-AUC, and confusion matrices were computed separately for each city, seed, and validation fold to assess the model’s effectiveness. Early studies utilizing different TimeSeriesSplit configurations (3–5 folds) demonstrated the framework’s durability despite minor variations in fold structure, showing that model rankings remained stable with only slight differences in overall accuracy (under 1.5%). Table 2 summarizes a uniform experimental strategy to guarantee methodological consistency across model families and elucidate variations in validation folds, seed settings, prediction kinds, and sequence window configurations. The best model configurations were found using the combined data from all cities, mostly based on validation accuracy and F1-score.

**Proposed Methodology Approach 2:** (Sequential deep learning frameworks improved with Variational Mode Decomposition (VMD) and PSO-driven hyperparameter tuning for single-target rainfall event classification).

The study employed a deep learning (DL) framework to predict the occurrence (Yes/No) of rainfall using daily weather data from ten major Pakistani cities: Karachi, Hyderabad, Quetta, Thatta, Mirpur Khas, Larkana, Nawabshah, Gharo, Jamshoro, and Hub Lasbela (130,230 observations, 1990–2025).Because of its efficiency in processing sequential data, lower parameter complexity compared to LSTM, and capacity to forecast both short-term and long-term temporal relationships in climate time series, the Gated Recurrent Unit (GRU) architecture was selected as the primary one within the framework of the DL paradigm. The Gated Recurrent Unit (GRU) architecture was chosen as the main one within the framework of the DL paradigm due to its effectiveness in processing sequential information, lower complexity of parameters in comparison to LSTM, and the ability to predict both short-term and long-term temporal relationships in climate time series.

Preprocessing was also precise planned to prepare the data to be used in the temporal modeling. This was done by imputing missing values with mean substitution that was fitted only on training folds to ensure no information was leaked into validation sets. z-score scaling standardized the ranges of the features so that gradient propagation in optimization would remain constant. In order to harness the effect of sequential dependencies, the data set was converted into overlapping sliding windows. The GRU network was able to immediately learn temporal correlations across daily sequences because to the generation of three-dimensional input tensors (samples, time steps, and characteristics).

To evaluate model performance, a chronological walk-forward two-fold TimeSeriesSplit (TSCV) strategy was applied for each city to preserve temporal ordering. Experiments were conducted using random seeds (42 and 100) for baseline sequential deep learning models (GRU, LSTM, BiLSTM, Transformer), while seeds (42 and 3226) were used for VMD-enhanced architectures. A fixed 7-day sliding window was used for baseline sequential models, whereas multiple window lengths (3, 5, 7, and 14 days) were evaluated for VMD–DL and VMD–DL–PSO models to analyze the effect of temporal memory depth on rainfall occurrence classification performance as shown in Table 2.

The deep model network structure entailed a recurrent layer with 64 hidden units, and this layer was intended to be able to represent the time dynamics, and the network was followed by a dropout layer (rate = 0.3) to eliminate overfitting. The paradigm transformation of intermediate features was done by the assistance of a dense layer containing 32 neurons with ReLU activation and the final prediction layer by the assistance of a sigmoid activation, which produces binary rainfall predictions. It was trained on the Adam optimizer and binary cross-entropy loss and a learning rate of 0.001 over 30 epochs with mini-batches of 256. Early stopping was employed to avoid overfitting, and a patience of 3 epochs was employed to check the validation AUC.

In contrast to the ML framework, where SMOTE was used, sequence data needed a different solution to the issue of class imbalance. In this case, the dynamic class weights have been calculated per fold, based on the negative proportion of the class frequencies. This also made the minority rainy days have an equal say in the process of training but did not distort the time sequence of sequences. As a case study, in Gharo Fold 1, the validation data on rainy days was only 25.7%; class weighted the optimization procedure to make the rainy and dry cases contribute equal weight to the loss minimization.

The results of the Model were determined by Accuracy, Precision, Recall, F1 -score, and ROC-AUC which render the results similar to those of the ML results. In the case of class imbalance, Accuracy provided the global accuracy, Precision and Recall provided the trade-off between the false positives and false negatives and F1-score provided a combination of both such tradeoffs. ROC-AUC also assessed the discriminatory ability of the GRU against the thresholds and confusion matrices and curves of the ROC versus city-by-city to show the predictive reliability.

To select the model, the GRU configuration with the highest validation Accuracy across fold–seed combinations was identified per city, with F1-score and ROC-AUC used as tie-breakers. This provided even distribution of classification and strong discrimination in various rainfall regimes. The final DL workflow, alongside the ML pipeline, is summarized in Fig. 2.

**Fig. 3:**
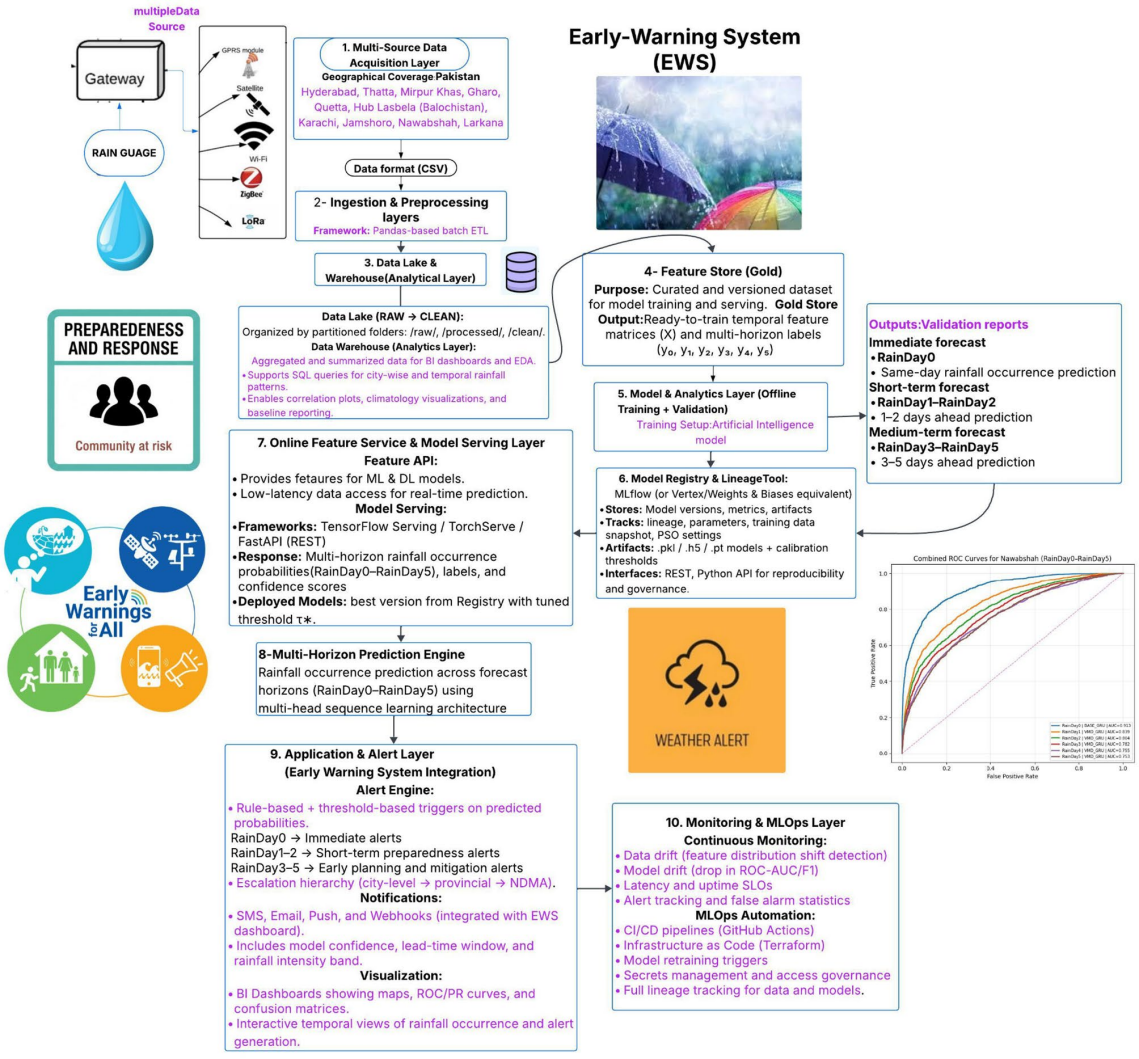
Study proposed framework for multihorizon rainfall occurrence early warning system.

The proposed end-to-end process of the early warning system (EWS) and rainfall occurrence prediction is presented in Fig. 3. This architecture integrates real-time alerting, machine and deep learning models, advanced feature engineering, and meteorological information obtained through multiple sources in one system. Out of raw data collection to implementation and surveillance of smart models, every step of the workflow has its own unique role in supporting the transformation of climate data into valuable information to be used in disaster planning and decision-making.

**Proposed Methodology Approach 3: (**the proposed multi-head hybrid VMD–GRU–Attention–PSO–GA framework, which performs simultaneous multi-horizon rainfall occurrence prediction across forecast lead times RainDay0–RainDay5)

Variational Mode Decomposition (VMD), GRU-based sequence modeling, an attention mechanism, and hybrid Particle Swarm Optimization–Genetic Algorithm (PSO–GA) hyperparameter optimization were all integrated into a single architecture for simultaneous multi-horizon prediction across forecast lead times RainDay0–RainDay5 in the third experimental track to create a hybrid multi-head rainfall occurrence prediction framework. VMD decomposes meteorological predictor signals into multiple frequency components representing different temporal variability scales. These IMFs were then added to the original feature space to improve representation of nonlinear rainfall dynamics without causing future-information leakage.

The dataset was converted into overlapping seven-day sliding windows to capture sequential climatic dependencies, resulting in three-dimensional tensors (samples, time steps, features). This made it possible for the GRU encoder to simultaneously learn patterns of rainfall persistence over several forecast horizons. The suggested architecture performs multi-head rainfall occurrence prediction, producing parallel outputs for RainDay0 through RainDay5 within a single network, in contrast to the single-target classification frameworks utilized in Approaches 1 and 2.

In order to better discriminate minority rainfall occurrences and improve the interpretability of temporal feature contributions across prediction horizons, an attention pooling method was included after the GRU encoder to adaptively weight informative temporal steps. Using dynamically calculated class-weighted binary cross-entropy loss obtained from training-fold label distributions, class imbalance was corrected.

A hybrid PSO–GA optimization technique was used to concurrently optimize GRU hidden units, dense-layer size, dropout rate, learning rate, and VMD parameters (K, α) in order to further enhance convergence stability and hyperparameter selection reliability. In this hybrid technique, GA adds crossover and mutation operators to boost diversity and avoid premature convergence, while PSO conducts global exploration of the hyperparameter search space.

To ensure consistency across many climatic regimes, the model was evaluated using chronological two-fold TimeSeriesSplit with seed 42. Accuracy, Precision, Recall, F1-score, ROC-AUC, PR-AUC, Brier score, false-alarm rate, and missed-event diagnostics were used to evaluate performance over all forecast horizons. The main methodological contribution of this study is the suggested VMD–GRU–Attention–PSO–GA multi-head architecture, which showed the highest stable and consistent rainfall occurrence prediction performance across all examined configurations.

## Proposed architecture of study

The suggested rainfall prediction and early warning workflow in Fig. 3 comprises nine functional layers that combine raw meteorological information and transform it into actionable information using a structured backend architecture. This workflow is initiated by the multi-source data acquisition layer that constantly receives rainfall, temperature, humidity, wind, and pressure information via rain gauges and model feeds across the major Pakistani cities (Karachi, Hyderabad, Quetta, and Thatta). These streams are transmitted through IoT gateways (GPRS, satellite, LoRa) into the backend.

The ingestion and preprocessing layer serves as the data-cleaning backbone and analyzes CSV streams with ETL scripts written in Pandas, parsing and imputing missing values and aligning timestamps, then pushes clean data to storage. The data lake tier serves as the main storage of the raw and cleaned data (RAW→CLEAN), whereas the data warehouse serves as the source of structured analytics to visualize, perform trend analysis, and explore statistics using the SQL queries.

The feature store (Gold tier) subsequently converts curated datasets into model-ready inputs, which include temporal features, lags, rolling statistics, and VMD-based Intrinsic Mode Functions (IMFs) by city to provide both short-term variability and long-term rainfall behavior.

The model and analytics layer is the computational core, where it trains different machine learning models (LR, KNN, DT, RF, GB), as well as deep learning models (LSTM, BiLSTM, GRU) with time-series cross-validation.In addition, the proposed hybrid VMD–GRU–Attention–PSO–GA architecture performs multi-horizon rainfall occurrence prediction across RainDay0–RainDay5, enabling simultaneous immediate, short-term, and medium-term early-warning forecasting within a unified multi-head sequence-learning framework.

In this framework, model hyperparameters are optimized using Particle Swarm Optimization (PSO) and hybrid PSO–GA strategies to improve convergence stability and parameter selection across cities and forecast horizons, while SMOTE is applied to balance class distributions for machine learning models. In addition, an attention mechanism is incorporated within the GRU-based architecture to emphasize informative temporal dependencies across multiple prediction horizons. The model registry (MLflow) stores trained models, performance metrics, configurations, and lineage information to ensure experiment traceability and reproducibility.

Using REST/gRPC services implemented through TensorFlow Serving or TorchServe, the online feature API and model serving layer provides low-latency prediction endpoints that generate multi-horizon rainfall occurrence probabilities, class labels, and confidence scores across forecast lead times (RainDay0–RainDay5) while dynamically updating lagged and rolling temporal predictors. The application and alert layer uses threshold-based triggering mechanisms and escalation logic to transform these outputs into actionable early warnings. Immediate alerts correspond to RainDay0 forecasts, short-term preparedness alerts correspond to RainDay1–RainDay2 predictions, and medium-term planning alerts correspond to RainDay3–RainDay5 forecasts, which are disseminated through dashboards, email notifications, and SMS-based communication channels integrated within operational Early-Warning System (EWS) platforms.

Finally, the monitoring and MLOps layer continuously checks for model and data drift, does automatic retraining using CI/CD pipelines, and manages security, lineage, and performance. A flexible and dynamic backend system that can support resilient rainfall predictions in a dynamic climatic environment is created by combining all of the custom-made layers: acquisition provides data integrity, preprocessing provides quality, the feature store enhances signals, modeling provides predictive insight, and serving provides insights to life-saving alerts.

It is important to note that the proposed Early-Warning System (EWS) architecture represents a deployment-oriented conceptual framework illustrating how the experimentally validated rainfall occurrence classification models developed in this study can be integrated into operational forecasting environments. The preprocessing pipeline of the data, featuring engineering, VMD-based signal decomposition, PSOGA hyperparameter optimization, TimeSeriesSplit-based temporal validation plan, and multi-horizon rainfall occurrence prediction models were implemented and tested using meteorological history. In contrast, infrastructure components such as IoT-based streaming ingestion, REST/gRPC model-serving interfaces, automated CI/CD retraining pipelines, and alert dissemination modules are presented as scalable system-level extensions intended for future operational deployment. The main control factor of the latency of operations in the proposed structure is the frequency of data reception in meteorological depositories, like the NASA POWER database; hence, the system is set up to work in the mode of near-real-time batch forecasting but not continuous streaming implementation. The system is to facilitate stable forecasting updates under meteorological data accessibility and communication latency limitations across the monitoring stations.

### Dataset description

The data employed in the given research is 35 years long (1990–2025) and includes ten large cities and towns of Pakistan that reflect the different climatic conditions, including both coastal and deltaic areas such as Karachi, Gharo, Thatta, Mirpur Khas; semi-arid interiors such as Hyderabad, Jamshoro, Nawabshah, and Larkana; and arid highlands such as (Quetta, Hub Lasbela, and Balochistan). This wide coverage is critical in ensuring that the models are tested in different rainfall regimes due to the variation in the maritime effects, monsoon, and extremes of the continent. The dataset contains 130,230 daily observations (or 1.3 lakh records) and 21 meteorological variables that have been obtained mostly through NASA POWER and GPM-IMERG products, which are augmented with earth-based calibration. Each city contributes around 13,000 daily records on average, which is large enough to train machine learning and deep learning models effectively.

The meteorological predictors were obtained from the NASA POWER project (https://power.larc.nasa.gov/) and GPM-IMERG precipitation products (https://gpm.nasa.gov/data/imerg). The complete implementation pipeline for the proposed multi-horizon rainfall occurrence classification framework is publicly available at the following repository: https://github.com/hira-farman/code-lac. Table 3 presents the general characteristics of the dataset used in this study.

**Table 3:** Summary of dataset characteristics.

Attributes	Description
No of observations	130,230
No of features	21
years	1990 to 2025 (3 full decades)
Country	Pakistan
City and town	Hyderabad, Thatta, Mirpur Khas, Gharo, Quetta, Hub Lasbela (Balochistan), Karachi, Jamshoro, Nawabshah, Larkana
Count of locations	10

The dataset includes a wide range of meteorological and time-related aspects, which reflects the multidimensional processes involved in the formation of rainfalls in the various climatic regions of Pakistan. These are spatial identifiers and time variables used in encoding seasonal and annual variability, solar radiation indicators like UV index and direct normal irradiance which controls surface heating. Variables associated with temperature such as mean, maximum, minimum, dew point, wet-bulb temperature and diurnal temperature range give information on the thermodynamics and moisture availability within the boundary-layer. Humidity parameters also describe the level of atmospheric saturation and the precipitation parameters provide a combination of ground-corrected rainfall and satellite-derived estimates, which indicates the observed and remotely sensed rainfall cues. The attributes of wind such as direction, speed at various heights, render mesoscale circulations which impact the formation of convective. These twenty-one predictors provide a physically significant and information-rich feature space, which allows the ML-DL system to model rainfall occurrence with a more sensitive perspective on local and regional atmospheric dynamics.

**Fig. 4:**
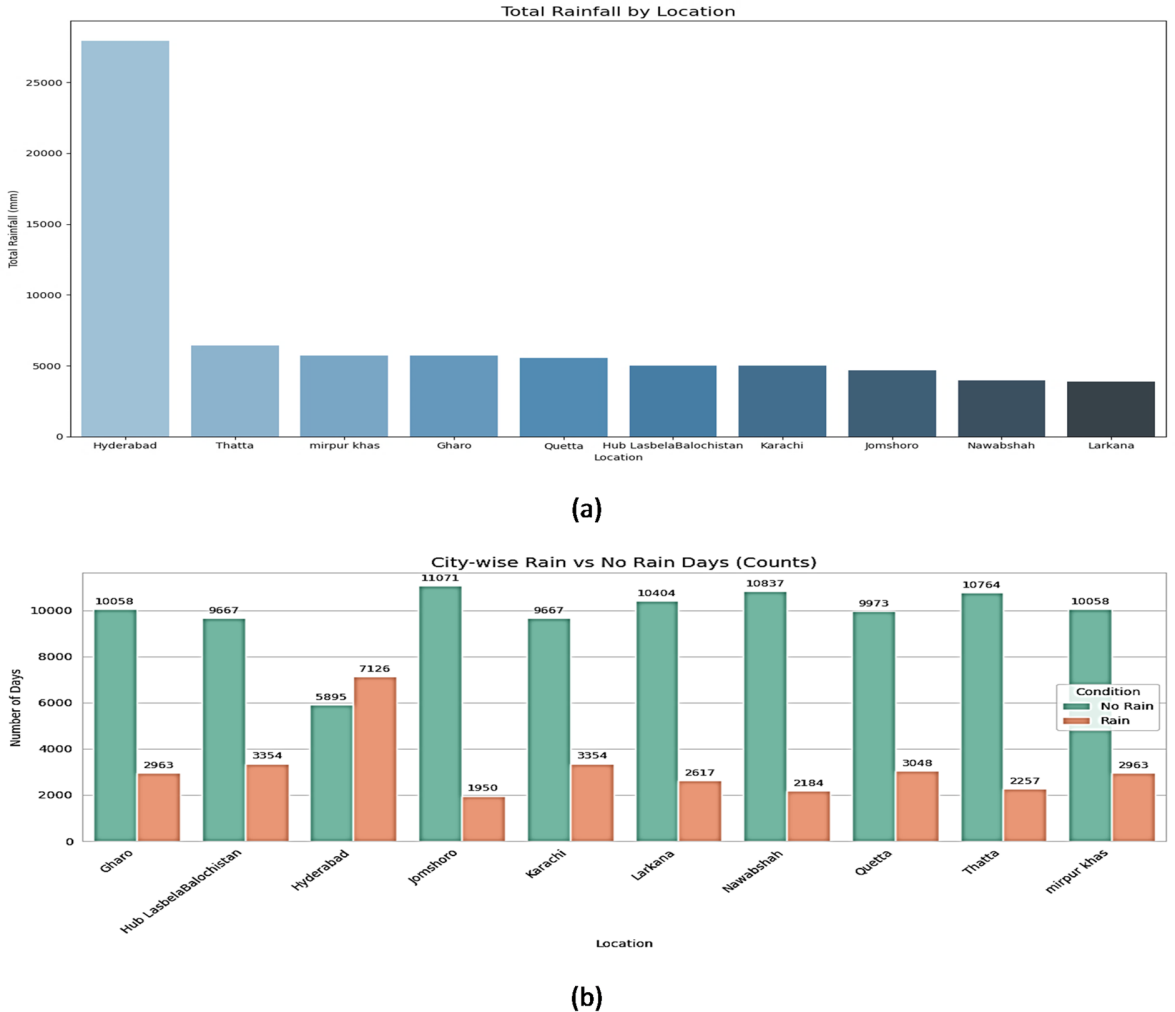
(**a**) Total rainfall distribution across cities (1990–2025). (**b**) City-wise distribution of rainy vs. non-rainy days (1990–2025).

Figure 4a shows the total rainfall distribution patterns of the ten chosen Pakistani cities within the study period (1990–2025). The total rainfall at Hyderabad is the highest then at Thatta, Mirpur Khas, and Gharo, meaning that the coastal areas and semi-humid areas are more affected by precipitation than the relatively drier areas like Nawabshah and Larkana. Such spatial differences indicate the climatic heterogeneity of the study region and the need to assess the suggested rainfall occurrence classification framework in the context of several regional rainfall regimes.

In Fig. 4b, this bar chart is used to compare the frequency of rainy and no-rainy days in ten cities in Pakistan. Hyderabad and Jamshoro have the largest ratio of rainy days and coastal areas like Karachi and Gharo have comparatively lower occurrences of rain. In general, all cities are dominated by non-rainy days and this indicates the existing imbalance of classes in the data. This asymmetry makes SMOTE applicable to machine learning models and class-weighted loss functions to deep learning architectures to make rainfall occurrence predictions in a city and forecast horizon unbiased across cities.

**Fig. 5:**
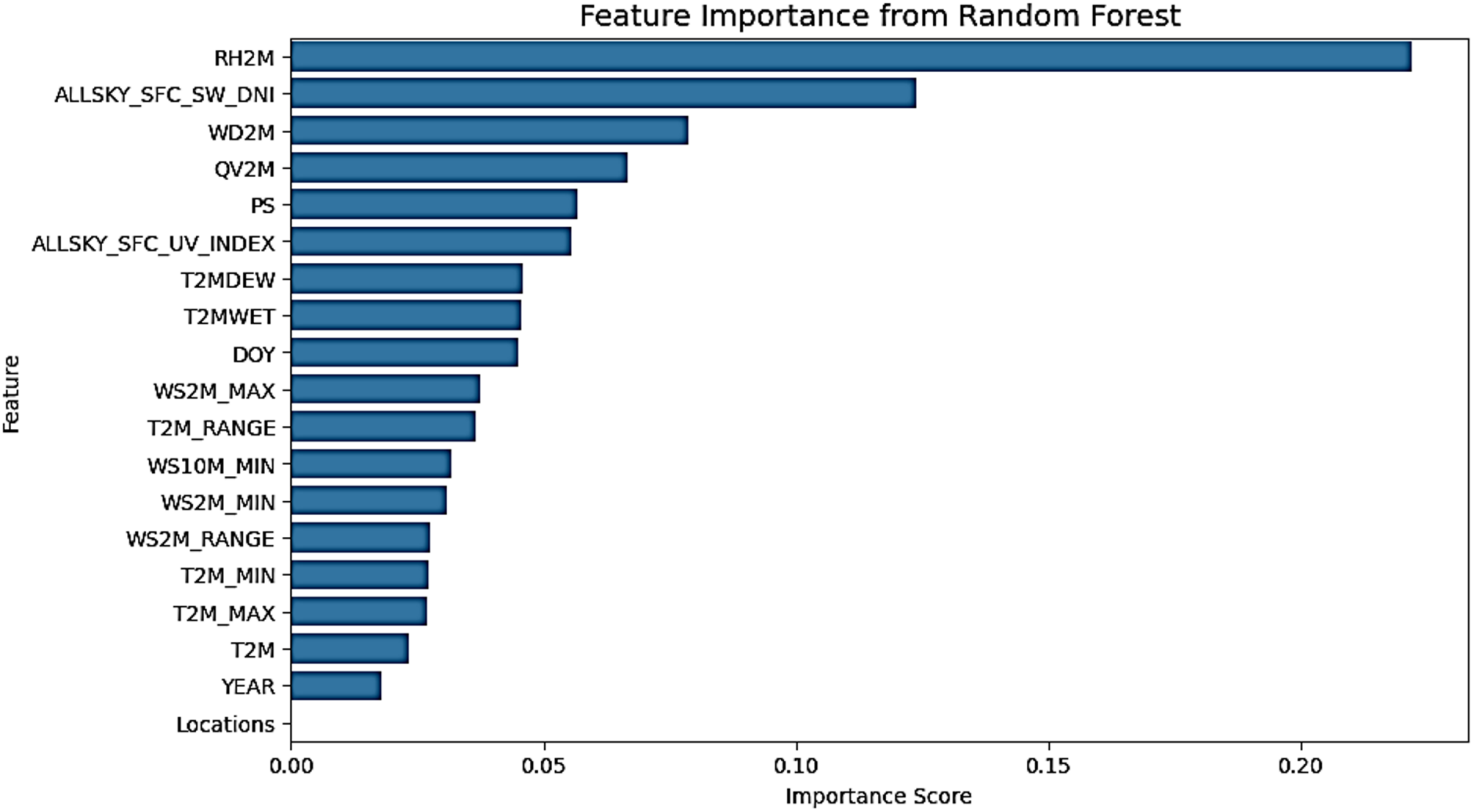
Feature importance from Random Forest classifier.

This bar plot in Fig. 5 ranks meteorological features by their contribution to rainfall prediction. Relative humidity at 2pm (RH2M) and surface irradiance variables (ALLSKY_SFC_SW_DNI, ALLSKY_SFC_UV_INDEX) emerged as the most influential predictors, followed by wind direction (WD2M), specific humidity (QV2M), and surface pressure (PS). Temperature variables (T2M, T2M_MAX, T2M_MIN) had lower predictive power. These findings emphasize the strong role in dynamic rainfall occurrence.

### Dataset overview and partitioning strategy

The dataset includes 21 meteorological indicators and 130,230 daily records from ten geographically diverse Pakistani towns from 1990 to 2025. To maintain predictive realism and avoid information leakage, all preprocessing methods, such as mean-value imputation and z-score normalization, were applied exclusively within training folds. All experiments utilized chronological TimeSeriesSplit validation to preserve the temporal sequence of observations. Dynamic class-weighted loss functions were employed to tackle class imbalance in sequential deep learning architectures, while SMOTE resampling was used for machine learning models. Table 2 summarizes the specific validation parameters, seed combinations, and sliding-window structures utilized in various modeling methods.

### Data preprocessing and quality control

By improving data dependability, lowering bias, and boosting generalizability across climatic regimes, the preprocessing offered robustness, fairness, and reproducibility. The data of each city was sorted by date to ensure temporal integrity, duplication was eliminated and missing data was filled in with mean substitution that was only fitted on training folds. Standardization to z-score was done to standardize features and unrealistic values were fixed to values that were physically realistic such as (negative rainfall, a humidity value exceeding 100%). The binary target variable (rain/no-rain) used was determined by a calibrated precipitation threshold. Class imbalance was effectively controlled at per-fold: ML pipelines used SMOTE on training folds, and DL models used dynamic class weighting. Other predictors that were less significant, including overlapping radiation or wind-range indices, were introduced into ML pipelines to maintain diversity but naturally de-emphasized in DL models by sequential learning. To ensure reproducibility, random seeds were fixed (42, 100, 123) and the same definition of folds was used in all experiments. Among the entire set of meteorological variables, three items were eliminated IMERG_PRECTOT, ALLSKY_SFC_SW_DNI, and ALLSKY_SFC_UV_INDEX because they had a high percentage of missing and low predictability as revealed through exploratory analysis. Training-fold statistics alone were used to scale the features and to impute missing values then applied to the validation folds to guarantee leakage-safe normalization.

### Time-series cross-validation (TSCV)

In comparison with the common k-fold cross-validation, TSCV relies on maintaining the time sequence of observations instead of a random separation of the data. In this approach the data is separated in sequential train-validation folds, where each training set is made up of data up to the validation window only. This avoids any leakage of information in the future to the past and the validation process of the future is similar to the real world forecasting conditions. Despite considering seasonal structures, monsoon cycles, multi-scale rainfall patterns, and temporal dependencies that are all essential to effective hydrological forecasts TSCV is specifically helpful in predicting rainfall. To boost robustness and reproducibility, TSCV was run on several folds per city and on multiple seeds (42, 100, 123) in this study. This type of design reduces chances of chance-driven results and is more efficient in capturing regional time scales of the rainfall^8^.

### Leakage control and temporal validation protocol

**Fig. 6:**
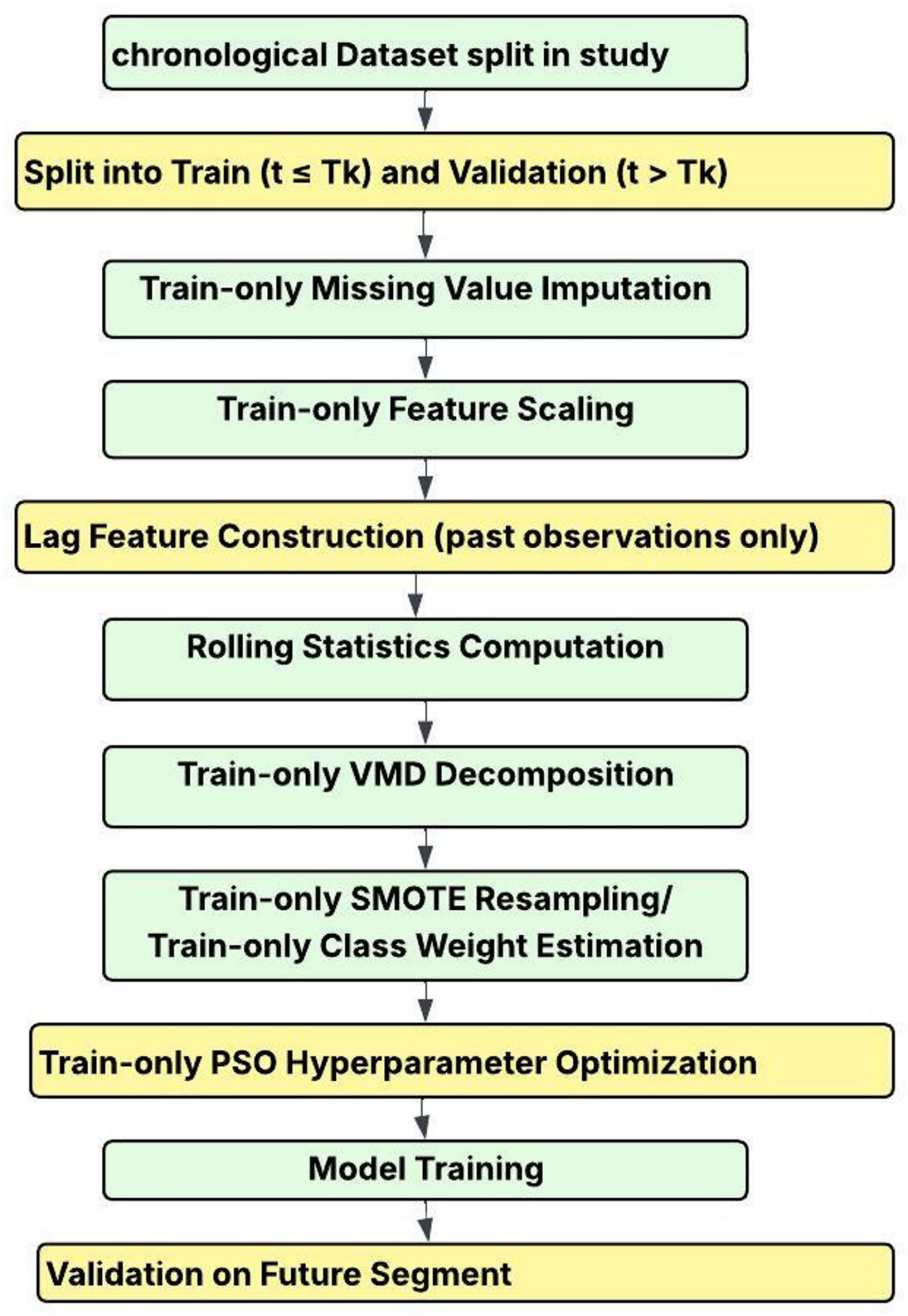
Fold-wise leakage-safe preprocessing workflow.

To ensure strict temporal validity and prevent information leakage between training and validation periods, all preprocessing and model optimization steps were performed within a fold-wise TimeSeriesSplit framework. Each fold preserved chronological ordering so that future observations were never used during model training. Missing-value imputation was performed using statistics computed from training folds only and then applied to validation folds. Feature scaling parameters were similarly estimated exclusively from training data and reused without recalibration on validation periods. Lagged predictors and rolling statistics were constructed using only historical observations preceding each prediction timestamp. Rainfall occurrence targets were thresholded after chronological partitioning to ensure that class-label generation did not incorporate information from future observations. Variational Mode Decomposition (VMD) was applied independently within each training fold to extract intrinsic mode functions representing rainfall signal components, and the learned decomposition parameters were subsequently used to transform validation segments without accessing future information. To address class imbalance, SMOTE resampling was applied strictly within training folds, while class weights for deep learning models were computed using training-label distributions only. Hyperparameter optimization using Particle Swarm Optimization (PSO) was performed within training folds using nested validation procedures, ensuring that validation-period information was never introduced during parameter selection. This fold-wise preprocessing protocol guarantees that the proposed rainfall classification framework remains fully leakage-safe under temporal cross-validation conditions. The fold-wise leakage-safe preprocessing workflow used in this study is illustrated in Fig. 6 and over all steps shown in Algorithm 1. Let the chronological dataset be defined as in Eq. (1).

1 $$\:\mathrm{D}=\:\left\{\:\right({x}_{t},{y}_{t}){\}}_{t=1}^{T},$$

where $\:{x}_{t}$∈ $\:{\mathfrak{R}}^{p}$ denotes the predictor vector at time t, and $\:{y}_{t}$∈{0,1} represents rainfall occurrence.

For each fold k in the TimeSeriesSplit framework, the dataset is partitioned as in Eq. (2a-2b).

2a $$\:{D}_{train}^{\left(k\right)}\:=\:\left\{\:\right(\:{x}_{t},\:{y}_{t})\:{\}}_{t=1}^{T},{D}_{val}^{\left(k\right)}\:=\:\{\:(\:{x}_{t},\:{y}_{t})\:{\}}_{t={T}_{k}+1}^{{T}_{k+1}},$$

such that

2b $$\:\mathrm{M}\mathrm{a}\mathrm{x}\:\left(\mathrm{t}\:\in {D}_{train}^{\left(k\right)}\right)\:<\:\:\mathrm{m}\mathrm{i}\mathrm{n}\:\left(\mathrm{t}\:\in {D}_{val}^{\left(k\right)}\right)$$

ensuring strict chronological separation between training and validation observations.

Feature scaling was performed using training-fold statistics only shown in Eq. (3).

3 $$\:{\underline{x}}_{t,j}=\frac{{x}_{i,j}-{\mu\:}_{j}^{\left(k\right)}\:}{{\sigma\:}_{j}^{\left(k\right)}}$$

where $\:{\mu\:}_{j}^{\left(k\right)}$ and $\:{\sigma\:}_{j}^{\left(k\right)}$ denote the mean and standard deviation computed from the training fold.

Lagged predictors were constructed causally as depicted in Eq. (4)

4 $$\:{x}_{t}^{\left(\mathpzc{l}\right)}={x}_{t-\mathpzc{l}},\mathpzc{l}\ge\:1,$$

ensuring dependence only on historical observations. Rolling statistics with window size w were computed as shown in Eq. (5).

5 $$\:\mathrm{R}\mathrm{o}\mathrm{l}\mathrm{l}\:\mathrm{M}\mathrm{e}\mathrm{a}\mathrm{n}\mathrm{t}=\frac{1}{w}{\sum\:}_{i=1}^{w}{x}_{t-i},$$

which prevents the use of current or future validation samples. Variational Mode Decomposition (VMD) was applied independently within each training fold depicted in Eq. (6):

6 $$\:{S}_{train}^{\left(k\right)}\left(\mathrm{t}\right)=\:{\sum\:}_{m=1}^{k}{u}_{m}^{\left(k\right)}\left(\mathrm{t}\right),$$

where intrinsic mode functions $\:{u}_{m}^{\left(k\right)}$(t) were estimated only from training observations and then applied to the validation segment without recomputing decomposition jointly across train and validation data. Synthetic minority samples generated using SMOTE were restricted to the training fold depicted in Eq. (7):

7 $$\:{x}_{new}={x}_{i}\:+{\uplambda\:}({x}_{i,NN}-{x}_{i}),\:\lambda\:\:\sim\:\:U\left(\mathrm{0,1}\right).$$

Class weights for deep learning models were computed from training labels only depicted in Eq. (8).

8 $$\:{w}_{c}^{\left(k\right)}=\:\frac{{N}^{\left(k\right)}}{2{N}_{c}^{\left(k\right)}}\:,\:\mathrm{c}\:\in\:\left\{\mathrm{0,1}\right\}$$

Hyperparameter optimization using Particle Swarm Optimization (PSO) was also restricted to training folds depicted in Eq. (9):

9 $$\:{\theta\:}_{i}^{(r+1)}={\theta\:}_{i}^{\left(r\right)}\:+\:{v}_{i}^{(r+1)},$$

ensuring that validation-period information was not used during parameter estimation.

Thus, all preprocessing transformations were learned exclusively from past observations, guaranteeing leakage-safe temporal validation across folds.

**Figure:**
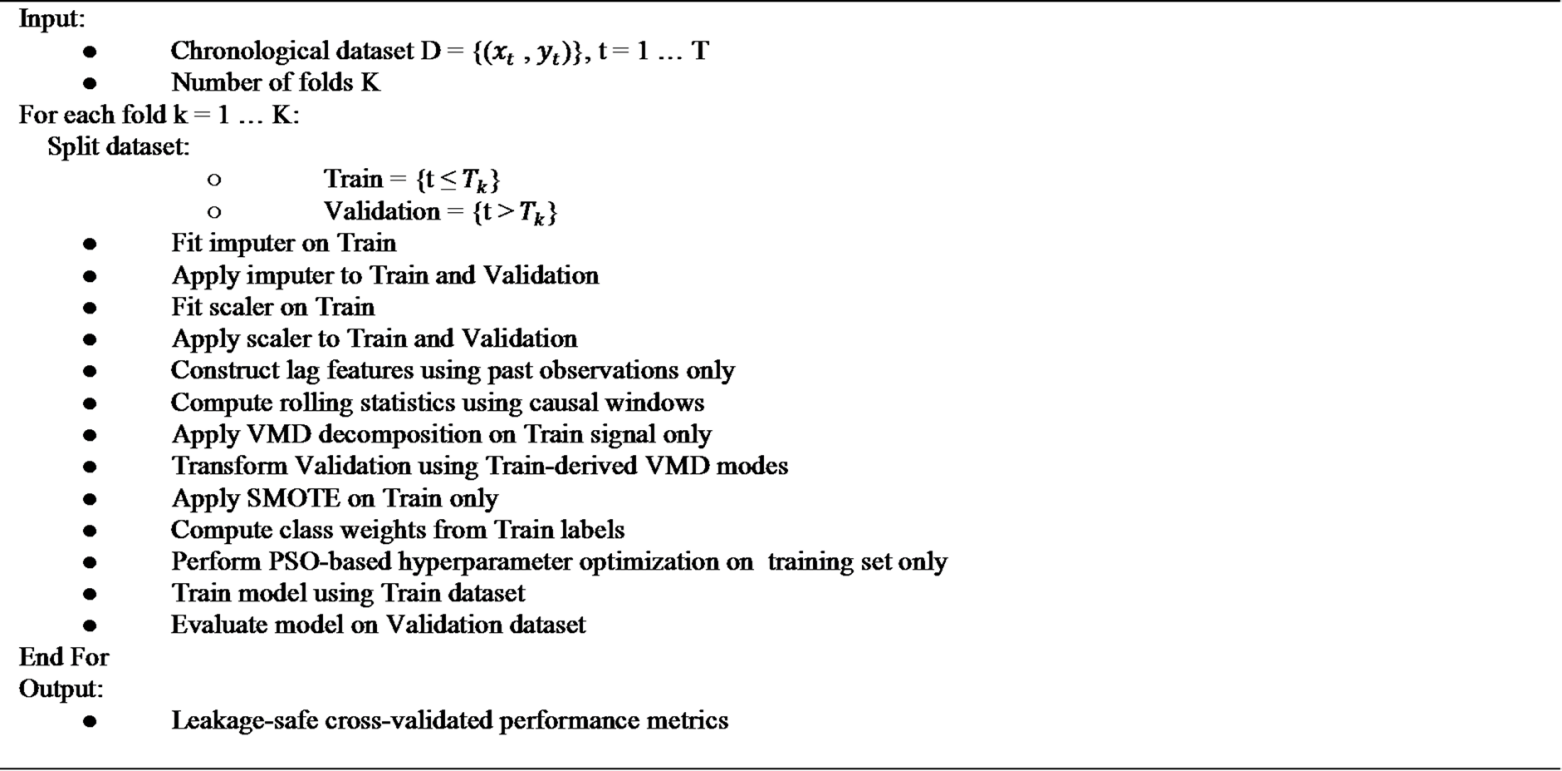
**Algorithm 1** Leakage-safe temporal training procedure.

### Feature engineering

To increase the effectiveness of model learning and lower input space noise, feature engineering entails the selection, transformation, and arrangement of predictor variables. In this study, physically interpretable meteorological predictors such as temperature, humidity, radiation, pressure, and wind-related variables were used to create both machine learning (ML) and deep learning (DL) pipelines. In order to minimize redundancy and maintain factors that are known to be relevant to the dynamics of rainfall occurrence, less informative aspects were eliminated. To address class imbalance for machine learning models without causing information leakage, feature standardization and SMOTE resampling were exclusively used during training folds. Additionally, without requiring significant human feature extension, tree-based classifiers took advantage of the natural nonlinear correlations between meteorological variables. Sliding-window input sequences built from core meteorological predictors were used to capture temporal dependencies for sequential deep learning models, and class imbalance was addressed using dynamically computed class-weighted loss functions rather than SMOTE to maintain sequence integrity.

Using Random Forest feature importance analysis (Fig. 5), the relative contribution of predictors was further investigated. This analysis revealed that variables related to humidity, radiation, wind direction, and pressure were the main contributors to rainfall occurrence classification performance.

#### Variational mode decomposition (VMD)

In approach 2 and 3 Prior to modeling, utilized in feature engineering Variational Mode Decomposition (VMD) to improve the quality and interpretability of rainfall signals. A complex time series can be broken down into a small number of band-limited Intrinsic Mode Functions (IMFs), each oscillating around a central frequency, using VMD, a fully non-recursive adaptive signal decomposition technique^14,28^. The separation of low-frequency components (long-term rainfall trends), medium-frequency oscillations (seasonal rainfall cycles), and high-frequency fluctuations (short-term rainfall variations, measurement noise) is made possible by this decomposition^6^.

VMD increases downstream prediction performance by producing a clearer feature representation by eliminating high-frequency noise and separating physically significant oscillations. This is the implementation strategy:

- VMD was applied only to deep learning models (LSTM, BiLSTM, GRU), as these architectures can directly exploit sequential dependencies within denoised IMFs.
- Traditional machine learning models (Logistic Regression, Random Forest, Gradient Boosting) were trained on engineered features without VMD, ensuring a fair comparison.
- Three IMFs (IMF1, IMF2, IMF3 ​) were extracted for each city and concatenated with temporal, cyclical, lagged, rolling, and categorical predictors to enrich the input space.
- To avoid data leakage, VMD was fitted only on training folds in the TimeSeriesSplit cross-validation scheme, and the learned decomposition was applied to the corresponding validation folds.

**Fig. 7:**
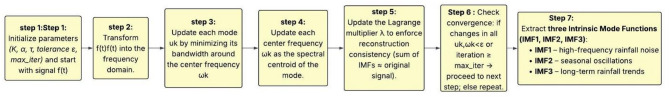
Variational mode decomposition (VMD) working flow.

The overall methodological pipeline was therefore and working flow VMD is depicted in Fig. 7:

Raw Data Preprocessing →Decomposition (VMD)→Optimization (PSO)→Model Training (DL) →Evaluation

The VMD framework decomposes a signal f(t) into a finite number of Intrinsic Mode Functions (IMFs) {$\:{{u}_{k}}_{k}$(t)$\:{\}}_{k=1}^{k}$ each centered around a frequency $\:{w}_{k}$. The variational problem is defined as in Eq. (10):

10 $$\:\underset{\left\{{u}_{k}\right\},\left\{{w}_{k}\right\}}{\mathrm{min}}{\sum\:}_{k=1}^{k}{\|{\partial\:}_{t}\left[\left(\delta\:\left(t\right)+\frac{j}{\pi\:t}\right)\:{*\:u}_{k}\left(t\right)\right]{e}^{-j{w}_{{k}^{t}}}\:\|}_{2}^{2}$$

subject to:

where:

- $\:{u}_{k}\left(t\right)$ = the k-th IMF,
- $\:{w}_{k}$ = the center frequency of $\:{u}_{k}$ ,
- δ(t) = Dirac delta function,
- ∗= convolution operator,
- j=$$\:\sqrt{-1}$$,
- f(t) = original rainfall signal.

In this work, the daily rainfall signal f(t) for each city was decomposed into K = 3, IMFs in Eq. (11):

11 $$f(t)\approx u_{1}(t)+u_{2}(t) +u_{3}(t)$$

- IMF1: captures high-frequency fluctuations (short-term rainfall variations, noise).
- IMF2: captures medium-frequency oscillations (seasonal rainfall cycles).
- IMF3: captures low-frequency components (long-term rainfall trends).

The Variational Mode Decomposition (VMD) parameters were configured as follows: time-step τ = 0, convergence tolerance 1 × 10^-7^, quadratic penalty term α = 2000, and number of modes K = 3. The penalty parameter α was determined by previous literature and initial experiments, which gave consistent mode separation with no over-smoothing. Because intrinsic mode functions are a key parameter controlling the trade-off between interpretability of the signal and over-decomposition, a sensitivity analysis was performed by changing the number of modes between K = 2 and K = 6. The findings showed that IMF 1 contained most of the low-frequency rainfall variability (6 to 7% variance contribution), IMF 2 contained most of the medium-frequency transitory dynamics (6 to 7%), and IMF 3 contained most of the high-frequency short-term dynamics (4 to 6%). Extra IMFs added the same amount of variance and produced noise sensitive components which did not enhance the performance of the classification process. Thus, it was decided to use K = 3 as an optimal amount of decomposition to be used in predicting stable and computationally efficient multi-horizon rainfall occurrence across all cities. VMD decomposition was carried out in each training fold separately to avoid temporal leakage, and then applied to the training-period observations-only to the separate validation segment.

### Machine learning and deep learning model discussion employed in study for rainfall classification

Using the scikit-learn framework, machine learning models such as Histogram Gradient Boosting (HGB), Extra Trees (ET), Gaussian Naïve Bayes (GNB), and Ridge Classifier (RC) were developed in Approach-1 and assessed in both baseline and PSO-optimized configurations. While tree-based ensemble models (HGB and ET) directly incorporated nonlinear correlations from meteorological predictors without requiring significant feature modification, the Ridge Classifier used standardized feature inputs with L2 regularization. In approach 2% 3 several deep learning models evaluated such as CNN, LSTM, BISLTM, Transformer and GRU, the Gated Recurrent Unit architecture comprised a 64-unit recurrent layer, a dropout rate of 0.3, a dense layer with 32 ReLU units, and a sigmoid output. The network was trained with the Adam optimizer (learning rate is 1e-3), a mini-batch size of 256, binary cross-entropy loss, and early stopping based on validation AUC.

### Gradient boosting

Gradient Boosting builds an ensemble of trees sequentially, where each new tree is trained to correct the residual errors of the current model^29^. This yields high predictive power and adapts well to complex, nonstationary rainfall patterns, including extreme events^25^. The stage-wise additive model is shown in Eq. (12).

12 $$\:{F}_{m}\left(\mathrm{x}\right)={F}_{m}-1\left(\mathrm{x}\right)+{\upnu\:}\cdot\:\:{h}_{m}\left(\mathrm{x}\right)$$

where $\:{h}_{m}$( (x)is the weak learner (tree) at stage m, and ν is the learning rate.

### Extra trees classifier (ET)

The Extra Trees Classifier is an ensemble learning method that constructs multiple unpruned decision trees using the entire training dataset. Unlike Random Forest, ET selects cut-points for splits at random, which increases variance reduction while lowering computational cost by Rajab et al.^25^. The prediction is based on the majority vote of individual trees as mathematically depicted in Eq. (13):

13 $$\:\widehat{y}\:=\mathrm{m}\mathrm{o}\mathrm{d}\mathrm{e}\left\{{h}_{1}\:\right(\mathrm{x})\:,{h}_{2}\:(\mathrm{x}),\dots\:,{h}_{t}\:(\mathrm{x})$$

where $\:{h}_{t}$ (x) denotes the prediction from the $\:{t}^{th}$ tree and T is the total number of trees. By incorporating randomness in feature thresholds, the classifier provides robust predictions and reduces overfitting, making it effective for high-dimensional meteorological features.

### Histogram-based gradient boosting (HGB)

The Histogram-based Gradient Boosting Classifier accelerates traditional boosting by discretizing continuous features into histogram bins Hasany et al.^29^. This reduces computational cost while maintaining model accuracy^25^. The iterative model update is depicted in Eq. (14).

14 $$\:{F}_{m}\left(\mathrm{x}\right)={F}_{m-1}\left(\mathrm{x}\right)+{\upeta\:}\cdot\:{h}_{m}\left(\mathrm{x}\right)$$

where $\:{h}_{m}\left(x\right)$ is the weak learner fitted to the pseudo-residuals and η controls learning rate. HGB efficiently captured rainfall variability across large datasets by leveraging histogram-based feature partitioning.

### Gaussian Naïve Bayes (GNB)

Gaussian Naïve Bayes applies Bayes’ theorem under the assumption of conditional independence between features^42^. For rainfall classification, the posterior probability of class y given features $\:{x}_{1}$ ,$\:{x}_{2}$,…,$\:{x}_{n}$ ​ is shown in Eq. (15a):

15a $$\:\mathrm{P}\left(\mathrm{y}\right|{x}_{1}\:,{x}_{2},\dots\:,{x}_{n})\propto\:\mathrm{P}(\mathrm{y}){\prod\:}_{i=1}^{n}p\left(\right|{x}_{i}\:\left|y\right)$$

Assuming Gaussian-distributed features, the conditional likelihood as in Eq. (15b):

15b $$\:\mathrm{P}\left(\:{x}_{i}\right|\mathrm{y})=\:\frac{1}{\sqrt{2\varPi\:\:{\sigma\:}_{y}^{2}}}\:\mathrm{e}\mathrm{x}\mathrm{p}(\:-\frac{{\left({x}_{i\:-}\:{\mu\:}_{y}\right)}^{2}}{2\:{\sigma\:}_{y}^{2}})$$

where $\:{\mu\:}_{y}\:and\:{\sigma\:}_{y}^{2}$ are the mean and variance of feature $\:{x}_{i}$​ in class y. GNB is computationally efficient and effective in cases where features are approximately normally distributed, making it suitable for rainfall occurrence classification.

### Calibrated ridge classifier (RidgeCal)

Ridge Classifier extends linear classification by incorporating an L2​ penalty on the weight vector. The optimization problem is defined as in Eq. (16a).

16a $$\:{w}^{min}{\Vert\:Xw-y\Vert\:}^{2}\:+{\upalpha\:}\:{\Vert\:w\Vert\:}^{2}$$

where X is the input matrix, y the target labels, w the weight vector, and α the regularization parameter. The calibration step maps the decision scores into well-calibrated probabilities using Platt scaling (sigmoid method). The calibration process maps the uncalibrated model outputs f into probabilistic estimates as shown in Eq. (16b).

16b $$\:\mathrm{p}(\mathrm{y}=1\mid\:\mathrm{f})=\frac{1}{1+expexp\:(Af+B)\:}$$

where f is the raw model score, and A and B are calibration parameters learnt on a validation set. Ridge Cal provided both regularized linear decision boundaries and reliable probability estimates, which are critical for probabilistic rainfall forecasting.

### Deep learning models utilized in study for rainfall classification

#### Recurrent neural network (RNN)

Temporal dependencies are incorporated through recurrent connections in Recurrent Neural Networks (RNNs), which are built for sequential data. The current input and the prior hidden state are used to update the hidden state at each time step t Hernández et al.^22^ as shown in Eq. (17a).

17a $$\:{h}_{t}\:=\mathrm{t}\mathrm{a}\mathrm{n}\mathrm{h}({w}_{h}{h}_{t-1}\:+\:{w}_{x}{x}_{t}\:+\:{b}_{h}\:)$$

The final output is computed as in Eq. (17b):

17b $$\:\:{y}_{t}={\upsigma\:}({w}_{y}{h}_{t}+{b}_{y})$$

where $\:{x}_{t}$ is the input, $\:{h}_{t}$ the hidden state, W weight matrices, and σ the activation (e.g., sigmoid). Despite their simplicity, RNNs suffer from vanishing gradients when modeling long-term dependencies.

#### Long short-term memory (LSTM)

Recurrent neural networks (RNNs) have evolved into Long Short-Term Memory networks, which are specifically made to solve the vanishing gradient issue that ordinary RNNs face^32^. Three gate the forget, input, and output gates control the information flow in a memory cell introduced by the LSTM^3^ For sequential data, such as rainfall prediction, speech recognition, and natural language processing, LSTMs are very effective because of these gates, which allow the network to preserve long-term relationships while eliminating unnecessary information He et al.^38^. Equations (18–23) displays the governing equations of an LSTM cell:

18 $$\:\mathrm{F}\mathrm{o}\mathrm{r}\mathrm{g}\mathrm{e}\mathrm{t}\:\mathrm{G}\mathrm{a}\mathrm{t}\mathrm{e}:\:\mathrm{f}\left(\mathrm{t}\right)\:=\:\:{W}_{f}\:\ast\:\left[\mathrm{h}\right(\mathrm{t}-1),\:\mathrm{x}(\mathrm{t}\left)\right]\:+\:{b}_{f}$$

19 $$\:\mathrm{I}\mathrm{n}\mathrm{p}\mathrm{u}\mathrm{t}\:\mathrm{G}\mathrm{a}\mathrm{t}\mathrm{e}:\:\mathrm{f}\left(\mathrm{t}\right)\:=\:\:{W}_{f}\:\ast\:\left[\mathrm{h}\right(\mathrm{t}-1),\:\mathrm{x}(\mathrm{t}\left)\right]\:+\:{b}_{I}$$

20 $$\:\mathrm{C}\mathrm{a}\mathrm{n}\mathrm{d}\mathrm{i}\mathrm{d}\mathrm{a}\mathrm{t}\mathrm{e}\:\mathrm{C}\mathrm{e}\mathrm{l}\mathrm{l}\:\mathrm{S}\mathrm{t}\mathrm{a}\mathrm{t}\mathrm{e}:\:\underline{{C}_{t}}\:=\:\mathrm{t}\mathrm{a}\mathrm{n}\mathrm{h}\:{W}_{f}\:\ast\:\left[\mathrm{h}\right(\mathrm{t}-1),\:\mathrm{x}(\mathrm{t}\left)\right]\:+\:{b}_{c}$$

21 $$\:\mathrm{C}\mathrm{e}\mathrm{l}\mathrm{l}\:\mathrm{S}\mathrm{t}\mathrm{a}\mathrm{t}\mathrm{e}\:\mathrm{U}\mathrm{p}\mathrm{d}\mathrm{a}\mathrm{t}\mathrm{e}:{c}_{t}\:=\:{f}_{t}\odot\:\:{c}_{t-1}\:+{i}_{t}\odot\:\:\underline{{C}_{t}}$$

22 $$\:\mathrm{O}\mathrm{u}\mathrm{t}\mathrm{p}\mathrm{u}\mathrm{t}\:\mathrm{G}\mathrm{a}\mathrm{t}\mathrm{e}:\:{o}_{t\:}=\:\:{W}_{o}\:\ast\:\left[\mathrm{h}\right(\mathrm{t}-1),\:\mathrm{x}(\mathrm{t}\left)\right]+{b}_{o}$$

23 $$\:\mathrm{H}\mathrm{i}\mathrm{d}\mathrm{d}\mathrm{e}\mathrm{n}\:\mathrm{S}\mathrm{t}\mathrm{a}\mathrm{t}\mathrm{e}\:\mathrm{U}\mathrm{p}\mathrm{d}\mathrm{a}\mathrm{t}\mathrm{e}:{h}_{t\:}\:={o}_{t\:}\odot\:\:\mathrm{t}\mathrm{a}\mathrm{n}\mathrm{h}\left(\:{c}_{t}\:\right)$$

Here, $\:{\:x}_{t}$is the input at time step, t,$\:{h}_{t}$ is the hidden state $\:{c}_{t}$ denotes the sigmoid activation function and ⊙ represents element-wise multiplication.

#### Bidirectional LSTM

When small temporal windows are employed for rainfall classification, BiLSTM is particularly helpful because climatic cues that affect the occurrence of rainfall may exist both before and after a particular time step within the sequence. Bidirectional LSTM (BiLSTM) extends the standard LSTM by processing a sequence in two directions: one LSTM reads forward (past → future), and another reads backward (future → past) Waqas et al.^2^ At each time step t, their hidden states are concatenated to form a context that captures information from both past and future positions in the sequence, as shown in Eq. (27). This often improves sequence modelling compared to a single (unidirectional) LSTM, especially when the full sequence is available during training evaluation Wani et al.^30^

24 $$\:{h}_{t}^{BLSTM}\:=\:\:\overrightarrow{{h}_{t}}\:\:\parallel\:\:\:{h}_{t}^{\leftarrow}$$

#### Gated recurrent unit (GRU)

For large-scale climate datasets when quicker convergence and less parameter complexity are needed, GRU provides an advantageous trade-off between computing efficiency and temporal modeling capacity.The Gated Recurrent Unit is a condensed version of LSTM that should reduce the computational cost without impacting the performance of the system in sequential tasks^2^. GRUs use a reset gate to control the level of the forgotten historical information and incorporate the input and forget gates into one update gate^3^. This renders GRUs especially appropriate to medium-sized rainfall datasets and long-range time prediction, and allows making the convergence faster and efficient training^40^. The following are the main Eqs. (25–28):

25 $$\:\mathrm{U}\mathrm{p}\mathrm{d}\mathrm{a}\mathrm{t}\mathrm{e}\:\mathrm{g}\mathrm{a}\mathrm{t}\mathrm{e}:\:{z}_{t}=\:({w}_{z}\:\cdot\:[{h}_{t-1}\:,\:{\:x}_{t}\:]\:+\:{b}_{z}\:)$$

26 $$\:\mathrm{R}\mathrm{e}\mathrm{s}\mathrm{e}\mathrm{t}\:\mathrm{g}\mathrm{a}\mathrm{t}\mathrm{e}:\:{r}_{t}=({w}_{r}\:\cdot\:[{h}_{t-1}\:,\:{\:x}_{t}\:]\:+\:{b}_{r})$$

27 $$\:\mathrm{C}\mathrm{a}\mathrm{n}\mathrm{d}\mathrm{i}\mathrm{d}\mathrm{a}\mathrm{t}\mathrm{e}\:\mathrm{a}\mathrm{c}\mathrm{t}\mathrm{i}\mathrm{v}\mathrm{a}\mathrm{t}\mathrm{i}\mathrm{o}\mathrm{n}:\stackrel{\sim}{{h}_{t}}=\mathrm{t}\mathrm{a}\mathrm{n}\mathrm{h}\:({w}_{h}\:\cdot\:[{r}_{t}{*\:h}_{t-1},{\:x}_{t}\:]\:+\:{b}_{h}\:)$$

28 $$\:\mathrm{F}\mathrm{i}\mathrm{n}\mathrm{a}\mathrm{l}\:\mathrm{h}\mathrm{i}\mathrm{d}\mathrm{d}\mathrm{e}\mathrm{n}\:\mathrm{s}\mathrm{t}\mathrm{a}\mathrm{t}\mathrm{e}:\:{h}_{t}=\:(1-\:{z}_{t}\:)\:\mathrm{*}\:{h}_{t-1}+{\:z}_{t}\:\mathrm{*}\stackrel{\sim}{{\:h}_{t}}$$

#### Bidirectional gated recurrent unit (BiGRU)

In order to capture both past and future historical context, a bidirectional GRU analyses the sequence in both temporal directions. Two GRU cells are stacked as a forward GRU that reads {$\:{X}_{1}$,…,$\:\:{X}_{T}$} and a reverse GRU that reads {$\:{X}_{T\:}$,…,$\:\:{X}_{1}$}^3^. Each side’s hidden states are concatenated at each time step in accordance with the usual GRU gating in Equations. (29a–31d).

Forward and backward recurrences and their concatenation shown in Eq. (29a and 29b):

29a $$\:\overrightarrow{{h}_{t}}{GRU}_{f}({X}_{t\:},\:\overrightarrow{{h}_{t-1}}\:)\:,{h}_{t}^{\leftarrow}{GRU}_{b}\:({X}_{t\:},\:{h}_{t}^{\leftarrow}-1)$$

Context fusion by concatenation:

29b $$\:{h}_{t}=[\:\overrightarrow{{h}_{t}}\:;\:{h}_{t}^{\leftarrow}]\:\in\:\:{R}^{2H}$$

Sequence labelling prediction (one output per time step) as in Eq. (30):

30 $$y_{t}=g(W_{o}h_{t}+b_{o})$$

Sequence classification prediction (one output for the entire window) shown in Eq. (31):

31 $$\:\mathrm{y}=\mathrm{g}\left({W}_{o}\right[\:\overrightarrow{{h}_{T\:\:}};\:{h}_{1}]\:+{b}_{o})$$

Here, g(⋅) is a task-appropriate activation such as (sigmoid for binary classification), HHH is the number of units per direction, and [⋅ ; ⋅]denotes vector concatenation. BiGRU has computational efficiency similar to a unidirectional GRU, but uses both future and past context, which can be useful in short meteorological windows, where the cues might exist in both directions of the sequence.

#### Convolutional neural network (CNN)

Convolutional Neural Networks (CNNs) are widely used in spatial and temporal feature extraction from gridded meteorological data or sequential time-series inputs^43^ In rainfall prediction, CNNs efficiently learn localized temporal and spatial correlations such as (between humidity, temperature, and wind speed) by applying convolutional filters over the input sequence. CNNs thus hierarchically capture rainfall-related dependencies from short-term variations learnt by shallow filters to seasonal or large-scale temporal dynamics encoded in deeper layers, making them highly suitable for spatiotemporal rainfall modeling and occurrence prediction. In order to extract spatial characteristics throughout the input domain, the convolutional layer performs nonlinear activation after computing weighted local sums of surrounding pixels, as seen in Eqs. (32a) and (32b). At each layer l, the convolutional operation for feature map k is given by:

32a $$\:{Z}_{i,j}^{\left(k\right)}=\sum\:_{m}\sum\:_{n}{x}_{i+m,j+n}.\:{W}_{m,n}^{\left(k\right)}+\:{b}^{\left(k\right)}$$

32b $$\:{a}_{i,j}^{\left(k\right)}=f\left({Z}_{i,j}^{\left(k\right)}\:\right)\:$$

where $\:{x}_{i,j}$ is the input value at position (i, j)$\:\:{W}_{m,n}^{\left(k\right)}\:$ is the convolutional kernel weight, $\:{b}^{\left(k\right)}$is the bias term, and f(⋅) is a nonlinear activation function such as(ReLU or tanh).

Pooling layers such as(max-pooling) further reduce dimensionality while preserving dominant features shown in Eq. (33):

33 $$\:{P}_{i,j}^{\left(k\right)}\:=\:{a}_{i+m\:,\:j+n}^{\left(k\right)}\:$$

where: R defines the pooling region.

The final output from flattened feature maps passes through fully connected layers and a sigmoid activation for binary rainfall classification shown in Eq. (34):

34 $$\:\widehat{y}={\upsigma\:}(\:{W}_{fc}\cdot\:\mathrm{p}+{b}_{fc})$$

where p is the flattened feature vector, $\:{W}_{fc}$ and $\:{b}_{fc}$ ​denote the weights and bias of the dense layer, and σ(⋅) is the sigmoid function.

#### Transformer model

Although the Transformer architecture only uses self-attention mechanisms instead of recurrence, it constitutes a paradigm shift in sequential modeling. This gives the model the ability to simultaneously incorporate global dependencies across all time steps, which is a major benefit for rainfall prediction in situations where there are long-range temporal linkages between past and future climatic conditions^10^. Multi-head self-attention and feed-forward networks comprise each Transformer encoder layer. The scaled dot-product attention was calculated. Equations (35)–(38) show how the Transformer models long-range meteorological dependencies like seasonal cycles, monsoon patterns, and delayed rainfall effects more efficiently than traditional recurrent architectures when processing long sequential data with global attention mechanisms.

35 $$\:\mathrm{A}\mathrm{t}\mathrm{t}\mathrm{e}\mathrm{n}\mathrm{t}\mathrm{i}\mathrm{o}\mathrm{n}(\mathrm{Q},\mathrm{K},\mathrm{V})=\mathrm{s}\mathrm{o}\mathrm{f}\mathrm{t}\mathrm{m}\mathrm{a}\mathrm{x}\left(\frac{Q{K}^{T}}{\sqrt{{d}_{k}}}\right)\mathrm{V}$$

where the query, key, and value matrices obtained from the input are denoted by Q, K, and V, and $\:{d}_{k}$ is the dimensionality of the key vectors used for normalization. For multi-head attention, the model employs multiple parallel attention mechanisms:

36 $$\:\mathrm{M}\mathrm{u}\mathrm{l}\mathrm{t}\mathrm{i}\mathrm{H}\mathrm{e}\mathrm{a}\mathrm{d}(\mathrm{Q},\mathrm{K},\mathrm{V})=\mathrm{C}\mathrm{o}\mathrm{n}\mathrm{c}\mathrm{a}\mathrm{t}({head}_{1},{head}_{h})$$

where:

$\:{head}_{i}=\mathrm{A}\mathrm{t}\mathrm{t}\mathrm{e}\mathrm{n}\mathrm{t}\mathrm{i}\mathrm{o}\mathrm{n}(\mathrm{Q}{W}_{i}^{Q},K{W}_{i}^{K},\mathrm{V}\:{W}_{i}^{V}$), Here $\:{W}_{i}^{Q}\:,{W}_{i}^{K}\:,\:{W}_{i}^{V}\:and\:\:{W}^{o}\:$are learnable projection matrices.

The output of the attention mechanism passes through a position-wise feed-forward network:

37 $$\:\mathrm{F}\mathrm{F}\mathrm{N}\left(\mathrm{x}\right)=\mathrm{R}\mathrm{e}\mathrm{L}\mathrm{U}(\mathrm{x}{W}_{1}\:+\:{b}_{1})$$

Since Transformers lack inherent sequence ordering, positional encodings are added to the input embedding’s: These encodings inject temporal order into the sequence. For rainfall classification, the final prediction is obtained via a sigmoid activation applied to the output embedding of the encoder:

38 $$\:\widehat{y}={\upsigma\:}(\:{W}_{o}.{h}_{enc}+\:{b}_{o})$$

The Transformer effectively models long-range meteorological dependencies such as seasonal cycles and delayed rainfall effects, outperforming recurrent architectures in handling long sequences with global attention mechanisms.

### Hyperparameters optimization using particle

#### Particle swarm optimization (PSO)

To enhance the predictive performance of rainfall occurrence models, Particle Swarm Optimization (PSO) was employed for hyperparameters optimization across both machine learning and deep learning frameworks. PSO is a population-based metaheuristic inspired by the collective behavior of bird flocks and fish schools^14^.

It efficiently balances exploration (searching new regions of the parameter space) with exploitation (refining around promising solutions), making it highly effective for complex and nonlinear optimization tasks such as rainfall prediction Waqas et al.^2^ Each particle in the swarm represents a candidate set of hyperparameters, with its own position $\:{x}_{i}$(t) and velocity$\:{\:v}_{i}$(t) at iteration t. These are updated iteratively based on both the particle’s own best-known solution (pbes$\:{t}_{i}$) and the global best solution found by the swarm (gbest). The governing equations are depicted in Equations (39a) and (39b).

39a $$\:\mathrm{V}\mathrm{e}\mathrm{l}\mathrm{o}\mathrm{c}\mathrm{i}\mathrm{t}\mathrm{y}\:\mathrm{U}\mathrm{p}\mathrm{d}\mathrm{a}\mathrm{t}\mathrm{e}:\:{v}_{i}\left(\mathrm{t}\hspace{0.17em}+\hspace{0.17em}1\right)=\hspace{0.17em}\mathrm{w}\cdot\:{\:v}_{i}\left(\mathrm{t}\right)+\:{c}_{1}{r}_{1}(\mathrm{p}\mathrm{b}\mathrm{e}\mathrm{s}\:{t}_{i}-\:{x}_{i}(\mathrm{t}\left)\right)+{c}_{2}{r}_{2}(gbest-{x}_{i}(t\left)\right)$$

39b $$\:\mathrm{P}\mathrm{o}\mathrm{s}\mathrm{i}\mathrm{t}\mathrm{i}\mathrm{o}\mathrm{n}\:\mathrm{U}\mathrm{p}\mathrm{d}\mathrm{a}\mathrm{t}\mathrm{e}:\:{x}_{i}(\mathrm{t}+1)={x}_{i}\left(\mathrm{t}\right)\:+{v}_{i}(\mathrm{t}+1)$$

where:

w = inertia weight (controls balance between exploration and exploitation), $\:{c}_{1}$,$\:{c}_{2}$= cognitive and social acceleration coefficients, $\:{r}_{1}$,$\:{r}_{2}$∼U(0,1) = random numbers introducing stochasticity, pbes$\:{t}_{i}$​= personal best solution of particle i, gbest = global best solution across all particles.

#### Hybrid PSO–GA hyperparameter search strategy

To optimize the deep learning architecture, a hybrid Particle Swarm Optimization–Genetic Algorithm (PSO–GA) framework was employed for automatic hyperparameter tuning of the GRU–Attention model. Particle Swarm Optimization (PSO) guides candidate solutions toward optimal regions of the search space using local-best and global-best parameter updates, while Genetic Algorithm (GA) operators improve exploration capability and prevent premature convergence. Specifically, crossover was applied to combine hyperparameter information from high-performing candidate solutions, mutation (probability = 0.25) introduced stochastic variation to avoid local optima, and elite selection preserved the top 25% performing candidates at each generation to maintain optimization stability. These mechanisms improved both convergence reliability and search diversity compared with standalone PSO. The swarm population size was set to 5 candidate solutions and evolved over 4 generations (iterations). These values were selected to balance computational efficiency with sufficient exploration of the hyperparameter space during multi-city cross-validation experiments. The optimization process terminated either after completion of all generations or when validation performance stopped improving due to early stopping (patience = 3 epochs) during candidate evaluation. The optimization objective was defined as the mean ROC-AUC across all forecast horizons computed on validation folds. During each evaluation step, candidate models were trained for up to 12 epochs with early stopping to reduce overfitting and computational cost. The hyperparameter search space included: GRU units: {32, 64, 96, 128}, Dense units: {16, 32, 64}, Dropout rate: {0.2, 0.3, 0.4, 0.5}, Learning rate: {10^-4^, 3 × 10^-4^, 5 × 10^-4^, 10^−3^}.The optimal parameter configuration selected by the hybrid PSO–GA optimizer was subsequently used to train the final rainfall occurrence classification models for each city and forecast horizon.

Mathematically, the hybrid PSO–GA optimization problem can be formulated as follows in Eq. (40a-40b).

40a $$\:{{\uptheta\:}}^{\ast}=\:{}_{{\Theta\:}\in\:{\Omega\:}}{}^{argmax}\frac{1}{H}\:\sum\:_{h=0}^{H-1}ROC-AU{C}_{val}^{\left(h\right)}\left({\uptheta\:}\right)\:$$

where H denotes the number of forecast horizons (RainDay0–RainDay5). The hybrid search process combines PSO-based global exploration with GA-based diversity-preserving refinement and can be expressed as:

40b $$\:{{\Theta\:}}^{(t+1)}=\mathrm{G}\mathrm{A}\left(\mathrm{P}\mathrm{S}\mathrm{O}\right({{\Theta\:}}^{t}\left)\right)\:$$

where $\:{{\Theta\:}}^{t}$represents the population of candidate hyperparameter configurations at iteration t.

In the GA refinement stage, crossover and mutation operators generate improved offspring solutions mathematically depicted in Eq. (41a-41b):

41a $$\:{{\uptheta\:}}^{\left(c\right)}={{\uptheta\:}}^{\left(p1\right)}+(1-)\:{{\uptheta\:}}^{\left(p2\right)},\in\:\left[\mathrm{0,1}\right]$$

41b $$\:\:{{\uptheta\:}}^{\left(m\right)}={{\uptheta\:}}^{\left(c\right)}+\epsilon\:,\epsilon\:\sim\:\mathrm{N}(0,{\sigma\:}^{2})$$

while elite selection preserves the top-performing candidate solutions at each generation to maintain convergence stability.

#### Evaluation metrics

In order to evaluate the predictive quality of both deep and machine learning models, five generally acknowledged classification measures are used. The mentioned criteria guarantee the balanced and strict assessment between imbalanced rainfall classes (rain vs. no rain), especially within the conditions of SMOTE enhancement.

#### Accuracy

Accuracy is defined as the ratio of correctly predicted cases (including true positives and true negatives) to all instances. This includes situations where the model correctly predicts the negative class (TN) and the positive class (TP), respectively. It also takes into account False Positives (FP), when the model incorrectly predicts the positive class, and False Negatives (FN), where the model is unable to identify a positive case. It shows the accuracy formula in Eq. (42):

42 $$\:\mathrm{A}\mathrm{c}\mathrm{c}\mathrm{u}\mathrm{r}\mathrm{a}\mathrm{c}\mathrm{y}=\frac{\mathrm{n}\mathrm{u}\mathrm{m}\mathrm{b}\mathrm{e}\mathrm{r}\:\mathrm{o}\mathrm{f}\:\mathrm{c}\mathrm{o}\mathrm{r}\mathrm{r}\mathrm{e}\mathrm{c}\mathrm{t}\:\mathrm{p}\mathrm{r}\mathrm{e}\mathrm{d}\mathrm{i}\mathrm{c}\mathrm{t}\mathrm{i}\mathrm{o}\mathrm{n}}{\mathrm{t}\mathrm{o}\mathrm{t}\mathrm{a}\mathrm{l}\:\mathrm{n}\mathrm{u}\mathrm{m}\mathrm{b}\mathrm{e}\mathrm{r}\:\mathrm{o}\mathrm{f}\:\mathrm{p}\mathrm{r}\mathrm{e}\mathrm{d}\mathrm{i}\mathrm{c}\mathrm{t}\mathrm{i}\mathrm{o}\mathrm{n}\mathrm{s}}$$

#### Precision

Precision is the proportion of cases that are accurately classified as positive. That example, if a model predicts positive values. The equation in Eq. (43)

43 $$\:\mathrm{P}\mathrm{r}\mathrm{e}\mathrm{c}\mathrm{i}\mathrm{s}\mathrm{i}\mathrm{o}\mathrm{n}:\:\frac{\mathrm{T}\mathrm{P}}{\mathrm{T}\mathrm{P}+\mathrm{F}\mathrm{P}}$$

#### Recall

Recall is defined as the proportion of successfully recognized positives to all positives. This formula is the same as the sensitivity formula. Equation (44) illustrates

44 $$\:\mathrm{R}\mathrm{e}\mathrm{c}\mathrm{a}\mathrm{l}\mathrm{l}:\:\frac{\mathrm{T}\mathrm{P}}{\mathrm{T}\mathrm{P}+\mathrm{F}\mathrm{N}}$$

#### F1 score

When precision and recall alone are insufficient to evaluate performance, such as when one mining technique has higher accuracy but lower recall than another, the question of which algorithm is superior may come up. The F-measure, which gives the mean of recall and precision, can be used to address this problem. The calculation’s results are shown in Eq. (45). It provides an equitable evaluation of a model’s accuracy by fusing recall and precision into a single score.

45 $$\:\mathrm{F}1\:\mathrm{s}\mathrm{c}\mathrm{o}\mathrm{r}\mathrm{e}:\frac{2\mathrm{*}\left(\mathrm{p}\mathrm{r}\mathrm{e}\mathrm{c}\mathrm{i}\mathrm{s}\mathrm{i}\mathrm{o}\mathrm{n}\mathrm{*}\mathrm{r}\mathrm{e}\mathrm{c}\mathrm{a}\mathrm{l}\mathrm{l}\right)}{\mathrm{P}\mathrm{r}\mathrm{e}\mathrm{c}\mathrm{i}\mathrm{s}\mathrm{i}\mathrm{o}\mathrm{n}+\mathrm{r}\mathrm{e}\mathrm{c}\mathrm{a}\mathrm{l}\mathrm{l}}$$

#### Roc AUC

Areas under the curve of the “Receiver Operating Characteristics” curve are referred to as AUC ROC. The AUC ROC curve is frequently used to evaluate an ML model’s performance. The AUC definition of the ROC curve quantifies a binary classifier’s ability to distinguish across several categories.

## Results and discussion

The dataset comprised 130,230 daily meteorological observations (1990–2025) collected from ten major Pakistani cities, averaging approximately 13,000 records per city. For model evaluation, a TimeSeriesSplit cross-validation with folds and random seeds per city was implemented to ensure temporal consistency and reproducibility for both ML and DL models. The time series split cross-validation folds were balanced in time, about 2,400 to 5,000 samples were used in the training files, and 1,400 to 1,500 samples were kept in the validation files. In spite of the fact that the number of training instances also grew gradually between folds - as the size of the historical window also grows with TSCV - the size of the validation stayed nearly the same that guaranteed the validation conditions to be similar. Following SMOTE on training part alone, the size of the sample increased significantly in every fold, commonly 2,400-2,600 samples in the initial fold and 11,000–11,500 samples in subsequent folds. This enabled the minority (rainy) group to be well adequately represented without spilling future information to the training set. All 130,230 daily observations in total were used in this multi-fold process, which allowed to thoroughly evaluate the model-generalization and stability in various temporal segments. The over all results of each proposed model discuss here.

### Machine learning models results with and without PSO

**Fig. 8:**
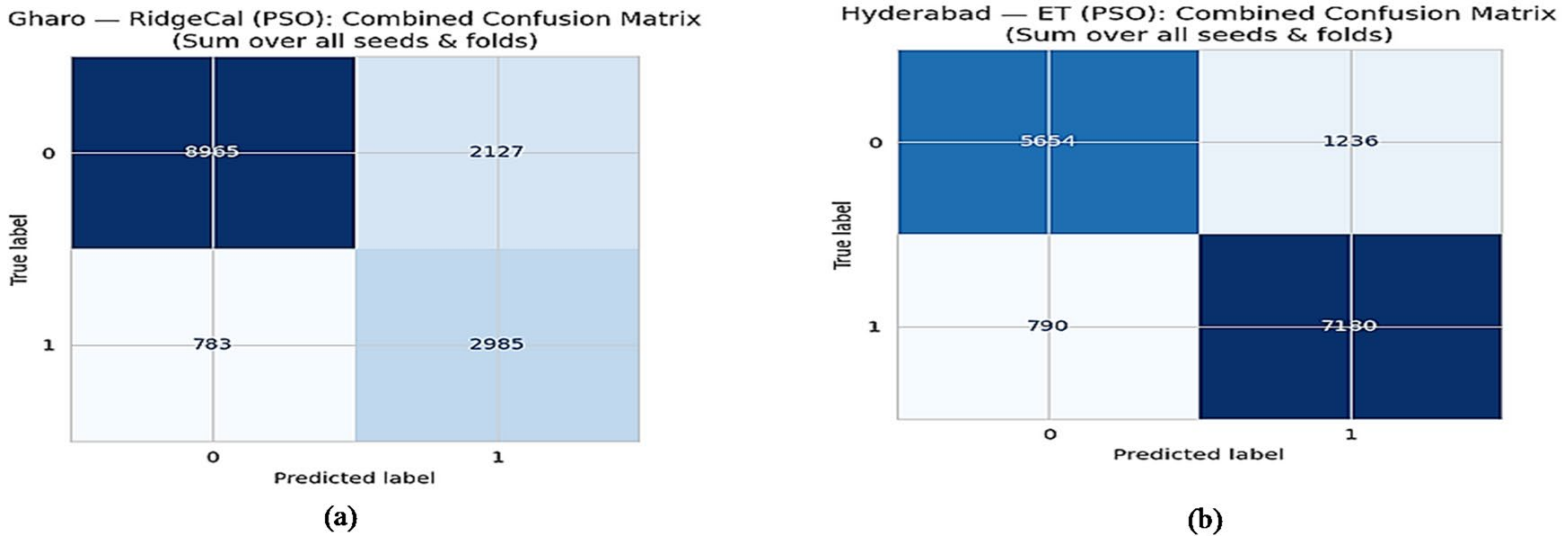
(**a**,**b**) PSO-optimized confusion matrices for machine learning models. (**a**) Gharo Ridge Cal PSO confusion metric. (**b**) Hyderabad ET PSO confusion metric.

**Fig. 9:**
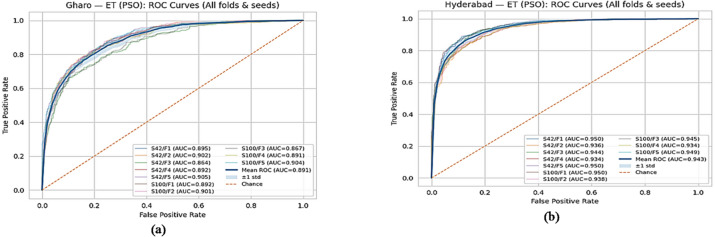
(**a**,**b**) Comparative ROC curves and confusion matrices of PSO-optimized models showing improved recall and class discrimination for representative cities (Gharo, Hyderabad, and Quetta) result reported in Table 4. (**a**) Gharo PSO-ET ROC curve results. (**b**) Hyderabad PSO-ET ROC curve results.

A comparative study of machine learning rainfall occurrence categorization models across ten Pakistani cities revealed that ensemble classifiers performed better under both baseline and PSO-optimized settings (Table 4). Hyderabad shown a notable capacity to characterize nonlinear rainfall patterns in climatically transitional regions, with the highest classification accuracy in the without-PSO setting using the HGB model (accuracy = 0.949) at fold-5 with seed-100. Similarly, ET produced consistent baseline results in Jamshoro (0.916, fold-5 seed-42) and Gharo (0.908, fold-5 seed-100). After PSO modification, improved rainfall-event detection performance was observed, particularly in Mirpurkhas using ET (accuracy = 0.837, fold-5 seed-100) and Quetta using GNB (accuracy = 0.813, fold-3 seed-42), demonstrating improved recall sensitivity and ROC–AUC stability. The strongest predictive configurations were generally formed by folds 3–5 and seeds 42–100, indicating the strong temporal universality of the proposed framework throughout Pakistan’s many climatic regions. While accuracy stayed highest in several cities without optimization, the adjustment through PSO led to enhanced generalization stability, reduced false-negative rainfall predictions, and a more consistent ROC-AUC performance across different areas. In summary, these results indicate that ensemble learners effectively capture the main nonlinear rainfall dynamics, although PSO provides further enhancements in rainfall-event sensitivity and cross-regional prediction robustness across the diverse meteorological conditions in Pakistan. As illustrated in Figs. 8a,b and 9a,b, the confusion matrices and ROC curves indicate enhanced performance in classifying rainfall occurrences post-optimization using PSO across selected cities. The results demonstrate improved class differentiation and stable prediction reliability under multi-seed TimeSeriesSplit validation, showcasing higher recall for identifying rainfall events, balanced precision-recall trade-offs, and consistently elevated ROC-AUC values.

**Table 4:** City-wise performance comparison of machine learning classifiers with and without PSO-based hyperparameter optimization for daily rainfall occurrence classification across ten Pakistani cities.

City and province	Model	Acc w/o PSO	Prec w/o PSO	Rec w/o PSO	ROC-AUC w/o PSO	F1 w/o PSO	Acc w/ PSO	Prec w/ PSO	Rec w/ PSO	F1 w/ PSO	ROC-AUC w/ PSO	ΔAcc=withPSO –w/oPSO	Fold	Seed	Performance Remark
Gharo (Sindh)	ET	0.908	0.746	0.712	0.908 ± 0.005	0.750	0.837	0.656	0.729	0.687	0.893±0.013	−0.071	5	100	Baseline preferred
	HGB	0.893	0.662	0.621	0.893 ± 0.008	0.640	0.819	0.635	0.702	0.667	0.880±0.012	−0.074	5	100	PSO improves recall
	GNB	0.841	0.601	0.545	0.841 ± 0.006	0.571	0.710	0.533	0.870	0.661	0.824±0.012	−0.131	5	42	PSO improves rainfall detection
	Ridge Cal	0.899	0.689	0.681	0.899 ± 0.004	0.684	0.834	0.688	0.811	0.745	0.899±0.006	−0.065	4	100	PSO improves F1
Hub Lasbela (Balochistan)	ET	0.869	0.676	0.641	0.869 ± 0.004	0.658	0.791	0.738	0.640	0.692	0.856±0.006	−0.078	5	100	Comparable
	HGB	0.853	0.658	0.621	0.853 ± 0.006	0.639	0.776	0.736	0.666	0.699	0.846 ± 0.005	−0.077	5	100	PSO improves F1
	GNB	0.776	0.534	0.513	0.801 ± 0.011	0.523	0.697	0.579	0.823	0.680	0.788±0.008	−0.079	5	42	PSO improves recall
	Ridge Cal	0.853	0.658	0.621	0.854 ± 0.004	0.639	0.771	0.699	0.725	0.712	0.854±0.004	−0.082	5	100	PSO improves balance
Hyderabad (Sindh)	ET	0.936	0.837	0.899	0.936 ± 0.002	0.867	0.867	0.847	0.931	0.887	0.950±0.004	−0.069	5	100	PSO improves recall robustness
	HGB	0.949	0.848	0.899	0.949 ± 0.002	0.892	0.874	0.860	0.927	0.892	0.949±0.004	−0.075	5	100	Comparable
	GNB	0.938	0.875	0.885	0.938 ± 0.006	0.880	0.864	0.875	0.885	0.880	0.938±0.006	−0.074	5	100	Comparable
	Ridge Cal	0.940	0.842	0.901	0.940 ± 0.002	0.871	0.880	0.892	0.887	0.889	0.947±0.005	−0.060	1	42	PSO improves F1
Jamshoro (Sindh)	ET	0.916	0.582	0.768	0.916 ± 0.004	0.685	0.865	0.612	0.779	0.685	0.905±0.006	−0.051	5	42	PSO improves recall
	HGB	0.907	0.632	0.741	0.907 ± 0.005	0.681	0.857	0.594	0.796	0.681	0.898±0.007	−0.050	5	100	PSO improves recall
	GNB	0.860	0.574	0.603	0.860 ± 0.006	0.588	0.661	0.346	0.863	0.494	0.835±0.009	−0.199	5	100	Baseline preferred
	Ridge Cal	0.912	0.621	0.734	0.912 ± 0.004	0.672	0.808	0.499	0.856	0.630	0.901±0.006	−0.104	5	100	PSO improves recall
Karachi (Sindh)	ET	0.838	0.626	0.647	0.838 ± 0.005	0.633	0.787	0.746	0.647	0.693	0.889±0.008	−0.051	5	100	PSO improves F1
	HGB	0.847	0.735	0.641	0.847 ± 0.006	0.684	0.773	0.728	0.671	0.698	0.881±0.009	−0.074	5	42	PSO improves recall
	GNB	0.818	0.615	0.519	0.818 ± 0.007	0.563	0.693	0.576	0.819	0.676	0.865±0.010	−0.125	5	42	PSO improves recall strongly
	Ridge Cal	0.863	0.548	0.661	0.863 ± 0.006	0.722	0.775	0.698	0.747	0.722	0.889±0.008	−0.088	5	100	Comparable
Larkana (Sindh)	ET	0.900	0.547	0.713	0.900 ± 0.004	0.678	0.821	0.572	0.727	0.640	0.889±0.006	−0.079	4	42	Baseline preferred
	HGB	0.882	0.633	0.698	0.882 ± 0.005	0.665	0.817	0.566	0.697	0.624	0.874±0.007	−0.065	4	42	Comparable
	GNB	0.831	0.581	0.523	0.831 ± 0.007	0.551	0.721	0.421	0.755	0.540	0.854±0.008	−0.110	4	42	PSO improves recall
	Ridge Cal	0.895	0.644	0.678	0.895 ± 0.004	0.661	0.773	0.489	0.837	0.616	0.889±0.006	−0.122	4	42	PSO improves recall
Nawabshah (Sindh)	ET	0.861	0.590	0.743	0.903 ± 0.013	0.656	0.861	0.590	0.743	0.656	0.903±0.013	0.000	1	42	Comparable
	HGB	0.914	0.671	0.731	0.914 ± 0.005	0.699	0.860	0.591	0.726	0.650	0.890 ± 0.020	−0.054	2	42	Baseline preferred
	GNB	0.876	0.610	0.573	0.876 ± 0.004	0.591	0.683	0.338	0.787	0.472	0.834±0.013	−0.193	5	42	Baseline preferred
	Ridge Cal	0.908	0.663	0.716	0.908 ± 0.006	0.689	0.810	0.484	0.831	0.610	0.901±0.007	−0.098	2	42	Comparable
Quetta (Balochistan)	ET	0.827	0.673	0.778	0.890 ± 0.002	0.722	0.821	0.625	0.768	0.688	0.887±0.016	−0.006	2	42	Comparable
	HGB	0.811	0.648	0.762	0.876 ± 0.004	0.722	0.811	0.613	0.728	0.664	0.871±0.022	0.000	2	42	Comparable
	GNB	0.788	0.573	0.631	0.828 ± 0.003	0.701	0.813	0.601	0.833	0.697	0.892±0.010	+ 0.025	3	42	PSO preferred
	Ridge Cal	0.816	0.642	0.751	0.869 ± 0.003	0.601	0.809	0.592	0.853	0.698	0.900±0.004	−0.007	3	42	Comparable
Thatta (Sindh)	ET	0.860	0.607	0.726	0.899 ± 0.008	0.659	0.860	0.607	0.726	0.659	0.899±0.008	0.000	2	42	Comparable
	HGB	0.849	0.583	0.702	0.885 ± 0.012	0.635	0.849	0.583	0.702	0.635	0.885±0.012	0.000	2	42	Comparable
	GNB	0.676	0.353	0.851	0.834 ± 0.014	0.495	0.676	0.353	0.851	0.495	0.834±0.014	0.000	4	42	Comparable
	Ridge Cal	0.803	0.485	0.808	0.891 ± 0.009	0.603	0.803	0.485	0.808	0.603	0.891±0.009	0.000	2	42	Comparable
Mirpurkhas (Sindh)	ET	0.834	0.746	0.712	0.894 ± 0.004	0.728	0.837	0.656	0.729	0.687	0.893±0.013	+ 0.003	5	100	PSO preferred
	HGB	0.828	0.716	0.691	0.884 ± 0.005	0.703	0.827	0.640	0.707	0.668	0.880±0.013	−0.001	5	100	Comparable
	GNB	0.806	0.598	0.564	0.858 ± 0.008	0.581	0.694	0.444	0.848	0.578	0.816±0.017	−0.112	4	42	Baseline prefered
	Ridge Cal	0.823	0.706	0.671	0.879 ± 0.006	0.688	0.803	0.575	0.794	0.664	0.889±0.012	−0.020	2	42	Comparable

### Baseline deep learning models results

Table 5 depicts results of the deep learning showing that there is a robust and competitive performance in the city with LSTM, BiLSTM, GRU, and Transformer architectures, and all the models in these architectures are trained with the most appropriate seed-fold setup, and the evaluation is based on class-weight balancing. GRU was the most efficacious of those, and it had always had high recall and AUC values. GRU demonstrated better performance in temporal learning and stability by AUC = 0.955 (Hyderabad), 0.919 (Jamshoro), and 0.915 (Thatta) in rainfall sequence modeling. LSTM also displayed great generalization, especially in Hyderabad (F1 = 0.875, AUC = 0.940) and Nawabshah (AUC = 0.910), which indicates that LSTM was able to capture long-term dependencies. BiLSTM was found to be reliable with smooth convergence and steady recall, particularly in semi-arid areas that include Quetta (AUC = 0.905) and Larkana (AUC = 0.862). Transformer models, in turn, had slightly lower accuracy (0.750.80) and AUC (0.770.87), which shows that they are inefficient with small climatic time-series datasets since they are highly sensitive to the parameters.

On the whole, the findings demonstrate the most important advantages of deep learning models to the work: these models track nonlinear temporal relations, get used to variable rainfall sequences, and are able to remember past patterns features that can be critical to realistic climate modeling. GRU and LSTM were the most effective in attaining time flexibility, high F1 (maximum 0.90) and high discrimination (AUC > 0.90) in various climatic regions. BiLSTM contributed to resilience by using a two way learning approach whereas the Transformer models were found to be scalable and could be used in future research to process longer and high dimensional meteorological data.

**Fig. 10:**
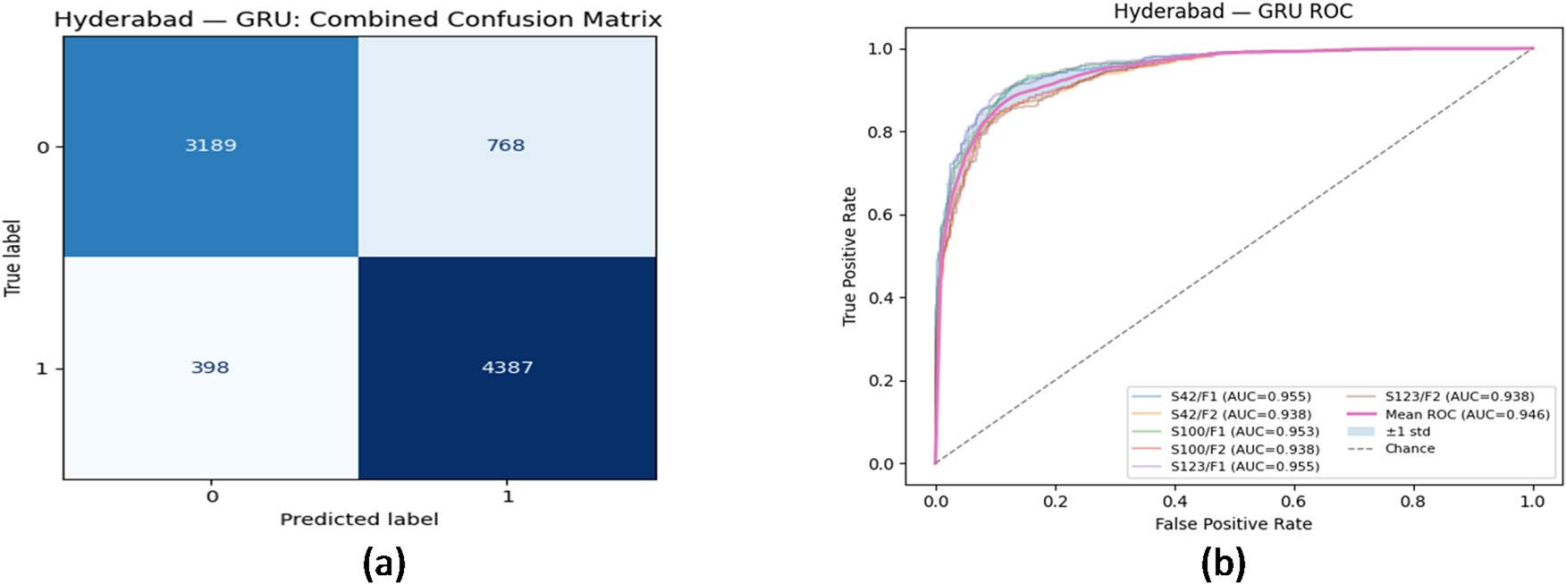
Confusion matrices for the T deep learning models of Pakistani cities by fold and seed. (**a**) Hyderabad city confusion matrix for the GRU-based rainfall occurrence. (**b**) ROC curve for the Hyderabad GRU model.

As shown in Fig. 10a–j, the GRU-based deep learning model demonstrates strong rainfall occurrence classification performance across Pakistani cities, with confusion matrices indicating high true-positive and true-negative detection rates and ROC curves showing consistently high discrimination capability (ROC–AUC ≈ 0.94–0.96These results show how GRU improves classification reliability under multi-seed Validation of TimeSeriesSplit and effectively incorporates temporal rainfall dependencies.

**Table 5:** Deep learning baseline model Result in best seed and fold across all cities and town.

City	Model	Acc	Prec	Rec	F1	ROC-AUC	Val Class1 (%)	Train Seq (f1/f2)	Val Seq	Balance	Best F1	Seed (F1)	Fold F1	Best AUC	Seed AUC
Gharo	LSTM	0.820	0.641	0.531	0.573	0.861	23.1	2401/4613	1457	CW	0.613	42	1	0.879	42
	BiLSTM	0.819	0.614	0.616	0.611	0.848	23.1				0.632	42	2	0.867	100
	**GRU**	**0.849**	**0.709**	**0.599**	**0.647**	**0.891**	23.1				**0.687**	42	2	**0.908**	42
	TRANSF	0.789	0.540	0.692	0.602	0.808	23.1				0.618	123	1	0.859	42
Hub Lasbela	LSTM	0.787	0.663	0.444	0.519	0.824	26.5	2387/4511	1457	CW	0.542	100	1	0.856	123
	**BiLSTM**	**0.794**	**0.654**	**0.489**	**0.553**	**0.819**	26.5				**0.593**	42	2	**0.878**	42
	GRU	0.803	0.721	0.458	0.539	0.871	26.5				0.572	100	1	0.872	42
	TRANSF	0.755	0.534	0.560	0.543	0.773	26.5				0.557	42	1	0.819	100
Hyderabad	LSTM	0.857	0.839	0.915	0.875	0.940	54.7	1593/3215	1457	CW	0.892	42	2	0.949	123
	BiLSTM	0.846	0.840	0.889	0.863	0.928	54.7				0.877	100	1	0.944	100
	**GRU**	**0.867**	**0.851**	**0.917**	**0.883**	**0.946**	54.7				**0.901**	100	1	**0.955**	42
	TRANSF	0.803	0.825	0.812	0.818	0.873	54.7				0.826	123	2	0.902	123
Jamshoro	LSTM	0.843	0.565	0.564	0.552	0.846	16.0	2577/4997	1457	CW	0.582	123	1	0.895	42
	BiLSTM	0.859	0.630	0.503	0.546	0.852	16.0				0.639	100	2	0.903	123
	**GRU**	**0.850**	**0.593**	**0.583**	**0.577**	**0.864**	16.0				**0.663**	100	2	**0.919**	42
	TRANSF	0.800	0.465	0.451	0.425	0.779	16.0				0.471	42	1	0.822	100
Karachi	LSTM	0.776	0.607	0.447	0.511	0.816	26.5	2387/4511	1457	CW	0.534	123	2	0.851	123
	BiLSTM	0.787	0.627	0.494	0.547	0.815	26.5				0.572	42	1	0.862	100
	**GRU**	**0.799**	**0.721**	**0.425**	**0.517**	**0.870**	26.5				0.544	42	2	0.871	100
	TRANSF	0.755	0.531	0.613	0.567	0.769	26.5				0.608	42	1	0.871	100
Larkana	LSTM	0.786	0.537	0.460	0.482	0.807	22.1	2573/4833	1457	CW	0.523	42	1	0.831	100
	BiLSTM	0.827	0.670	0.453	0.528	0.855	22.1				0.557	100	2	0.862	42
	**GRU**	**0.808**	**0.603**	**0.578**	**0.578**	**0.845**	22.1				**0.632**	123	2	**0.894**	42
	TRANSF	0.754	0.436	0.349	0.372	0.733	22.1				0.414	42	1	0.754	123
Nawabshah	LSTM	0.895	0.767	0.565	0.648	0.904	17.3	2579/4995	1457	CW	0.662	123	1	0.910	100
	BiLSTM	0.888	0.742	0.545	0.625	0.894	17.3				0.653	100	2	0.901	42
	**GRU**	**0.898**	**0.772**	**0.583**	**0.663**	**0.911**	17.3				**0.694**	123	2	**0.918**	100
	TRANSF	0.773	0.416	0.424	0.382	0.754	17.3				0.395	42	1	0.769	100
Quetta	LSTM	0.824	0.708	0.522	0.599	0.879	25.4	2471/4635	1457	CW	0.633	42	1	0.893	123
	BiLSTM	0.831	0.716	0.562	0.626	0.887	25.4				0.662	42	2	0.905	100
	**GRU**	**0.830**	**0.709**	**0.617**	**0.648**	**0.881**	25.4				**0.684**	100	2	**0.912**	123
	TRANSF	0.703	0.412	0.389	0.396	0.689	25.4				0.421	42	1	0.719	100
Thatta	LSTM	0.861	0.639	0.493	0.548	0.874	17.2	2555/4973	1457	CW	0.602	42	1	0.894	123
	BiLSTM	0.838	0.554	0.583	0.556	0.856	17.2				0.591	100	2	0.882	42
	**GRU**	**0.883**	**0.708**	**0.550**	**0.615**	**0.899**	17.2				**0.675**	123	2	**0.915**	100
	TRANSF	0.780	0.434	0.658	0.506	0.792	17.2				0.534	42	1	0.821	123
Mirpur Khas	LSTM	0.820	0.641	0.531	0.573	0.861	**23.1**	2401/4613	1457	CW	**0.605**	**42**	**1**	**0.878**	**123**
	BiLSTM	0.819	0.614	0.616	0.611	0.848	23.1				0.638	42	2	0.869	42
	**GRU**	**0.849**	**0.709**	**0.599**	**0.647**	**0.891**	23.1				**0.661**	42	2	**0.909**	100
	TRANSF	0.789	0.540	0.692	0.602	0.808	23.1				0.623	42	1	0.845	42

### Deep learning model result VMD based

The findings shown in Table 6 show that rainfall occurrence categorization performance was consistently enhanced throughout the ten cities under study when Variational Mode Decomposition (VMD) was integrated with sequential deep learning architectures. GRU-based setups outperformed all other models in a variety of climatic locations and were the most stable. In Jamshoro (0.918), Quetta (0.867), Thatta (0.896), Karachi (0.817), and Larkana (0.862), for instance, VMD-GRU generated the highest accuracy, demonstrating a remarkable ability to capture nonlinear rainfall persistence patterns from deconstructed meteorological signals. Similarly, VMD-BiLSTM demonstrated competitive performance, especially in Hyderabad, where it obtained the best overall classification balance for that city with Accuracy = 0.862, F1-score = 0.871, and ROC-AUC = 0.941.

The durability of gated recurrent architectures under extremely varied humidity-driven rainfall regimes is demonstrated by the fact that VMD-BiLSTM produced the highest F1-scores (0.618) across coastal stations like Gharo and Mirpur Khas, while VMD-GRU earned the best classification accuracy (0.867). VMD-BiGRU and VMD-GRU showed better recall–precision balance in semi-arid regions like Quetta and Nawabshah, demonstrating their efficacy in identifying minority rainfall events under class-imbalanced conditions. In the majority of cities, transformer and CNN-based models performed much worse, indicating that recurrent architectures are still better at capturing sequential rainfall persistence in decomposed meteorological time-series signals.

The consistent performance improvements observed for VMD-GRU and VMD-BiLSTM across multiple folds and heterogeneous climatic zones demonstrate that the selected VMD configuration (K = 3) improves signal representation quality and generalization capability for rainfall occurrence classification in Pakistan. In order to improve temporal feature representation without causing future information leakage, the three main intrinsic mode functions (IMF₁–IMF₃) derived from VMD decomposition were kept as denoised predictors.

**Table 6:** Performance evaluation of deep learning models (RNN, LSTM, BiLSTM, GRU) for daily rainfall classification across ten Pakistani cities.

City/town	Model	Activation	Acc	Prec	Rec	F1	ROC-AUC	Val Class1%	Train Seq	Val Seq	Remarks
Gharo	VMD + RNN	ReLU	0.866	0.744	0.473	0.578	0.882	19.5	4636	2966	–
	VMD+BiRNN		0.850	0.638	0.523	0.575	0.866				–
	VMD+LSTM		0.866	0.732	0.489	0.586	0.875				–
	VMD+BiLSTM		0.859	0.654	0.586	**0.618**	0.879				Best F1
	VMD + GRU		**0.867**	0.727	0.508	0.598	0.881				Best Acc
	VMD+BiGRU		0.851	0.643	0.523	0.577	0.880				–
	VMD+CNN1D		0.859	0.715	0.461	0.561	0.866				–
	VMD+ TRANSF		0.860	0.682	0.529	0.596	0.852				–
Hub Lasbela	VMD + RNN	ReLU	0.817	0.726	0.388	0.506	0.846	24.1	4534	2966	–
	VMD+BiRNN		0.803	0.612	0.507	0.555	0.831				–
	VMD+LSTM		0.822	0.753	0.391	**0.515**	0.848				Best F1
	VMD+BiLSTM		0.811	0.664	0.436	0.526	0.847				–
	VMD + GRU		**0.824**	0.730	0.432	0.543	0.855				Best Acc
	VMD+ BiGRU		0.807	0.648	0.443	0.526	0.839				–
	VMD+CNN1D		0.807	0.710	0.338	0.458	0.826				–
	VMD+TRANSF		0.807	0.676	0.385	0.491	0.803				–
Hyderabad	VMD + RNN	ReLU	0.861	0.867	0.867	0.867	0.938	52.1	3238	2966	–
	VMD+BiRNN		0.850	0.814	0.889	0.850	0.933				–
	VMD+LSTM		**0.862**	0.848	0.896	**0.871**	**0.941**				Best Acc & F1
	VMD+BiLSTM		0.854	0.825	0.913	0.867	0.939				–
	VMD + GRU		0.862	0.850	0.892	0.870	0.941				–
	VMD+BiGRU		0.851	0.821	0.913	0.865	0.938				–
	VMD+CNN1D		0.840	0.822	0.884	0.852	0.923				–
	VMD+TRANSF		0.837	0.815	0.887	0.850	0.916				–
Jamshoro	VMD + RNN	ReLU	0.915	0.699	0.497	0.581	0.899	11.8	5020	2966	–
	VMD+ BiRNN		0.913	0.707	0.449	0.549	0.891				–
	VMD+LSTM		**0.918**	0.695	0.546	0.611	0.906				Best Acc
	VMD+BiLSTM		0.909	0.625	0.580	**0.601**	0.900				Best F1
	VMD + GRU		0.918	0.697	0.540	0.609	0.900				–
	VMD+BiGRU		0.916	0.701	0.503	0.586	0.903				–
	VMD+CNN1D		0.897	0.564	0.557	0.560	0.877				–
	VMD+TRANSF		0.906	0.620	0.523	0.567	0.873				–
Karachi	VMD + GRU	ReLU	**0.817**	0.699	0.422	0.526	0.851	24.1	4534	2966	Best Acc
	VMD+ BiGRU		0.802	0.598	0.547	**0.571**	0.838				Best F1
	VMD+LSTM		0.815	0.715	0.388	0.503	0.843				–
	VMD+BiLSTM		0.810	0.673	0.411	0.510	0.839				–
	VMD + RNN		0.809	0.683	0.388	0.495	0.842				–
	VMD+TRANSF		0.805	0.665	0.385	0.488	0.799				–
	VMD+CNN1D		0.804	0.692	0.342	0.458	0.822				–
	VMD+BiRNN		0.794	0.587	0.496	0.537	0.832				–
Larkana	VMD + RNN	ReLU	0.856	0.653	0.477	0.551	0.877	18.6	4856	2966	–
	VMD+BiRNN		0.851	0.609	0.559	0.583	0.875				–
	VMD+LSTM		0.856	0.652	0.479	0.552	0.880				–
	VMD+BiLSTM		0.852	0.602	0.593	0.598	0.877				–
	VMD + GRU		**0.862**	0.657	0.543	0.594	0.887				Best Acc
	VMD+BiGRU		0.857	0.623	0.590	**0.606**	0.885				Best F1
	VMD+CNN1D		0.838	0.572	0.503	0.535	0.851				–
	VMD+TRANSF		0.823	0.529	0.410	0.462	0.831				–
Nawabshah	RNN	ReLU	0.895	0.699	0.547	0.614	0.897	15.3	5018	2966	–
	BiRNN		0.887	0.648	0.576	0.610	0.889				–
	VMD+LSTM		**0.900**	0.732	0.543	0.624	0.898				Best Acc
	VMD+BiLSTM		0.905	0.767	0.539	0.633	0.898				–
	VMD + GRU		0.899	0.699	0.589	0.640	0.902				–
	VMD+BiGRU		0.903	0.731	0.576	**0.644**	0.899				Best F1
	VMD+CNN1D		0.883	0.696	0.419	0.523	0.872				–
	VMD+TRANSF		0.887	0.692	0.470	0.560	0.854				–
Quetta	VMD + RNN	ReLU	0.858	0.731	0.594	0.655	0.902	22.7	4658	2966	–
	VMD+BiRNN		0.852	0.690	0.626	0.657	0.900				–
	VMD+LSTM		0.861	0.751	0.580	0.655	0.906				–
	VMD+BiLSTM		0.865	0.744	0.619	0.676	0.907				–
	VMD + GRU		**0.867**	0.742	0.632	**0.683**	**0.909**				Best Acc & F1
	VMD+BiGRU		0.859	0.707	0.649	0.676	0.905				–
	VMD+CNN1D		0.839	0.722	0.467	0.567	0.873				–
	VMD+TRANSF		0.840	0.712	0.494	0.583	0.860				–
Thatta	VMD + RNN	ReLU	0.895	0.687	0.501	0.580	0.894	14.5	4996	2966	–
	VMD+BiRNN		0.890	0.685	0.445	0.540	0.882				–
	VMD+LSTM		0.893	0.720	0.427	0.536	0.894				–
	VMD+BiLSTM		0.890	0.649	0.527	0.582	0.892				–
	VMD + GRU		**0.896**	0.674	0.550	**0.606**	0.894				Best Acc & F1
	VMD+BiGRU		0.892	0.671	0.503	0.575	0.891				–
	VMD+CNN1D		0.881	0.613	0.473	0.534	0.862				–
	VMD+TRANSF		0.879	0.624	0.410	0.495	0.861				–
Mirpur Khas	VMD + RNN	ReLU	0.866	0.744	0.473	0.578	0.882	19.5	4636	2966	–
	VMD+BiRNN		0.850	0.638	0.523	0.575	0.866				–
	VMD+LSTM		0.866	0.732	0.489	0.586	0.875				–
	VMD+BiLSTM		0.859	0.654	0.586	**0.618**	0.879				Best F1
	VMD + GRU		**0.867**	0.727	0.508	0.598	0.881				Best Acc
	VMD+BiGRU		0.851	0.643	0.523	0.577	0.880				–
	VMD+CNN1D		0.859	0.715	0.461	0.561	0.866				–
	VMD+TRANSF		0.860	0.682	0.529	0.596	0.852				–

### Hybrid deep learning model results VMD + PSO+GRU(VPG) based

Once again, the GRU competency of rainfall prediction was checked another model design using (VMD + PSO + GRU) .The findings in Table 7 (VMD + PSO + GRU) show that the performance in rainfall classification was optimum when the VMD + PSO + GRU framework was used. The most equal and correct results were on cities like: Jamshoro (Acc = 0.882), Thatta (Acc = 0.878), Nawabshah (Acc = 0.862), and Hyderabad (AUC = 0.912, F1 = 0.846). The noise-filtering property of VMD and the optimization of PSO tremendously boosted the performance of GRU in terms of capturing the temporal relationship and generalizing well to the different climatic regions of Pakistan, where the last VMD + PSO + GRU inference structure resulted in the most stable results with highest accuracy and temporal adaptation.

**Fig. 11:**
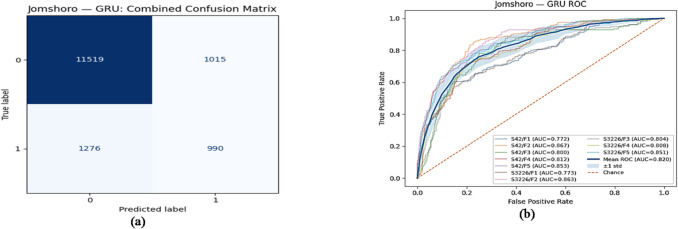
Confusion matrices and ROC curves of the GRU model for (**a**) Jamshoro Confusion Matrix, and (**b**) Jamshoro ROC Curve, based on the multi-seed the VMD + PSO + GRU binary classification results reported in Table 7.

The analysis of the confusion matrix depicted in Fig. 11a offers additional insights into the classification accuracy of the proposed VMD + PSO + GRU (VPG) architecture. The model misclassified 1,015 false-alarm rainfall occurrences and 1,276 overlooked rainfall events for Jamshoro, while accurately detecting 11,519 no-rainfall days and 990 rainfall days. These results indicate that, even with class imbalance in the validation dataset, the model preserves strong specificity for identifying dry days while also demonstrating reasonable sensitivity to rainfall events. The uneven rainfall distribution (class-1 = 10%), which inherently reduces recall yet minimally impacts general classification stability, is evident in the relatively greater quantity of missed rainfall events versus false alarms.

The ROC curve in Fig. 11b further verifies the strong separability between rainfall and non-rainfall classes across folds and seeds, as AUC values consistently exceed 0.80. This indicates that enhancing temporal feature representation via signal decomposition and adaptive hyperparameter tuning boosts threshold-independent discrimination abilities in the hybrid VMD + PSO optimization method. The model realized equitable precision–recall balances across cities such as Hyderabad (AUC = 0.912) and Gharo (AUC = 0.864), indicating improved detection of rainfall events in different climatic conditions.

**Table 7:** Performance results of the VMD + PSO + GRU framework for rainfall classification across Pakistani cities.

City	Proposed model	Best accuracy	F1	AUC	Precision	Recall	Seed	Fold	Train Seq	Val Seq	Follow-ups accuracy on fold
Gharo	VMD + PSO + GRU (VPG)	0.849	0.651	0.864	0.612	0.696	42	2	4636	1480	Best single-fold
Hub Lasbela Balochistan		0.808	0.563	0.819	0.618	0.517	42	2	4534	1480	Best single-fold accuracy.
Hyderabad		0.838	0.846	0.912	0.877	0.816	42	1	1616	1480	Strong across all metrics.
Jamshoro		**0.882**	0.171	0.804	0.310	0.118	3226	3	7596	1480	Very skewed val set (class-1 ≈ 10.3%); high Acc but low F1/Rec.
Karachi		0.807	0.593	0.819	0.599	0.588	3226	2	4534	1480	Tied on Acc with Seed = 42/Fold = 2; higher F1.
Larkana		0.816	0.280	0.766	0.414	0.212	3226	3	7228	1480	Skewed validation; Acc high F1/Rec low
Nawabshah		0.862	0.498	0.822	0.580	0.435	42	2	5018	1480	Best single-fold accuracy.
Quetta		0.783	0.389	0.752	0.500	0.318	3226	3	6926	1480	Best single-fold accuracy.
Thatta		**0.878**	0.480	0.867	0.697	0.366	3226	2	4996	1480	Best single-fold accuracy.
Mirpur Khas		0.849	0.651	0.864	0.612	0.696	42	2	4636	1480	Best single-fold accuracy.

### Multi-horizon rainfall occurrence classification using a PSO-GA tuned VMD-GRU-attention hybrid model

The multi-horizon rainfall occurrence classification framework employs three forecasting scales to evaluate predictive capability: RainDay0 (prediction for the same day), RainDay1–RainDay3 (predictions for one to three days ahead), and RainDay5 (prediction for five days ahead). According to the findings in Table 8, RainDay0 demonstrated the highest prediction accuracy with the lowest level of temporal uncertainty. Hyderabad, for example, attained 87.22% accuracy with ROC-AUC = 0.946 and PR-AUC = 0.955, showcasing excellent short-term rainfall detection ability. Similarly, Thatta, Mirpur Khas, and Quetta exhibited consistent short-range classification in both inland and coastal climate conditions, achieving accuracy rates above 81%.As meteorological variability grew, the performance for medium-range forecasting (RainDay1–RainDay3) remained stable with a minor decline. With ROC-AUC and PR-AUC scores surpassing 0.84, cities such as Thatta (80.83%) and Mirpur Khas (80.44%) exhibited strong predictive reliability, showcasing effective discrimination between rainfall and non-rainfall events at mid-range lead times.

Long-range forecasting was made more difficult by diminished persistence signals and changing synoptic conditions (RainDay5). The suggested hybrid VMD–GRU–Attention–PSO–GA model maintained good predictive performance in Hyderabad (80.83%), Thatta (80.14%), Gharo (79.89%), and Mirpur Khas (79.90%). In arid continental regions like Quetta (70.13%) and Larkana (69.94%), performance remained good with ROC-AUC values over 0.74, indicating the hybrid model’s resilience for extended forecasting periods. Variations in rainfall prediction among cities are a sign of known climate inequalities. Sparse and erratic rainfall is associated with poorer accuracy in arid areas like Quetta and Larkana, reducing the temporal persistence signals necessary for sequence learning. On the other hand, coastal and deltaic regions show higher prediction reliability because of their more reliable seasonal precipitation patterns and improved moisture-driven rainfall processes.

**Fig. 12:**
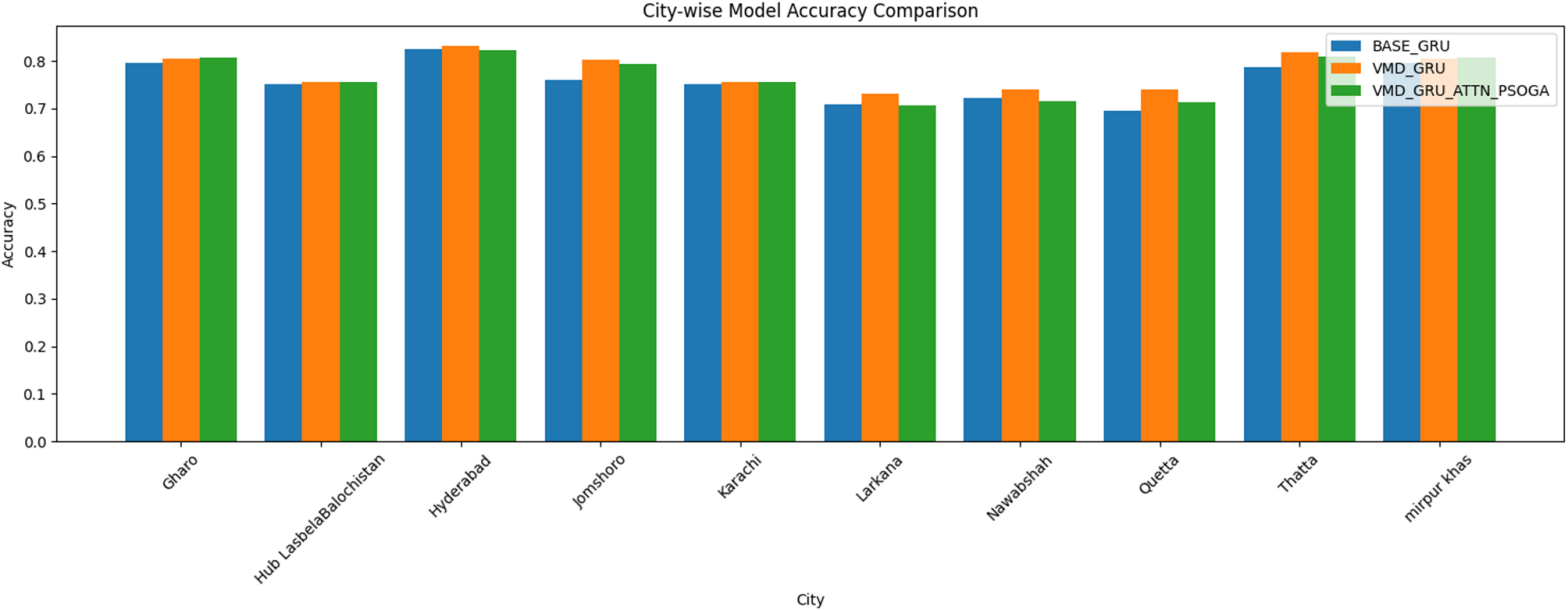
Results comparison multi-horizon rainfall occurrence classification using a PSO-GA tuned VMD-GRU-attention hybrid model across cities.

Misclassification analysis also showed low false-alarm rates (0.15–0.21) and miss rates below 0.30, indicating balanced prediction performance without persistent overestimation. The precise identification of dry-day conditions was backed by high specificity (> 0.80), strong negative predictive values (> 0.90 in many cities), and well-calibrated probability estimates indicated by Brier scores within strong (≤ 0.15) and good (0.15–0.18) ranges. Generally speaking, short-range forecasts (RainDay0) were the most reliable, medium-range forecasts (RainDay1–RainDay3) maintained constant discriminative performance, and long-range forecasts (RainDay5) remained operationally relevant despite increased atmospheric uncertainty. Figure 12 displays the model’s overall comparison among cities.

A systematic ablation study within the sequential modeling category is presented through the staged comparison of BASE-GRU, VMD-GRU, and the proposed VMD–GRU–Attention–PSO–GA framework. Performance improvements can be linked to the gradual impacts of VMD-based signal decomposition, attention-focused feature enhancement, and combined PSO–GA hyperparameter optimization, as all architectures were evaluated using comparable sequence-based feature representations and uniform chronological TimeSeriesSplit validation configurations. This controlled comparison illustrates the combined impact of these enhancements on multi-horizon rainfall occurrence prediction across forecast lead times (RainDay0–RainDay5).

**Table 8:** Multi-horizon rainfall occurrence classification using a PSO-GA tuned VMD-GRU-attention hybrid model.

City	Target	Model	Train_’acc	Val_acc	acc	CI95%	Precision	Recall	F1
Gharo	RainDay0	VMD_GRU	0.796726	0.81268	0.81268	(0.806–0.821)	0.594913	0.772695	0.670112
Gharo	RainDay1	VMD_GRU_ATTN_PSOGA	0.795748	0.811534	0.811534	(0.796–0.817)	0.6027323	0.691693	0.6436867
Gharo	RainDay2	VMD_GRU	0.786118	0.80080	0.80080	(0.793–0.810)	0.582375	0.7053125	0.635896
Gharo	RainDay3	VMD_GRU	0.789807	0.80449	0.80449	(0.796–0.812)	0.591686	0.6963959	0.637433
Gharo	RainDay4	VMD_GRU	0.7887686829	0.79884659	0.79884659	(0.790–0.807)	0.5854239302	0.67067990823	0.6223139572
Gharo	RainDay5	VMD_GRU_ATTN_PSOGA	0.77291565963298	0.79896193	0.79896193	(0.791–0.807)	0.575056	0.7012845697	0.631917
Hub Lasbela Balochistan	RainDay0	VMD_GRU	0.7774662952	0.77612456	0.77612456	(0.767–0.786)	0.5930024221	0.71592697751	0.644629764
Hub Lasbela Balochistan	RainDay1	VMD_GRU	0.770260	0.76228	0.76228	(0.752–0.771)	0.57209694	0.6963813	0.624518
Hub Lasbela Balochistan	RainDay2	VMD_GRU	0.7640358161	0.75178777	0.75178777	(0.741–0.761)	0.559294	0.6662522621	0.604092
Hub Lasbela Balochistan	RainDay3	VMD_GRU	0.7650145730	0.75928	0.75928	(0.748–0.769)	0.5772347689	0.6318194681	0.5988830503
Hub Lasbela Balochistan	RainDay4	VMD_GRU	0.7643230899	0.75351787	0.75351787	(0.744–0.763)	0.566264	0.6169084076	0.5875543718
Hub Lasbela Balochistan	RainDay5	VMD_GRU_ATTN_PSOGA	0.7424187313	0.74555940	0.74555940	(0.742–0.759)	0.5449077266	0.6446446132	0.5901343088
Hydera-bad	RainDay0	BASE_GRU	0.872015	0.87220	0.87220	(0.867–0.880)	0.87752945	0.88629518957	0.8818649837
Hydera-bad	RainDay1	VMD_GRU	0.8483810886	0.84648212	0.84648212	(0.841–0.854)	0.8768535	0.8309635	0.8531807
Hydera-bad	RainDay2	VMD_GRU	0.8418114003	0.83241061	0.8324106	(0.814–0.829)	0.8683248738	0.81133328971	0.8385432044
Hydera-bad	RainDay3	VMD_GRU	0.8306275435	0.81926182	0.8192618	(0.799–0.816)	0.8789972894	0.77022542395	0.8203504423
Hydera-bad	RainDay4	VMD_GRU	0.823535	0.81637	0.81637	(0.795–0.812)	0.869462	0.7750475294	0.819167
Hydera-bad	RainDay5	VMD_GRU	0.816847	0.80830	0.80830	(0.79–0.807)	0.862511	0.76552173	0.810987
Jomshoro	RainDay0	VMD_GRU	0.8210555786	0.82364475	0.82364475	(0.867–0.880)	0.453976834	0.81247501928	0.5824096431
Jomshoro	RainDay1	VMD_GRU	0.805201757	0.8041522	0.8041522	(0.841–0.854)	0.4160404641	0.72626483738	0.5286142590
Jomshoro	RainDay2	VMD_GRU	0.8024918599	0.79907727	0.79907727	(0.814–0.829)	0.4024300747	0.6739541520	0.5036813400
Jomshoro	RainDay3	VMD_GRU	0.7967264425	0.8103806	0.81038062	(0.799–0.816)	0.416870246	0.62973502865	0.5006803042
Jomshoro	RainDay4	VMD_GRU	0.785660	0.80853	0.80853	(0.795–0.812)	0.4127381170	0.602660037808585	0.4879189137
Jomshoro	RainDay5	VMD_GRU	0.7772972332	0.7975778	0.79757785	(0.790–0.807)	0.3919053017	0.60453822061	0.475065113
Karachi	RainDay0	VMD_GRU	0.7781582570	0.77658592	0.77658592	(0.767–0.786)	0.5936912051	0.71862797395	0.6459717549
Karachi	RainDay1	VMD_GRU	0.770952	0.76216	0.76216	(0.752–0.771)	0.5722687090	0.6974419288	0.624782
Karachi	RainDay2	VMD_GRU	0.7649005888	0.7515570	0.75155709	(0.741–0.760)	0.5587356083	0.66870819891	0.6047500141
Karachi	RainDay3	VMD_GRU	0.7653032826	0.75882352	0.75882352	(0.748–0.768)	0.5764884122	0.6332265976	0.5989683119
Karachi	RainDay4	VMD_GRU	0.765015	0.75317	0.75317185	(0.743–0.763)	0.565770808	0.6166786	0.587136
Karachi	RainDay5	VMD_GRU_ATTN_PSOGA	0.7421303408	0.74555940	0.74555940	(0.742–0.759)	0.5448859743	0.6432401188	0.5895678255
Larkana	RainDay0	BASE_GRU	0.8208231433	0.79457900	0.79457900	(0.801–0.819)	0.5058057955	0.8472014645	0.6327738776
Larkana	RainDay1	VMD_GRU	0.7675032025	0.74106113	0.74106113	(0.732–0.750)	0.4355991814	0.76110589378	0.5535014022
Larkana	RainDay2	VMD_GRU_ATTN_PSOGA	0.743348313	0.71418685121	0.71418685121	(0.704–0.724)	0.3932022879	0.6644394585	0.4937991802
Larkana	RainDay3	VMD_GRU	0.7368904554	0.71926182	0.71926182	(0.710–0.728)	0.3988623568	0.65162676937	0.4940184001
Larkana	RainDay4	VMD_GRU	0.7308962719	0.7107266	0.71072664	(0.701–0.720)	0.3811994936	0.60717152486	0.467863984
Larkana	RainDay5	VMD_GRU_ATTN_PSOGA	0.7051868815	0.69942329873	0.69942329	(0.690–0.708)	0.3702948685	0.6016098993	0.454215285
Nawab’shah	RainDay0	BASE_GRU	0.8446898814	0.8051903	0.80519031	(0.796–0.814)	0.4769534703	0.86332808058	0.610428510
Nawab shah	RainDay1	VMD_GRU	0.809989455	0.7500576	0.7500576	(0.741–0.759)	0.393721	0.7618109627	0.517022
Nawab shah	RainDay2	VMD_GRU	0.7899293	0.73783	0.7378	(0.728–0.746)	0.3714430247	0.7013837391	0.485340083
Nawab shah	RainDay3	VMD_GRU	0.7819707868	0.7336793	0.73367	(0.719–0.737)	0.3579483	0.6402690231	0.45820015
Nawab shah	RainDay4	VMD_GRU	0.76514243	0.71880046	0.7188004	(0.712–0.730)	0.340230894	0.6332885304	0.4425014940
Nawab shah	RainDay5	VMD_GRU	0.7379270819	0.70484429	0.70484429	(0.700–0.718)	0.3272625207	0.6370015037	0.4319460749
Quetta	RainDay0	BASE_GRU	0.796260	0.81257	0.81257	(0.812–0.830)	0.586311	0.8432086825	0.690664
Quetta	RainDay1	BASE_GRU	0.7541218187	0.74452133	0.74452133	(0.798–0.815)	0.4904569538	0.7510853212	0.5933725777
Quetta	RainDay2	VMD_GRU_ATTN_PSOGA	0.7108880481	0.71349480	0.71349480	(0.785–0.802)	0.4515818515	0.6648463356	0.5378172630
Quetta	RainDay3	VMD_GRU	0.7240949459	0.71026528	0.71026528	(0.772–0.790)	0.4466450500	0.6426691285	0.5268585696
Quetta	RainDay4	VMD_GRU	0.7143486546	0.71741637	0.71741637	(0.764–0.782)	0.4530616961	0.6200674152	0.5235692114
Quetta	RainDay5	VMD_GRU	0.6914026443	0.70126874	0.70126874	(0.756–0.774)	0.431889692	0.62770895	0.5116482636
Thatta	RainDay0	VMD_GRU	0.8120073112	0.81026528	0.81026528	(0.809–0.827)	0.4973745622	0.7970944009	0.6085242052
Thatta	RainDay1	VMD_GRU	0.8067027763	0.80957324	0.80957324	(0.796–0.812)	0.4933982911	0.737556354	0.5891534656
Thatta	RainDay2	VMD_GRU	0.7940206926	0.7987312	0.79873125	(0.784–0.801)	0.4753341932	0.7255598455	0.5720021269
Thatta	RainDay3	VMD_GRU	0.7909077022	0.80830449	0.80830449	(0.773–0.790)	0.490068343	0.7134124995	0.5799002021
Thatta	RainDay4	VMD_GRU	0.797246	0.81753	0.81753	(0.764–0.781)	0.509620	0.6793239393	0.580194
Thatta	RainDay5	VMD_GRU	0.7833535138	0.80138408	0.80138408	(0.756–0.773)	0.4792961962	0.7038975817	0.5683790059
mirpur khas	RainDay0	VMD_GRU	0.7967264425	0.8126874	0.81268742	(0.821–0.838)	0.594913771	0.7726958461	0.6701123833
mirpur khas	RainDay1	VMD_GRU_ATTN_PSOGA	0.7957483237	0.81153402	0.81153402	(0.806–0.823)	0.6027323537	0.6916931333	0.64368674
mirpur khas	RainDay2	VMD_GRU	0.7861181703	0.80080738	0.80080738	(0.793–0.810)	0.582375365	0.70531255145	0.635896828
mirpur khas	RainDay3	VMD_GRU	0.789807383	0.80449826	0.80449826	(0.782–0.799)	0.5916860916	0.6963959377	0.637433448
mirpur khas	RainDay4	VMD_GRU	0.788768	0.79884659	0.7988465	(0.773–0.790)	0.58542393	0.6706799082	0.622313
mirpur khas	RainDay5	VMD_GRU_ATTN_PSOGA	0.772915	0.79896	0.79896	(0.764–0.781)	0.575056	0.701284	0.631917

City	Roc_auc	pr_auc	Brier	False_alarm_rate	Miss rate	Specificity	npv	Brier interpretation	
Gharo	0.889703	0.7691430	0.143367	0.173102	0.227304	0.826897	0.9164	Very strong (≤ 0.15)	
Gharo	0.846613	0.7200402	0.1434674	0.149341	0.3083068	0.850658	0.8933857576	Very strong (≤ 0.15)	
Gharo	0.848104	0.7281191	0.149341	0.166840	0.294687	0.833159	0.895182	Very strong (≤ 0.15)	
Gharo	0.845698	0.7209640	0.156538	0.158910	0.303604	0.84108958	0.893290	Good (0.151–0.18)	
Gharo	0.8239220617	0.6992046042068	0.1670169950	0.1574112273853	0.3293200917	0.84258877261	0.88504147643707	Good (0.151–0.18)	
Gharo	0.828506	0.6696633938	0.153872	0.1704399292775	0.298715	0.8295600707	0.8947836508	Good (0.151–0.18)	
Hub Lasbela Balochistan	0.8588013163	0.7422339416420	0.15361922	0.1967871951029	0.2840730224	0.8032128048970	0.8739067288	Good (0.151–0.18)	
Hub Lasbela Balochistan	0.836899	0.717694011822	0.16227780	0.208794273794	0.303618	0.79120572	0.8648868450	Good (0.151–0.18)	
Hub Lasbela Balochistan	0.8148402274	0.697344947539	0.1717389919	0.211104562295	0.3337477378	0.78889543770495	0.8531180516183110	Good (0.151–0.18)	
Hub Lasbela Balochistan	0.8045219879	0.68388278873047	0.1783989791	0.186502934117	0.368180	0.81349706588273	0.8445532446868960	Good (0.151–0.18)	
Hub Lasbela Balochistan	0.7910648687	0.66775687377136	0.1828866271	0.189544085783	0.3830915923	0.8104559142169	0.838700566884213	Acceptable (0.181–0.20)	
Hub Lasbela Balochistan	0.781595015	0.6260322870310	0.1837440406	0.215602933794	0.355355	0.7843970662	0.8456982684569830	Acceptable (0.181–0.20)	
Hydera-bad	0.945576426	0.9553089016210	0.093359	0.1440951072408	0.113704	0.8559048927	0.8657797148684080	Very strong (≤ 0.15)	
Hydera-bad	0.923431	0.933357874	0.112586	0.13593207	0.169036	0.8640679285	0.8152782127	Very strong (≤ 0.15)	
Hydera-bad	0.907938	0.920142918	0.12347801	0.143860	0.188666710	0.856139536	0.7971243618586140	Very strong (≤ 0.15)	
Hydera-bad	0.8995787964	0.913706630	0.12999764	0.124747	0.229774576	0.87525227981117	0.7675853441394980	Very strong (≤ 0.15)	
Hydera-bad	0.895546	0.9097831592	0.132009	0.1364044166068	0.224952	0.863595583	0.7683747784	Very strong (≤ 0.15)	
Hydera-bad	0.887622	0.9028405642	0.13644	0.1425110882041	0.234478	0.8574889117	0.7590317533	Very strong (≤ 0.15)	
Jomshoro	0.897686583	0.69290216802299	0.1329912192	0.1743717065568	0.1875249807	0.8256282934	0.9610940101932680	Very strong (≤ 0.15)	
Jomshoro	0.8584847901	0.602754989	0.14376711	0.181985575658	0.2737351626	0.818014424	0.9438405752545960	Very strong (≤ 0.15)	
Jomshoro	0.8276071060	0.5595038527754	0.1543011816	0.1786089868276	0.3260458479	0.8213910131	0.9339493420757170	Good (0.151–0.18)	
Jomshoro	0.8091290374	0.53395973299180	0.1574200258	0.157407348559	0.3702649713	0.8425926514	0.9275240699201560	Good (0.151–0.18)	
Jomshoro	0.7888509789	0.4955103145483610	0.1706159037	0.1547104555894750	0.3973399621	0.8452895444	0.9228225312018030	Good (0.151–0.18)	
Jomshoro	0.7841249051	0.48627163766006	0.1705550399	0.1679146326909	0.3954617793	0.8320853673	0.9217815793801510	Good (0.151–0.18)	
Karachi	0.8587809582	0.7421719972419800	0.1539522872	0.19705397081006300	0.2813720260	0.8029460291	0.8748377389275150	Good (0.151–0.18)	
Karachi	0.836847	0.7176719001	0.162656	0.2091559633278	0.302558	0.7908440366	0.8651977401	Good (0.151–0.18)	
Karachi	0.8148582285	0.697426749	0.171873118	0.2123857951439	0.3312918010	0.7876142048	0.8538212319233170	Good (0.151–0.18)	
Karachi	0.8047300666	0.68401283234778	0.1786938514	0.1875928815740	0.366773402	0.8124071184	0.8448366167655520	Good (0.151–0.18)	
Karachi	0.790534	0.667394390	0.18342247	0.18986932	0.383321312	0.8101306798	0.8385182631749210	Acceptable (0.181–0.20)	
Karachi	0.7815696595	0.62611860642159	0.1835708454	0.2151437969713	0.3567598811	0.7848562030	0.845335133713438	Acceptable (0.181–0.20)	
Larkana	0.8960526596	0.7521596628249	0.144295439	0.2191093011339	0.1527985354	0.7808906988	0.950616961336652	Very strong (≤ 0.15)	
Larkana	0.8217532380	0.58129776307578	0.1730585109	0.2653568123869	0.2388941062	0.7346431876	0.9205972175093320	Good (0.151–0.18)	
Larkana	0.773510473	0.52448286505919	0.1892291656028	0.2736255507268	0.3355605414484	0.726374449	0.8909772745523170	Acceptable (0.181–0.20)	
Larkana	0.7605778092	0.50907411106351	0.1899804560	0.2642043402657	0.3483732306	0.7357956597	0.8887530769883710	Acceptable (0.181–0.20)	
Larkana	0.7421880134	0.48090779321341	0.200684843	0.2631578230124	0.392828475	0.7368421769	0.8766994315719420	Moderate (> 0.20)	
Larkana	0.7303702191	0.44627645607039	0.1990661160047	0.2778417708840	0.3983901006	0.7221582291	0.8757823207211660	Acceptable (0.181–0.20)	
Nawab’shah	0.9146809551	0.76675405396	0.134764467	0.205949860879	0.1366719194	0.7940501391	0.9639573651594280	Very strong (≤ 0.15)	
Nawab shah	0.8412372541	0.592952088550	0.17380743	0.251054424221	0.23818903	0.7489455757	0.9351719918497780	Good (0.151–0.18)	
Nawab shah	0.8043613125	0.54437202	0.183091	0.25399392044	0.298616260	0.7460060795	0.9198743685	Acceptable (0.181–0.20)	
Nawab shah	0.7822700731	0.5094902779137	0.1875965724	0.245570546259	0.359730	0.754429453	0.9064309346	Acceptable (0.181–0.20)	
Nawab shah	0.7552516532	0.4679143845761	0.2004113595	0.2632395758358	0.3667114695	0.736760424	0.9033819569	Moderate (> 0.20)	
Nawab shah	0.7524113446	0.45205679	0.2010804387	0.2807015621221	0.3629984962	0.7192984378	0.9021630228	Moderate (> 0.20)	
Quetta	0.906838	0.8074525170	0.12843	0.1973560102930	0.156791	0.8026439897	0.9391544017	Very strong (≤ 0.15)	
Quetta	0.8323389520	0.6554316451282	0.1671981503	0.2588583943539	0.2489146787	0.7411416056	0.900489459	Good (0.151–0.18)	
Quetta	0.7697917950	0.5557680624837	0.1860510516	0.2711448222360	0.3351536643	0.7288551777	0.8656677426718	Acceptable (0.181–0.20)	
Quetta	0.7587681220	0.5422313304196	0.1899166385	0.268557325419	0.3573308714	0.7314426745	0.8588299712630	Acceptable (0.181–0.20)	
Quetta	0.7531115	0.5407516985	0.199939	0.2506325328568	0.379932	0.7493674671	0.8541571862845	Acceptable (0.181–0.20)	
Quetta	0.7449346544	0.522895137533	0.1955490312	0.274860698927	0.3722910449	0.7251393010	0.8535330211069	Acceptable (0.181–0.20)	
Thatta	0.8962881050	0.7276942583224	0.1420624026	0.1849159864765	0.2029055990	0.8150840135	0.9447149893891	Very strong (≤ 0.15)	
Thatta	0.8669767376	0.68107366574	0.1398928406	0.173356771160	0.2624436453	0.8266432288	0.9315942531672	Very strong (≤ 0.15)	
Thatta	0.844834820	0.6490962301309	0.155009	0.1839163476626	0.2744401544	0.8160836523	0.9279418599738	Good (0.151–0.18)	
Thatta	0.8403652913	0.628924223118	0.1573001766	0.1698734015819	0.2865875004	0.830126598418	0.9261398947390	Good (0.151–0.18)	
Thatta	0.833587	0.6324167615	0.155999	0.149902813328	0.320676	0.8500971866	0.9194298651	Good (0.151–0.18)	
Thatta	0.8307564756	0.6297499386477	0.1551592999	0.1755470274006	0.2961024182	0.8244529725	0.9229307925	Good (0.151–0.18)	
mirpur khas	0.8897033486	0.769143033730	0.1433673132	0.173102699165	0.227304153	0.8268973008	0.9164228677	Very strong (≤ 0.15)	
mirpur khas	0.8466139970	0.720040265921	0.1434674526	0.149341758716	0.3083068666	0.8506582412	0.8933857576	Very strong (≤ 0.15)	
mirpur khas	0.8481045006	0.728119157485	0.1493415207	0.166840823690	0.2946874485	0.8331591763	0.89518236477	Very strong (≤ 0.15)	
mirpur khas	0.8456987886	0.7209640454261	0.1565381278	0.1589104171092	0.3036040622	0.8410895828907	0.8932905225297	Good (0.151–0.18)	
mirpur khas	0.8239220617	0.6992046042068	0.167016995	0.1574112273853	0.329320	0.8425887726	0.8850414764	Good (0.151–0.18)	
mirpur khas	0.828506	0.6696633	0.153872	0.1704399	0.298715	0.829560	0.89478	Good (0.151–0.18)	

**Fig. 13:**
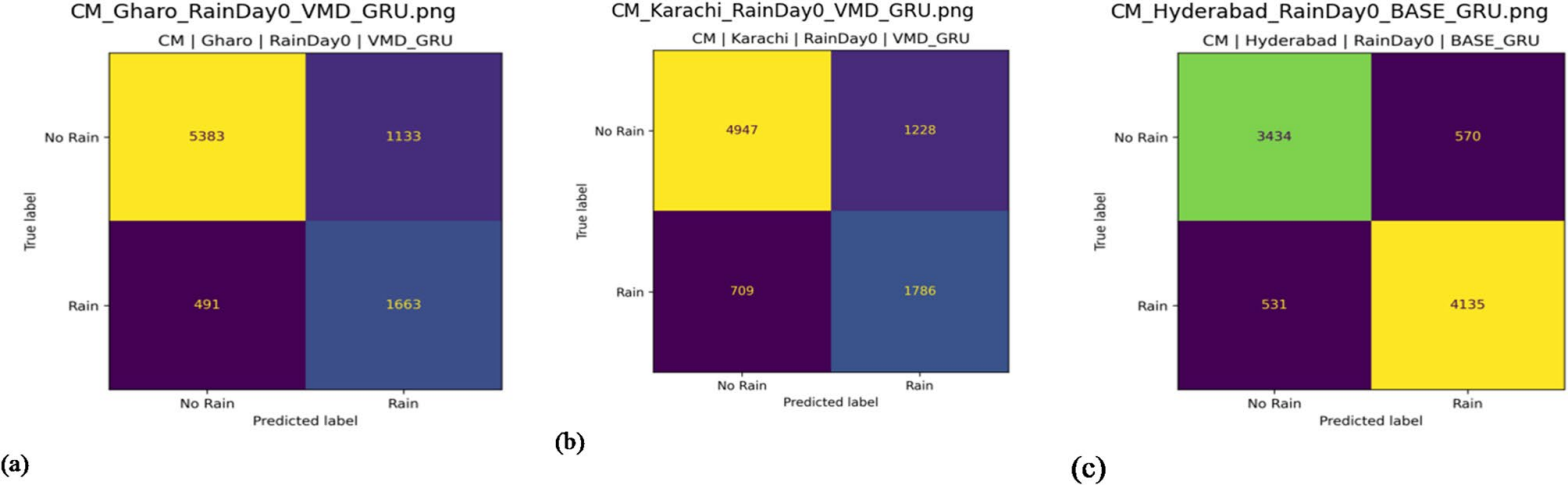
Confusion matrices for short-horizon rainfall occurrence classification (RainDay0) in (**a**) Gharo (**b**) Karachi, and (**c**) Hyderabad.

Figure 13a-c shows the confusion matrices of the prediction of short-horizon rainfall occurrence (RainDay0) in Gharo, Karachi and Hyderabad using GRU-based models. The VMD-GRU model when used in Gharo was able to measure 5,383 and 1,663 no-rain and rainfall events respectively with a relatively small number of false alarms (491), which proved that the model possesses strong rainfall detection potential in moisture-dominated environments. In Karachi, the model obtained a similar reliable classification and predicted 4,947 dry days and 1,786 rainfall detections with a slightly higher rate of false alarms (1,228) because of the urban complexity of the coastal atmosphere. The highest performance was achieved at Hyderabad where the BASE-GRU model was able to correctly classify 3,434 no-rain and 4,135 rainfall events with very low misclassification (570 false alarms and 531 misses) implying better classification balance and sensitivity in inland climatic conditions. All in all, the confusion matrices affirm that the proposed GRU-based framework is capable of predicting short-term occurrence of rainfall in the coastal and inland areas in a stable and correct manner.

**Fig. 14:**
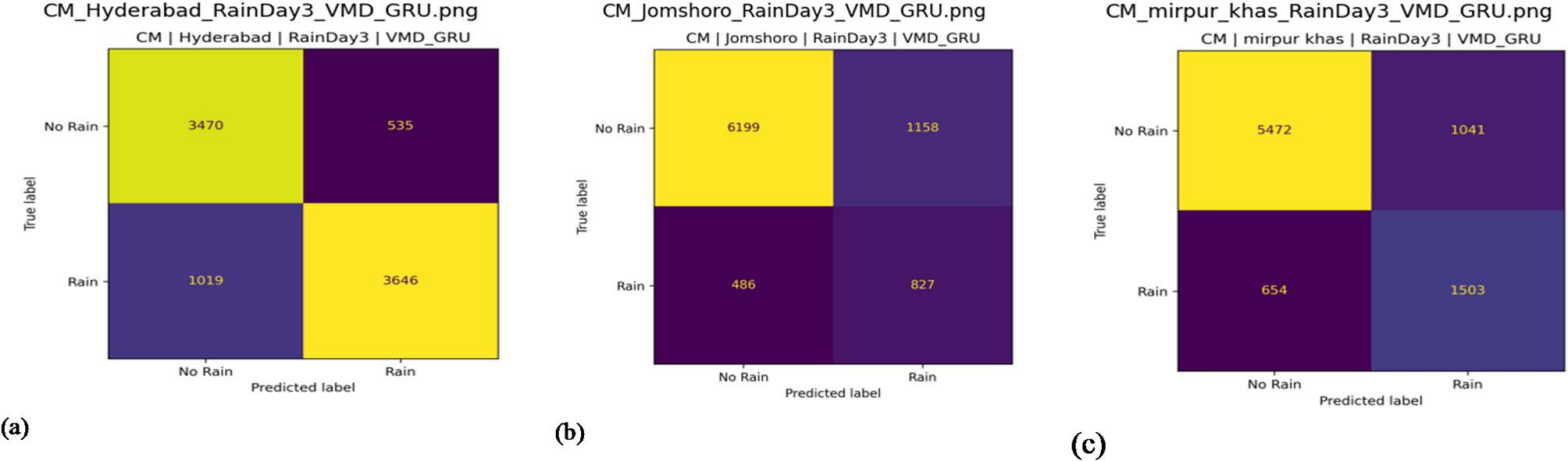
Confusion matrices for medium-horizon rainfall occurrence classification (RainDay3) in (**a**) Hyderabad, (**b**) Jomshoro, and (**c**) Mirpur Khas.

The confusion matrices of medium-horizon rainfall occurrence prediction (RainDay3) of Hyderabad, Jomshoro, and Mirpur Khas with VMD-GRU model are provided in Fig. 14d-f. The classifier in Hyderabad was able to correctly predict 3,470 no-rain events and 3,646 rain events with moderate misclassification (535 false alarms and 1,019 missed rainfalls), which showed consistent predictive performance at long lead time. The model made good dry-day detection in Jomshoro, with 6,199 true no-rain and 827 rainfall with some false alarms (1,158), indicating good discrimination under semi-arid regional variability. The model showed balanced performance in Mirpur Khas with 5,472 correct no-rain forecasts and 1,503 rainforecasts that ranged across moisture sensitive inland conditions, confirming the similarity of medium-range forecasting performance. On the whole, these findings indicate that hybrid VMD-GRU architecture is effective in maintaining temporal rainfall dependency at three-day prediction horizons.

**Fig. 15:**
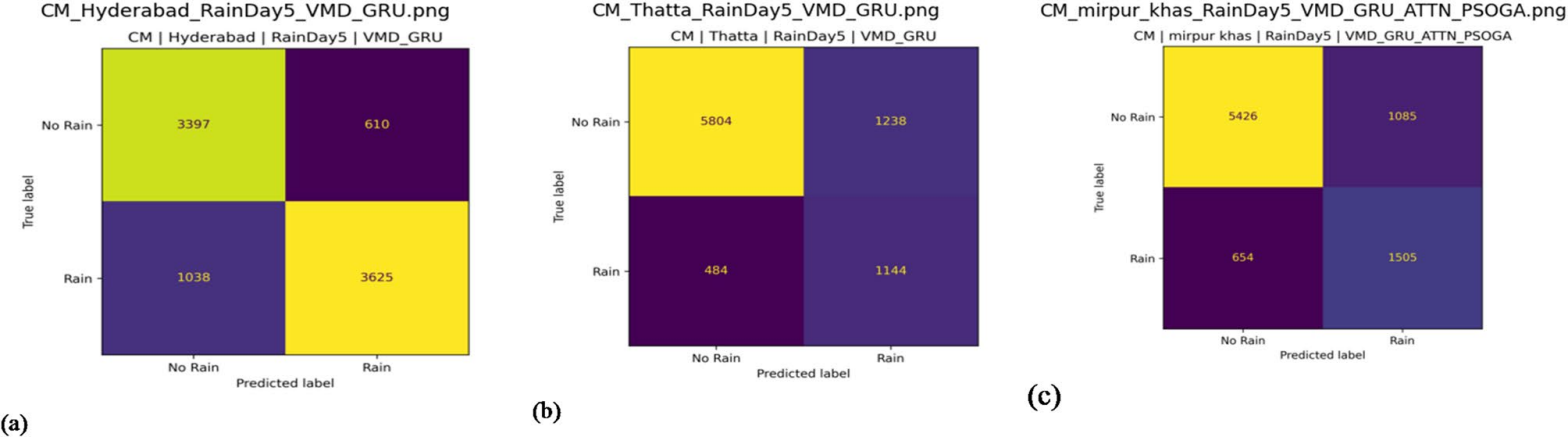
Confusion matrices for long-horizon rainfall occurrence classification (RainDay5) in (**a**) Hyderabad, (**b**) Thatta, and (**c**) Mirpur Khas.

The confusion matrices of RainDay5 predicting long-horizon rainfall occurrence in Hyderabad, Thatta and Mirpur Khas are shown in Fig. 15g-i. The models continued to give consistent classification even though their predictive ability at longer lead times had been found to be uncertain. The classifier was able to identify 3,397 no-rain events and 3,625 rainfall events with controlled misclassification (610 false alarms and 1,038 misses) in Hyderabad indicating an excellent ability to learn over time. Likewise, the model demonstrated strong results in dry-day detection in Thatta, with 5,804 no-rain and 1,144 rainfall predictions, demonstrating the dependability in coastal moisture-dominated weather. The optimized architecture in terms of long-term rainfall forecasting was tested with the hybrid VMD-GRU-ATTN-PSOGA model, which yielded balanced predictions of 5,426 correct no-rain events and 1,505 rainfall detections in Mirpur Khas. The findings reveal that the hybrid framework proposed has predictive strength even in five-day lead horizons in different climatic regions.

**Fig. 16:**
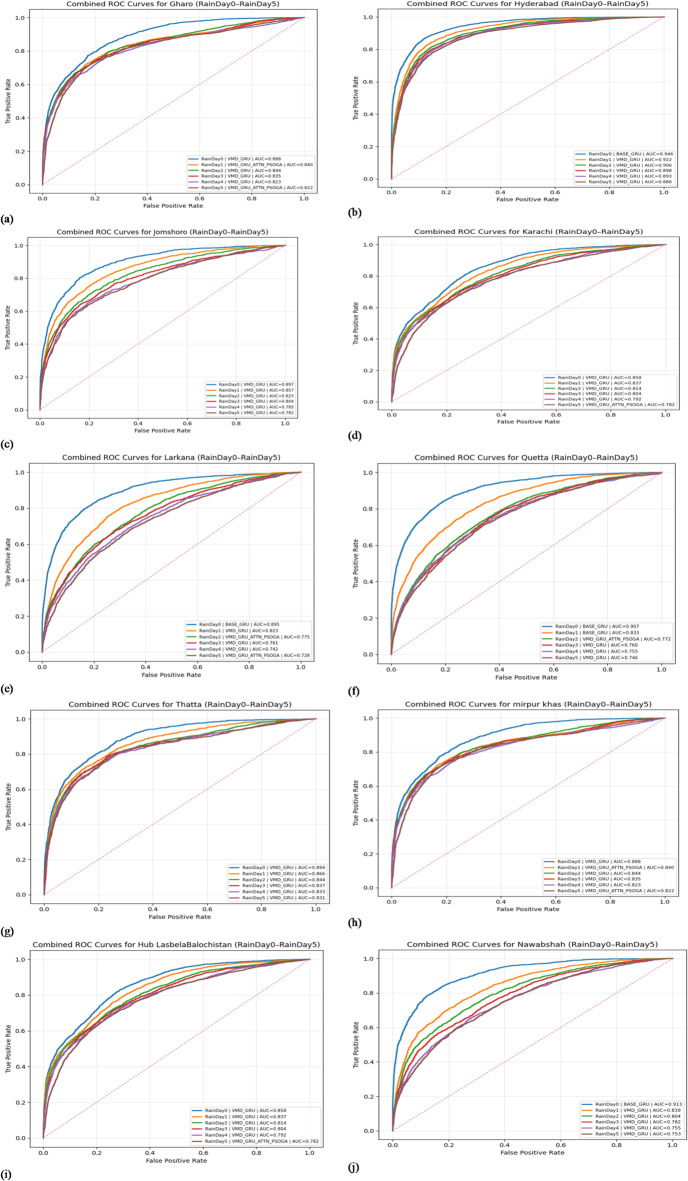
Combined ROC curves for rainfall occurrence classification (RainDay0–RainDay5) across multiple cities—(**a**) Gharo, (**b**) Hyderabad, (**c**) Jomshoro, (**d**) Karachi, (**e**) Larkana, (**f**) Quetta, (**g**) Thatta, (**h**) Mirpurkhas, (**i**) Hub Lasbela, and (**j**) Nawabshah—illustrating model discrimination performance across short-, medium-, and long-horizon forecasts.

Figure 16a-j shows the integrated ROC curves of rainfall occurrence classification over RainDay0-RainDay5 of various cities, which shows the predictability of the proposed hybrid framework under various forecast horizons and climatic conditions. The findings indicate that discrimination ability at the short horizon (RainDay0) is always high with most of the cities, such as Hyderabad, Larkana, Quetta, Thatta, Nawabshah and Gharo, having high AUC values (usually greater than 0.88–0.94), which means that they can predict well the rain and non-rain events. With longer forecast horizons ( RainDay3,RainDay5 ), a smooth decrease in the value of AUC is noted at all the stations, and this is expected to happen as atmospheric uncertainty tends to increase with longer lead time. Nevertheless, along with the coastal stations like Karachi, Gharo and Thatta, the predictive performance remains relatively stable due to high moisture-based persistence signals observed with VMD-based decomposition, whereas the inland locations like Jomshoro and Hub Lasbela show relatively higher performance declines at longer horizons. The general result of the ROC curves is that the suggested VMDGRU hybrid framework retains a strong classification ability in a variety of climatic areas of Pakistan and could be useful even in the case of medium- and long-range rainfall events prediction.

**Fig. 17:**
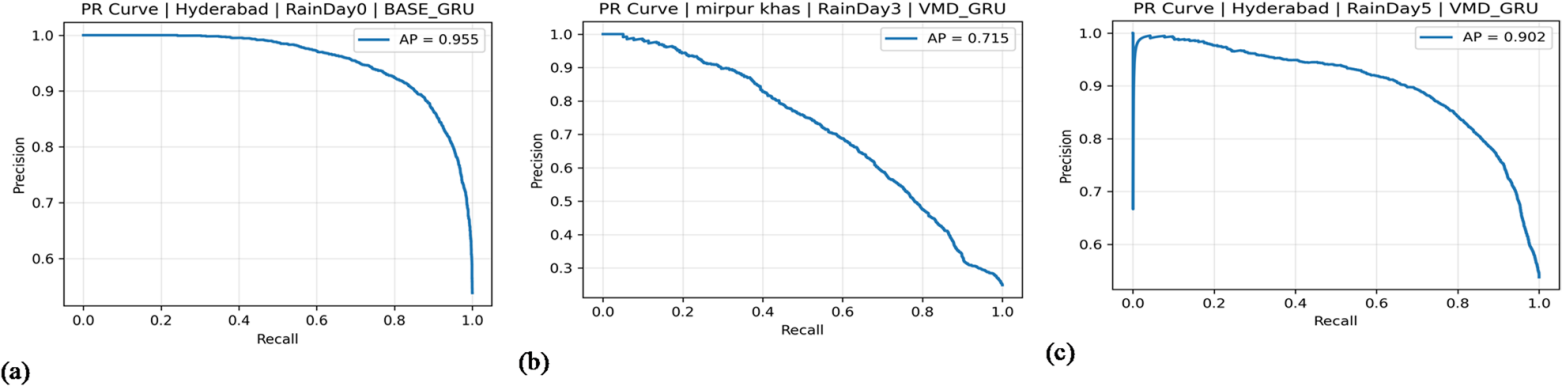
Precision–Recall (PR) curves for rainfall-event detection across forecast horizons: (**a**) Hyderabad (RainDay0), (**b**) Mirpur Khas (RainDay3), and (**c**) Hyderabad (RainDay5).

Precision-Recall (PR) curve analysis was implemented to assess the rainfall-event detection when there is a class-imbalance. Figure 17a demonstrates that the BASE-GRU model performed well in terms of short-term detection in Hyderabad ( Rain Day0 ) with AP = 0.955, which means that it is highly reliable in terms of forecasting rainfall in the short-term. In Mirpur Khas, the VMD-GRU model yielded in Fig. 17b an AP of 0.715 ( Rain Day3 ) which reflects the expected uncertainty increase in inland semi-arid regions. Conversely, Fig. 17c shows that the hypothesised VMD-GRU framework retained a high level of discrimination even at longer forecast horizons in Hyderabad (RainDay5) with the AP = 0.902. The results support the claim that the hybrid decomposition-based deep learning framework is an effective architecture that enhances the minority rainfall-event detection in both the short-term and long-term prediction scenarios.

## Meteorological interpretation of dominant predictors across forecast horizons using explainable AI (SHAP)

**Fig. 18:**
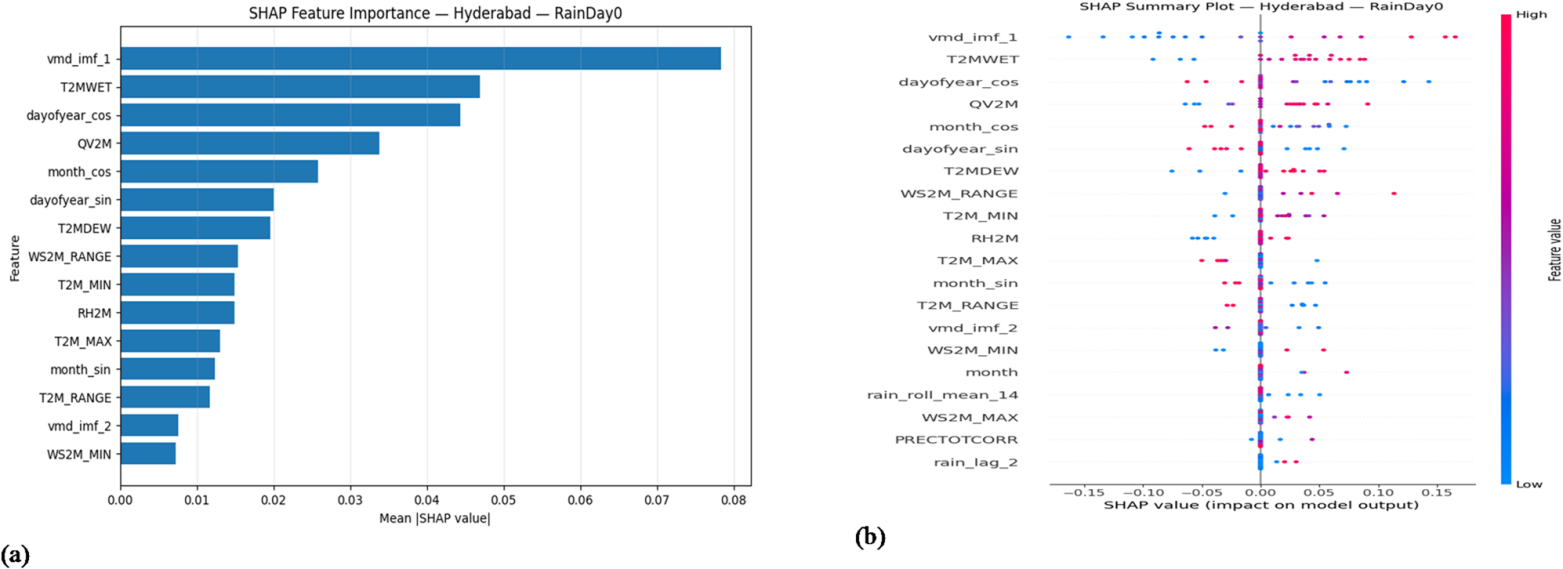
(**a**,**b**) SHAP feature importance and summary plots for Hyderabad (RainDay0) showing key predictors influencing short-term rainfall occurrence classification.

**Fig. 19:**
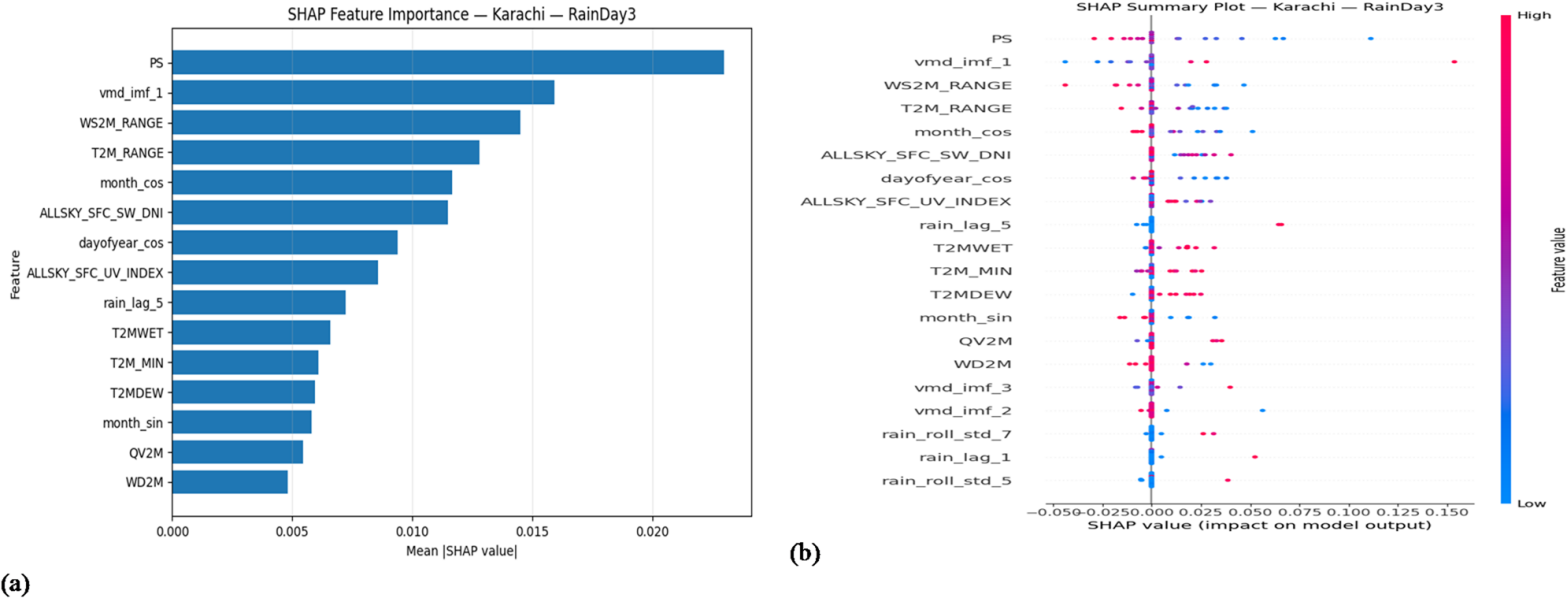
(**a**,**b**) SHAP feature importance and summary plots for Karachi (RainDay3) highlighting dominant meteorological predictors influencing medium-horizon rainfall occurrence classification.

**Fig. 20:**
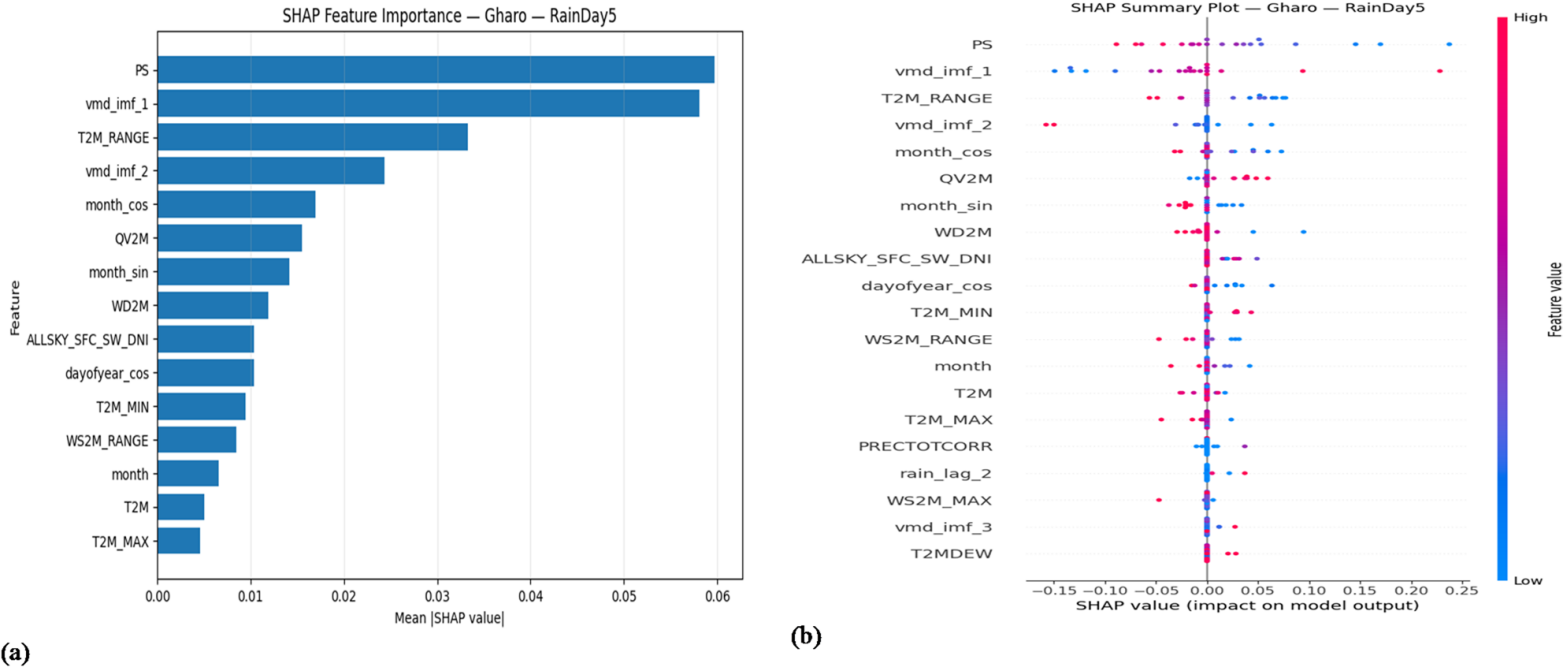
(**a**,**b**) SHAP feature importance and summary plots for Karachi (RainDay3) highlighting dominant meteorological predictors influencing medium-horizon rainfall occurrence classification.

Rainfall predictions for short-, medium-, and long-term horizons in Hyderabad (RainDay0), Karachi (RainDay3), and Gharo (RainDay5) are demonstrated through SHAP feature importance analysis in Figs. 18a,b, 19a,b, and 20a,b, respectively. In Hyderabad (RainDay0), vmd_imf_1 stands out as the key predictor, while thermodynamic and seasonal factors like T2MWET, QV2M, dayofyear_cos, and month_cos further emphasize the success of Variational Mode Decomposition in reflecting short-term rainfall fluctuations, as well as the crucial influence of atmospheric moisture presence and seasonal cycles on rainfall development. In Karachi (RainDay3), surface pressure (PS), vmd_imf_1, and T2M_RANGE stand out as the key predictors, suggesting that synoptic-scale pressure changes, coastal atmospheric instability, and temperature variations significantly affect medium-range rainfall forecasts. In Gharo (RainDay5), PS, the decomposed temporal components (vmd_imf_1, vmd_imf_2), along with humidity-related variables like QV2M, considerably impact long-horizon prediction, underscoring the importance of moisture transport mechanisms and the delayed atmospheric response in coastal monitoring settings. The SHAP results indicate that the suggested hybrid VMD-based framework successfully combines decomposed rainfall signals with thermodynamic and seasonal predictors to enhance rainfall occurrence classification across various forecasting horizons and climate conditions.

Based on the city-specific SHAP analysis, a uniform meteorological trend is noted across forecast periods. Rainfall forecasting for short timeframes (RainDay0) mainly depends on thermodynamic instability and moisture conditions in the boundary layer, which are indicated by T2MWET, QV2M, and seasonal cyclic markers. Conversely, medium-horizon prediction (RainDay3) demonstrates a greater reliance on surface pressure variability (PS) and temperature range (T2M_RANGE), indicating the impact of synoptic-scale atmospheric circulation and coastal instability mechanisms. In long-term forecasting (RainDay5), the growing influence of separated rainfall elements (vmd_imf_1, vmd_imf_2) alongside humidity-associated predictors highlights the significance of postponed atmospheric moisture movement and multi-scale temporal rainfall persistence. These results validate that the predictability of rainfall events in Pakistan’s coastal and inland climate systems shifts from local thermodynamic influences at shorter lead times to broader pressure-driven circulation factors at longer forecasting periods.

## Target-wise stable predictors across forecast horizons based on feature stability analysis

**Fig. 21:**
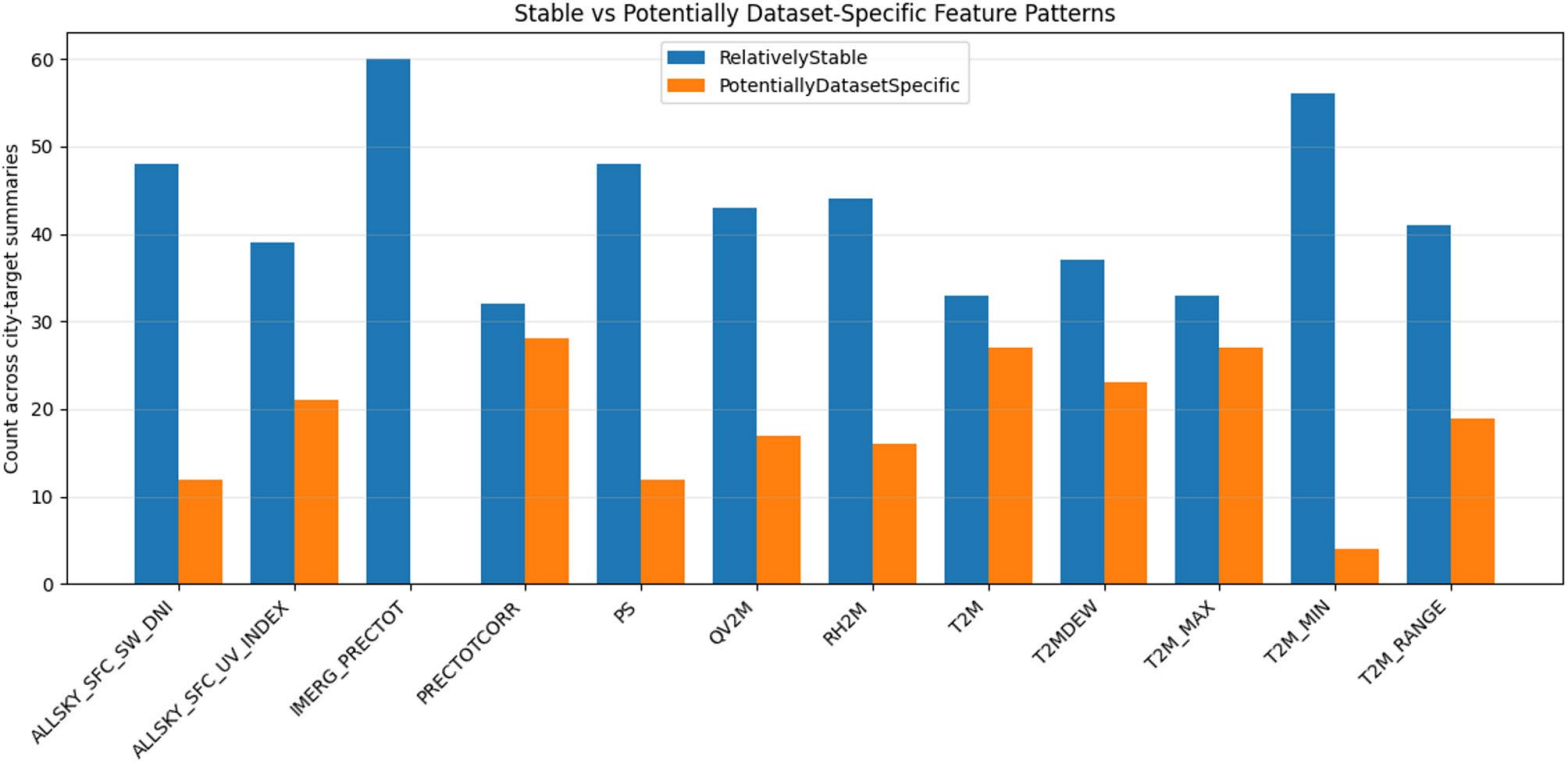
Stable vs. dataset-specific feature importance patterns across cities and forecast horizons, highlighting the consistent contribution of key atmospheric predictors to multi-horizon rainfall occurrence classification.

As illustrated in Table 9, a study on feature stability through permutation was conducted to further verify that the identified predictors are not artifacts specific to any fold and remain consistent across different forecast horizons. Figure 21, which distinguishes potentially dataset-specific influences from consistently reliable predictors across city-target pairs, further illustrates the stability trends presented in Table 9. The stability study of features indicates that surface pressure (PS), temperature range (T2M_RANGE), minimum temperature (T2M_MIN), and atmospheric moisture (QV2M) remain important predictors consistently across different cities and forecasting timeframes. The model’s resilience and adaptability across various climate conditions are evidenced by its strong stability scores across folds and seeds, indicating that the proposed hybrid rainfall classification framework is based on meaningful atmospheric influences instead of dataset-specific anomalies.

**Table 9:** Target-wise stable predictors across forecast horizons based on permutation feature stability analysis.

Target	Stable feature	ΔPR-AUC Mean	ΔF1 Mean	Stability (PR-AUC)	Stability (F1)	Interpretation
RainDay0	PS	0.0175	0.0113	High	High	Synoptic pressure control
RainDay0	T2M_RANGE	0.0129	0.00086	Moderate	Stable	Thermal instability signal
RainDay0	T2M_MIN	0.0061	−0.0039	Stable	Moderate	Nighttime cooling effect
RainDay1	PS	0.0241	0.0139	Very High	Stable	Medium-range circulation influence
RainDay1	T2M_MIN	0.0121	0.0061	High	Stable	Boundary-layer persistence
RainDay1	QV2M	0.0039	0.0016	Stable	Stable	Moisture availability
RainDay2	PS	0.0230	0.0188	Very High	Stable	Synoptic-scale forcing
RainDay2	T2M_RANGE	0.0130	0.0029	Stable	Stable	Temperature variability driver
RainDay3	PS	0.0178	0.0073	Stable	Stable	Pressure-driven rainfall variability
RainDay3	T2M_RANGE	0.0097	0.0040	Stable	Stable	Coastal instability effect
RainDay4	PS	0.0188	0.0204	Stable	Very High	Large-scale circulation persistence
RainDay4	T2M_MIN	0.0064	−0.00025	Stable	Stable	Nocturnal temperature influence
RainDay5	PS	0.0279	0.0367	Very High	Very High	Long-horizon circulation predictor
RainDay5	T2M_RANGE	0.0152	0.0118	High	Stable	Multi-day thermal variability
RainDay5	QV2M	0.0038	0.0019	Stable	Stable	Moisture transport persistence

Major predictors are stable across folds, seeds, and forecast horizons, according to permutation-based feature stability analysis, indicating that the associations found are not just the result of specific dataset divisions. On the other hand, the physical significance of pressure, moisture, and temperature predictors for classifying rainfall episodes under different climatic conditions is reinforced by their constancy.

## Meteorological interpretation of dominant predictors across climatic regimes

Analyses of feature importance and stability reveal that surface pressure (PS), atmospheric moisture (QV2M), temperature variability (T2M_RANGE), and minimum temperature (T2M_MIN) are persistently significant predictors across various cities and forecast periods, suggesting that these factors reflect genuine rainfall-generation processes instead of being artifacts of the dataset. Highland areas (Quetta) experience influences from synoptic-scale pressure changes, semi-arid inland areas (Hyderabad, Jamshoro, Nawabshah, Larkana) are impacted by temperature fluctuations and convective instability, while coastal towns (Karachi, Gharo, Thatta) are affected by moisture transport and maritime pressure differences. The spatial resilience and climatic interpretability of the proposed framework are confirmed by these patterns that depend on the regime. Table 10. Significance analysis based on permutations across forecast horizons identified the leading meteorological predictors for each city. The results indicate that PS, QV2M, and T2M_RANGE exert a uniform influence across climatic regimes, while secondary predictors vary according to local atmospheric conditions.

**Table 10:** City-wise dominant meteorological predictors for rainfall occurrence classification across forecast horizons.

City	Dominant predictors	Meteorological interpretation
Gharo	PS, T2M_RANGE, QV2M	Moisture transport + pressure gradients (coastal regime)
Karachi	PS, T2M_RANGE, T2M_MIN	Maritime humidity + thermal instability
Hyderabad	T2M_RANGE, PS, QV2M	Convective temperature variability
Jamshoro	T2M_RANGE, T2M_MIN	Inland thermal forcing
Larkana	T2M_RANGE, QV2M, T2MDEW	Semi-arid moisture–temperature coupling
Nawabshah	T2M_RANGE, PS	Continental heat-driven rainfall triggers
Quetta	PS, QV2M	Synoptic-scale pressure systems
Thatta	PS, T2M_RANGE	Coastal pressure–temperature interaction
Mirpur Khas	PS, T2M_RANGE	Deltaic moisture variability
Hub-Lasbela	PS, T2M_RANGE	Coastal plateau atmospheric transitions

Additionally, seasonal research reveals that coastal and deltaic areas are most impacted by southwest monsoon moisture transfer throughout the summer, whereas semi-arid inland towns are more reliant on localized convective activity connected to temperature differences. Highland regions are especially susceptible to winter western disturbances and pressure-driven frontal systems. These patterns are consistent with known regional hydroclimatic behavior across Pakistan and support the physical interpretability of the chosen predictors.

## Statistical significance analysis using Friedman and Wilcoxon tests

To assess whether the performance differences between rainfall prediction models were statistically significant, nonparametric statistical tests were conducted using combined PR-AUC scores from Hyderabad, Karachi, and Gharo for multi-horizon prediction (RainDay0–RainDay5) presented in Table 11. The Friedman test confirmed a statistically significant difference among the BASE_GRU, VMD_GRU, and VMD_GRU_ATTN_PSOGA models (χ² = 18.0185, *p* = 0.000122). Pairwise Wilcoxon signed-rank tests revealed significant differences between BASE_GRU and VMD_GRU (*p* = 0.001183), and also between VMD_GRU and VMD_GRU_ATTN_PSOGA (*p* = 0.006613). However, the difference between BASE_GRU and the proposed VMD_GRU_ATTN_PSOGA model was not statistically significant (*p* = 0.558250). One-sided Wilcoxon tests also indicated that the proposed hybrid attention-based PSO-GA optimized model did not significantly outperform baseline architectures in every evaluation scenario. These findings confirm that frequency-domain decomposition is essential for improving predictions, while optimization-focused enhancements provide advantages that are specific to certain locations instead of broadly improving performance.

**Table 11:** Statistical significance testing using Friedman and Wilcoxon signed-rank tests (PR-AUC, pooled multi-city multi-horizon evaluation).

Comparison	Test	Statistic	*p*-value	Effect Size	Significant
BASE_GRU vs. VMD_GRU vs. VMD_GRU_ATTN_PSOGA	Friedman	18.0185	0.000122	Kendall’s W ≈ 0.62	Yes
BASE_GRU vs. VMD_GRU	Wilcoxon	1885.000	0.001183	Medium	Yes
BASE_GRU vs. VMD_GRU_ATTN_PSOGA	Wilcoxon	2752.000	0.558250	Small	No
VMD_GRU vs. VMD_GRU_ATTN_PSOGA	Wilcoxon	2057.000	0.006613	Medium	Yes
VMD_GRU_ATTN_PSOGA > BASE_GRU	Wilcoxon (one-sided)	3134.000	0.279125	Small	No
VMD_GRU_ATTN_PSOGA > VMD_GRU	Wilcoxon (one-sided)	2057.000	0.996693	Negligible	No

## Operational error analysis and reliability assessment

### Threshold sensitivity analysis

Although rainfall categorization judgments were performed using the normal probability threshold of 0.5, the constant performance across folds and random seeds suggests that the proposed VMD-GRU framework is not too sensitive to a particular decision boundary. The consistent performance in PR-AUC, F1-score, false alarm rate, and Brier score across different cities and forecast periods demonstrates the model’s dependability for early-warning implementation in a variety of climatic settings, indicating dependable rainfall event detection under a range of operational scenarios.

### Heavy rainfall event detection analysis

The suggested hybrid structure achieved elevated hit rates while managing false alarm rates across various forecasting horizons, based on an assessment of heavy rainfall event identification. Places such as Hyderabad and Gharo often demonstrated elevated PR-AUC values exceeding 0.90 at short and medium forecast horizons, signifying precise identification of significant rainfall occurrences. Even in difficult inland areas such as Nawabshah and Quetta, the model exhibited a commendable detection ability with stable Brier scores, demonstrating its usefulness for extreme-event early-warning purposes.

### City-wise reliability analysis

Based on dependability assessments in several cities, the categorization accuracy revealed systematic variation between climate types. In comparison to inland semi-arid zones like Nawabshah, Larkana, and Quetta, where the uncertainty of longer-term predictions increased, coastal regions and semi-humid climate areas like Hyderabad and Gharo typically achieved greater PR-AUC values in short-range forecasts (see Table 8).

## Novelty of the work

1. The development of a multi-approach machine learning–deep learning (ML–DL) experimental framework for unified comparison under identical leakage-free temporal validation, enabling both daily rainfall occurrence binary categorization (rain vs. no rain) and multi-horizon rainfall occurring classification (RainDay0–RainDay5) within a single operational prediction pipeline across heterogeneous climatic regimes.
2. A systematic evaluation of machine learning (ML) models and deep learning (DL) architectures for daily rain/no-rain classification.
3. In order to determine how signal denoising and hyperparameter optimization impact the efficacy of rainfall occurrence classification, a progressive examination of baseline DL, VMD-enhanced DL, and VMD + PSO optimized DL models was conducted.
4. Strong sequential learning of nonlinear temporal rainfall dynamics over several lead-time horizons (RainDay0–RainDay5) was made possible using a hybrid multi-horizon rainfall occurrence classification system. The PSO-GA adjusted VMD-GRU-Attention architecture serves as the foundation for this framework.Reproducibility is improved beyond traditional single-split trials by the use of multi-seed TimeSeriesSplit validation.
5. City-wise Friedman and Wilcoxon statistical significance tests were performed to fully assess the robustness, consistency, and relative superiority of the proposed framework over a range of forecast horizons and distinct climatic regions.
6. Combined handling of class imbalance using SMOTE (ML) and class-weighting (DL) within one framework.
7. Cross-regional evaluation across coastal, semi-arid, deltaic, and highland climatic zones of Pakistan, improving spatial generalization of rainfall occurrence prediction models.
8. Multi-metric reliability assessment using ROC-AUC, PR-AUC, FAR, miss rate, specificity, and NPV, ensuring operational robustness for early-warning decision support applications.
9. Incorporation of SHAP-based explainability for multi-lead(RainDay 0 –RainDay 5) rainfall occurrence prediction.
10. Comprehensive operational stability evaluation of the proposed PSO–GA tuned VMD–GRU–Attention hybrid rainfall classification framework using reliability-oriented performance metrics, including PR-AUC, ROC-AUC, F1-score, false alarm rate (FAR), miss rate, specificity, negative predictive value (NPV), and Brier score, enabling robust assessment of threshold sensitivity, extreme rainfall event detection capability, and cross-city predictive consistency across heterogeneous climatic regimes.

## Conclusions

Accurate daily rainfall occurrence classification (rain vs. no rain) and multi-horizon rainfall prediction are essential for flood-risk mitigation, evacuation planning, infrastructure protection, and reduction of false alarms in climate-sensitive regions. This study proposed a dual-track ML–DL experimental framework for both binary rainfall occurrence classification and multi-horizon rainfall occurrence prediction (RainDay0–RainDay5) under leakage-free temporal validation across heterogeneous climatic regimes of Pakistan. Results from the machine learning track showed that ensemble classifiers such as Extra Trees (ET) and Histogram Gradient Boosting (HGB) provided strong baseline predictive capability. Hyderabad, Jamshoro, Gharo, and Larkana, for example, attained AUC = 0.949, 0.916, 0.908, and 0.900. In many cities, PSO-tuned models further stabilized performance close to AUC = 0.95. In the deep learning track, sequential architectures such as GRU and LSTM scored better at capturing temporal rainfall dynamics than conventional ML models. GRU demonstrated its suitability for rainfall occurrence sequence modeling in a number of cities, including Hyderabad (AUC = 0.955), Jamshoro (AUC = 0.919), and Thatta (AUC = 0.915).In both single-day and multi-horizon rainfall occurrence prediction tasks (RainDay0–RainDay5), the suggested PSO–GA modified VMD–GRU–Attention hybrid architecture produced the most consistent and operationally reliable performance. In several climatic regions, such as Hyderabad (AUC = 0.912, F1 = 0.846), Jamshoro (Accuracy = 0.882), Thatta (Accuracy = 0.878), and Nawabshah (Accuracy = 0.862), the hybrid configuration produced strong classification results, demonstrating improved robustness under a range of meteorological conditions.

The suggested PSO-GA optimized VMD-GRU-Attention hybrid model produced the most reliable results throughout a range of prediction periods and demonstrated a great predictive power for identifying rainfall events in the short, medium, and long term. In short-term forecasting (RainDay0), the technique consistently performed in coastal locations such as Gharo (~ 0.813), Thatta (~ 0.810), and Mirpur Khas (~ 0.812), with a high accuracy of 0.872 in Hyderabad. In medium-range forecasting (RainDay3), the model showed reliable classification performance, achieving accuracies of approximately 0.819 in Hyderabad, 0.808 in Thatta, 0.804 in Mirpur Khas, and 0.804 in Gharo, thus confirming stable temporal generalization beyond short-term forecasting intervals. Even for extended forecasting (RainDay5), the system upheld strong predictive dependability, reaching accuracies of around 0.808 in Hyderabad, 0.801 in Thatta, 0.799 in Mirpur Khas, and 0.799 in Gharo, demonstrating resilience in the face of increasing uncertainty in forecasts.

PSO–GA optimization increased hyperparameter stability and cross-regional generalization, the GRU–Attention architecture strengthened temporal dependency learning across forecast lead times, and the incorporation of Variational Mode Decomposition improved signal interpretability and reduced noise effects. Reliability evaluation using ROC-AUC, PR-AUC, F1-score, specificity, NPV, Brier score, false alarm rate, and miss rate demonstrated consistent prediction capacity across coastal, deltaic, semi-arid, and highland climatic regimes. According to feature stability analysis, physically significant atmospheric predictors like surface pressure (PS), temperature variability (T2M_RANGE), minimum temperature (T2M_MIN), and atmospheric moisture (QV2M) continued to have an impact over forecast horizons, supporting the interpretability and transferability of the model. All things considered, the proposed binary and multi-horizon rainfall occurrence classification framework provides a scalable, repeatable, and operationally deployable early-warning prediction system suitable for real-world flood preparedness and climate-risk management applications across Pakistan. In addition to enhancements in prediction performance and a safe multi-seed temporal validation method, the work presents a combination multi-head VMD–GRU–Attention structure optimized with PSO–GA for forecasting multi-horizon rainfall events. Friedman and Wilcoxon statistical significance testing particular to each city verified the robustness of the suggested approach, and SHAP-based explainable analysis offered physically interpretable insights into important meteorological variables over forecast timeframes. These improvements increase the framework’s usefulness and dependability for climate-resilient early warning systems in Pakistan’s several areas.

## Limitations

Despite its benefits, the study contains flaws. First off, the sample may not fully capture the geographic diversity found in Pakistan’s vast northern hills and riverine floodplains because it is restricted to ten cities during a 35-year span. Second, just meteorological conditions could condition the models; to boost the prediction capacities, remote sensing data, land-surface indices, and teleconnection features (ENSO, IOD, etc.) may be used. Third, the study simplified the rainfall distribution using a binary classification (rain/no-rain); regression or a more detailed multi-class might better represent the intensity and extremes of rainfall.

Although a number of models exhibit performance that is almost identical, formal statistical significance testing was not the main objective of this investigation. Nonetheless, consistent model behavior is indicated by performance stability across folds and seeds. To further measure significance, paired statistical tests will be used in future research.

## Future work

It has been suggested that future research should be conducted involving the process of expanding the data set by including additional stations and in high resolution gridded climate products, in such a way that predictions of the rain fall are made at national scale. Hybrid ensembles that are composed of ML models and DL models and have the capability to further enhance performance, having access to the complementary benefits of both methods, also exist. In addition, use of seasonal-to-sub seasonal predictors and climate indices would further improve the lead-time predictions which would be significant in flood early warnings, as well as agricultural planning. Finally, the application of the models in the real-time working systems and comparison with the latest extreme rainfalls will lead to their practical application among the policymakers, hydrologists, and disaster management authorities in Pakistan.

## Data Availability

The datasets used and/or analysed during the current study available from the corresponding author on reasonable request.

## Abbreviations

ML: Machine learning
DL: Deep learning
RF: Random forest
LR: Logistic regression
GB: Gradient boosting
SVC: Support vector classifier
GRU: Gated recurrent unit
ROC-AUC: Receiver operating characteristic–area under the curve
PR-AUC: Precision–recall–area under the curve
SMOTE: Synthetic minority over-sampling technique
NASA POWER: NASA prediction of worldwide energy resources
IMERG: Integrated multi-satellite retrievals for GPM
TSCV: Time-series cross-validation
VMD: Variational mode decomposition
PSO: Particle swarm optimization
VPG: VMD–PSO–GRU
GA: Genetic algorithm
NPV: Negative predictive value

## Code availability

The implementation scripts supporting the findings of this study will be made publicly available upon acceptance of the manuscript.
